# Synthesis and Structure–Activity Relationship of Palmatine Derivatives as a Novel Class of Antibacterial Agents against *Helicobacter pylori*

**DOI:** 10.3390/molecules25061352

**Published:** 2020-03-16

**Authors:** Tianyun Fan, Xixi Guo, Qingxuan Zeng, Wei Wei, Xuefu You, Jing Pang, Yanxiang Wang, Danqing Song

**Affiliations:** 1 Beijing Key Laboratory of Antimicrobial Agents, Institute of Medicinal Biotechnology, Chinese Academy of Medical Sciences and Peking Union Medical College, Beijing 100050, China; fty1668@163.com (T.F.); sissi.kwok@outlook.com (X.G.); zqx50810793@163.com (Q.Z.); weiwei082695@163.com (W.W.); 13311123098@163.com (X.Y.); songdanqingsdq@hotmail.com (D.S.); 2 State Key Laboratory of Bioactive Substance and Function of Natural Medicines, Institute of Materia Medica, Chinese Academy of Medical Sciences and Peking Union Medical College, Beijing 100050, China

**Keywords:** *Helicobacter pylori*, palmatine, synthesis, structure–activity relationship, urease

## Abstract

Taking palmatine (PMT) as the lead, 20 new PMT derivatives were synthesized and examined for their antibacterial activities against six tested metronidazole (MTZ)-resistant *Helicobacter pylori* (*H. pylori*) strains. The structure–activity relationship (SAR) indicated that the introduction of a suitable secondary amine substituent at the 9-position might be beneficial for potency. Among them, compound **1c** exhibited the most potent activities against MTZ-resistant strains, with minimum inhibitory concentration (MIC) values of 4–16 μg/mL, better than that of the lead. It also exhibited a good safety profile with a half-lethal dose (LD_50_) of over 1000 mg/kg. Meanwhile, **1c** might exert its antimicrobial activity through targeting *H. pylori* urease. These results suggested that PMT derivatives might be a new family of anti-*H. pylori* components.

## 1. Introduction

*Helicobacter pylori* (*H. pylori*), spiral shaped gram-negative bacteria, has infected more than 50% of humans globally. It is believed that gastrointestinal diseases, such as gastritis and peptic ulcer, were highly related to *H. pylori* infections [1]. Furthermore, the damage to gastric structure and function might lead to the development of gastric cancer, which becomes a major cause of morbidity and mortality worldwide [2,3]. *H. pylori* has been classified as a class I carcinogen by the World Health Organization (WHO) [4]. Currently, triple therapy regimens comprising amoxicillin and metronidazole (MTZ) or clarithromycin, as well as proton pump inhibitor, are recommended as first-line treatment for *H. pylori* infections [5,6]. However, the prevalence of antibiotic resistance, including MTZ and clarithromycin [3], decreased the efficacy and has become a great challenge to the clinical treatment. Thus, the discovery and development of novel agents against *H. pylori* to eradicate the pathogen is still of great importance.

**Figure 1:**
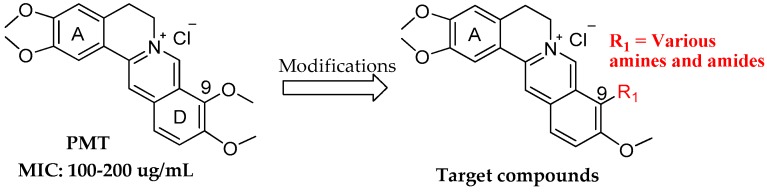
Chemical structure of palmatine (PMT), and structure modification strategy. MIC, minimum inhibitory concentration.

Our group has been committed to exploring antibacterial agents with unique structure skeletons and novel mechanisms, and extensive efforts have been made on structural modifications and antibacterial activity explorations of protoberberine alkaloid [7,8,9,10]. Palmatine (PMT, Figure 1), a representative protoberberine alkaloid extracted from the traditional Chinese medicine *Coptis chinensis*, possesses a special quaternary ammonium in its structure and has diverse pharmacological and biological activities, including anti-inflammatory, anti-cancer, antiviral, and neuro-protection [11,12,13]. Meanwhile, PMT and its derivatives showed a potential as antibacterial candidates with their antibacterial efficacy against both gram-positive and gram-negative bacteria (including *Staphylococcus aureus*, *Escherichia coli*, and so on), which has been disclosed by different groups [14,15,16].

*H. pylori* creates a local neutral environment for survival by continuously releasing ammonia into *succus gastricus*, mediated by its own urease. Therefore, urease plays an essential role for *H. pylori* colonization in the human stomach, and is considered to be a critical target in the exploitation of anti-*H. pylori* agents [17,18,19,20]. Until now, no drug has been approved by Food and Drug Administration (FDA) targeting *H. pylori* urease [20,21]. As a novel *H. pylori* urease inhibitor, PMT exhibits bactericidal activity against *H. pylori* both in vitro and in vivo [22,23], as well as its potential therapeutic effects on gastritis and peptic ulcer caused by *H. pylori* infection, and other urea-related diseases have also been reported [24,25]. However, PMT only exhibited moderate potency with minimum inhibitory concentration (MIC) values of 100 to 200 μg/mL, which need further improvement. Besides, the anti-*H. pylori* activity and structure–activity relationship (SAR) of its derivatives have not been investigated. Therefore, it was essential to conduct structural modification and anti-*H. pylori* activity screening on its kind, so as to acquire potent anti-*H. pylori* candidates.

As ionizable nitrogen is beneficial for the accumulation of small-molecule antibacterial in gram-negative bacteria [26], SAR study was mainly focused on the substituent at the 9-posision in the present study. Twenty new 9-*N* substituted PMT derivatives were prepared and evaluated for their anti-*H. pylori* activity, as depicted in Figure 1, taking PMT as the lead. Meanwhile, SAR analysis, safety evaluation and preliminary mechanism exploration of the representative compound are also described.

## 2. Results and Discussion

### 2.1. Chemistry

**Scheme 1:**
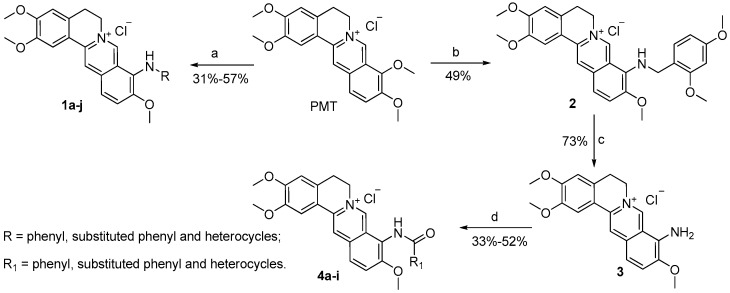
Reagents and conditions: (**a**) RNH_2_, 95–120 °C, 4–72 h; (**b**) 2,4-dimethoxybenzylamine, 110 °C, 6 h; (**c**) 1:1 HCl/CH_3_OH, room temperature (rt), 24 h; (**d**) R_1_COCl, pyridine, CH_3_CN, 71 °C, 3–24 h.

The synthetic route used for the preparations of all amine and amide derivatives of PMT is presented in Scheme 1. PMT was first heated with amines, which were used as the nucleophilic reagents and the solvents, to produce the corresponding amine derivatives **1a**–**j** and **2** in 31–57% yields [27,28]. Subsequently, compound **3** with a free amine was obtained using **2** and HCl/CH_3_OH (1:1 by vol) as the reactive reagents with the yield of 73%. Afterward, taking **3** and corresponding acyl chlorides as the reactive materials, the target products **4a**–**i** were acquired in 33–52% yields [27]. All the target compounds were purified via flash column chromatography on silica gel using CH_2_Cl_2_ and MeOH as eluent or washed with CH_2_Cl_2_ and 80% EtOH.

### 2.2. Pharmacological Evaluation

The European Committee on Antimicrobial Susceptibility Testing (EUCAST) resistance breakpoint of MTZ to *H. pylori* was >8 μg/mL [29,30]. We carried out the SAR study for antibacterial activity against six different MTZ-resistant *H. pylori* strains including ATCC43504 and five clinical isolated strains from Chinese hospitals, taking PMT and MTZ as the positive controls. The chemical structures of 20 target compounds and their MIC values are depicted in Table 1.

As a start, a benzyl amine group was attached on position 9 and compound **1a** was constructed. It exhibited comparable potencies against all the tested MTZ-resistant strains compared with those of positive controls. Subsequently, compounds **1b**–**d** and **2** were designed to explore the influence of electron-donating groups, including methyl and methoxy on the benzyl group. Among them, compounds **1b**, **1c**, and **2** with m-methyl, p-methyl, and *o*,*p*-methoxy benzyl amine substituent displayed better potencies than those of PMT and MTZ against all tested strains, with MIC values of 4–32 μg/mL. However, compound **1e** with the electron-withdrawing group cyano-substituted lost its antibacterial activity. These results hinted that electron-donating groups on the benzyl group might be beneficial for antibacterial potency. Then, the fluorine and bromine atom, as the bioisosteres of the hydrogen atom, were attached on the benzyl amine substituent and **1f**–**g** were created. All of them showed comparable activities to that of PMT and MTZ. After that, heterocyclic amine derivatives **1h** and **1i** were synthesized and screened, and compound **1h** acquired improved potencies than those of positive controls. Finally, the efficacy of compound **3** with 9-amine substituted was eliminated totally, which suggested that appropriate substituted secondary amine on position 9 might be helpful for maintaining potency against MTZ-resistant *H. pylori* strains.

As a comparison, substituted amide analogues **4a**–**i** possessing less ionizable nitrogens on position 9 were designed and screened for their anti-*H. pylori* activities. As shown in Table 1, all of them lost the anti-*H. pylori* potencies partially or completely, suggesting that appropriate ionizable nitrogen might be essential for the activity.

Although different candidates are in various phases of discovery and development pipeline [17,18,19,20], no drugs with novel skeleton against *H. pylori* have been approved recently. Compound **1c** with unique skeleton exhibited most the promising potency against *H. pylori* compared with PMT, and was selected as the representative compound for further investigation.

### 2.3. Acute Toxicity Assay of Compound ***1c***

Then, taking Kunming mice as the animal model, we performed an acute toxicity test on compound **1c** through oral administration. All mice survived during the seven-day observation period, and had glossy hair, fleshy body, agile movement, and good appetite. The result indicated that the half-lethal dose (LD_50_) value of compound **1c** was over 1000 mg/kg, suggesting a good safety feature.

### 2.4. Molecular Docking Study of Key Compound ***1c***

*H. pylori* expresses high levels of urease, which enables the survival and colonization of itself. To further explore the preliminary mechanism of compound **1c**, molecular docking studies were conducted to calculate the interactions between compound **1c** and PMT with *H. pylori* urease (Protein Data Bank (PDB): 1E9Y) [31], respectively. The LibDock score of compound **1c** (104.13) with *H. pylori* urease was higher than that of PMT (92.14), which is consistent with the phenotype screening results.

**Figure 2:**
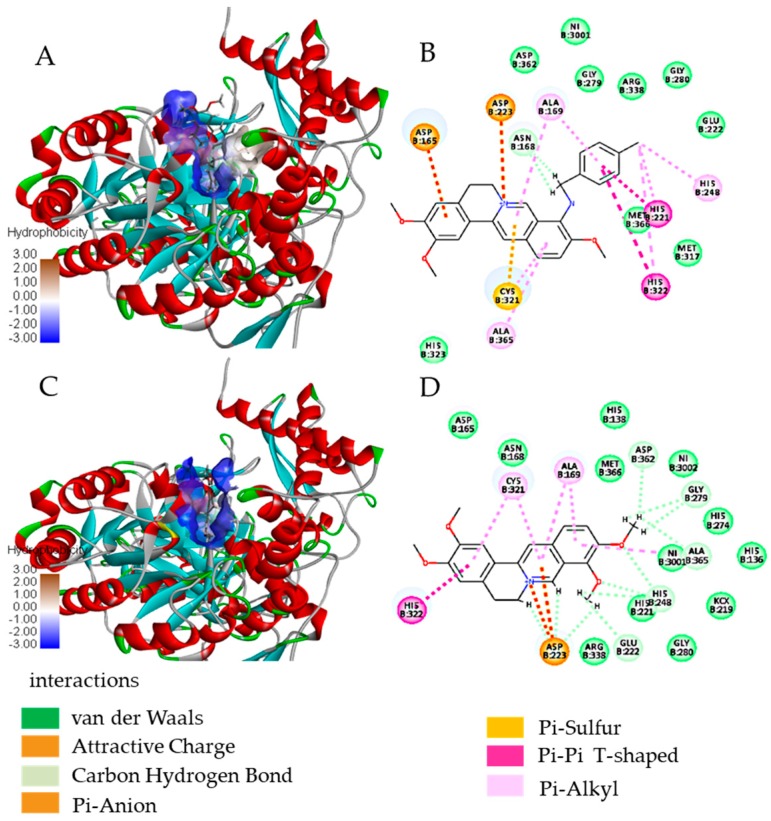
Solid surface maps of the active pocket of *H. pylori* urease with compound **1c** (**A**) and PMT (**C**). Red, blue, and white colored regions correspond to negatively charged, positively charged, and neutral areas, respectively. Binding mode plans of **1c** (**B**) and PMT (**D**) within the *H. pylori* urease active pocket. These figures were produced using the Discovery Studio 4.5 software. Van der Waals force, green; attractive charge force, orange; carbon hydrogen bond force, celadon; Pi-Anion force, orange; Pi-sulfur force, yellow; Pi-Pi T-shaped force, amaranth; Pi-alkyl force, mauve pink. The receptor structure is shown in surface form.

The docking results of *H. pylori* urease with PMT and compound **1c** are illustrated in Figure 2. Both of them could embed into the active hydrophobic pocket of *H. pylori* urease and fit well in the binding site (Figure 2A,C). Besides attractive charges and Pi-Alykl, several other typical interactions between **1c** and *H. pylori* urease, including Pi-Pi T-shaped with residue HIS-221 and Pi-Anion with residue ASP-165, were predicted (Figure 2B), which together contributed to the stronger interactions and the higher docking score compared with those of PMT (Figure 2D). The results gave a possible explanation as to why **1c** was more potent and suggested that **1c** might act on *H. pylori* urease for mediating its anti-*H. pylori* activity. As appropriate substituents might enhance the interactions between PMT derivatives and urease, our docking data provided powerful information for further structural modifications of this kind.

### 2.5. The Inhibitory Effects on Urease of Compound ***1c***

**Figure 3:**
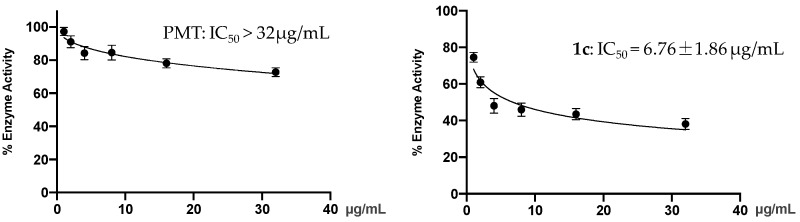
Effects of Palmatine (PMT) and **1c** on the activity of Jack bean urease.

Considering that Jack bean urease shares a high sequence similarity and highly conserved catalytic mechanism with the *H. pylori* urease [32], the inhibitory effects of PMT and **1c** at different concentrations on Jack bean urease were carried out to further validate the docking results. As shown in Figure 3, compound **1c** could dose-dependently inhibit the activity of Jack bean urease, and the half maximal inhibitory concentration (IC_50_) was 6.76 ± 1.86 μg/mL, much lower than that of PMT (>32 μg/mL), as anticipated. These data were in accordance with the phenotype screening and docking results. Therefore, *H. pylori* urease might be one of the targets of PMT derivatives. On the basis of these results, compound **1c** might possess a different mechanism from the first-line anti-*H. pylori* drugs, which provided a possible explanation for the efficacy of compound **1c** against MTZ-resistant *H. pylori* strains.

## 3. Experimental Section

### 3.1. Apparatus, Materials, and Analysis Reagents

Melting point (mp) was obtained with MPA100 OptiMelt automated melting point system (Stanford Research Systems, Palo Alto, CA, USA) and uncorrected. The ^1^H-NMR and ^13^C-NMR spectra were performed on Bruker Avance 600 MHz spectrometer (AV600-III, Bruker, Zürich, Swiss) with Me_4_Si as the internal standard, and all samples were dissolved in dimethyl sulfoxide (DMSO)-*d*_6_ or CD_3_OD and CDCl_3_ before testing. High resolution mass spectra (HRMS)-electronic spray ion (ESI) data were recorded on an Autospec UItima-TOF mass spectrometer (Micromass UK Ltd., Manchester, UK). Flash chromatography was performed on CombiflashRf 200 (Teledyne, Lincoln, NE, USA), with a particle size of 0.038 mm (Figure S1–S20).

PMT was purchased from Xi’an Tianbao Biotechnology Co., Ltd. (Shanxi, China) with purity over 95%. All of the other chemical reagents and anhydrous solvents were purchased from commercial sources (J&K Scientific, Beijing, China) and used without further purification. *H. pylori* ATCC43504 is a standard strain purchased from American Type Culture Collection (ATCC), and other *H. pylori* strains obtained from CAMS Collection Center of Pathogen Microorganisms (CCPM) were isolated from gastric antrum in Beijing, China. MTZ and vancomycin were purchased from National Institutes for Food and Drug Control, Beijing, China. Trimethroprim, polymyxin B sulfate, amphotericin B, and cefsulodin sodium salt were purchased from Sangon Biotech Co., Ltd., Shanghai, China. Mueller–Hinton (MH) agar used for bacterial culture and antimicrobial susceptibility tests was purchased from Becton, Dickinson, and Company (Franklin Lakes, NJ, USA).

### 3.2. General Synthesis Procedures for Compounds ***1a–i*** and ***2***

PMT (387 mg, 1.0 mmol) and corresponding amines (15.0 mmol) were heated for 4–72 h at 95–120 °C. (Table S1) Afterwards, the mixture was cooled and washed with ethyl acetate (15 mL) to remove the remaining amine. The residue was purified using flash column chromatography on silica gel with CH_2_Cl_2_/MeOH as gradient eluent to obtain the desired compounds **1a**–**i** and **2** (Figure S1–S20).

*2,3,10-Trimethoxy-9-benzylaminoprotopalmatine chloride* (**1a**): red solid; yield: 41%; m.p.: 236–238 °C; ^1^H-NMR (DMSO-*d_6_*) *δ* 10.49 (s, 1H), 8.79 (s, 1H), 7.84 (d, *J* = 9.0 Hz, 1H), 7.67 (s, 1H), 7.49 (d, *J* = 8.4 Hz, 1H), 7.43–7.37 (m, 3H), 7.28 (t, *J* = 7.8 Hz, 2H), 7.22–7.15 (tt, *J* = 7.2, 1.2 Hz, 1H), 7.07 (s, 1H), 4.83 (t, *J* = 6.6 Hz, 2H), 4.79 (d, *J* = 6.6 Hz, 2H), 3.93 (s, 3H), 3.86 (s, 3H), 3.85 (s, 3H), 3.23 (t, *J* = 6.6 Hz, 2H); ^13^C-NMR *δ* 151.7, 149.1, 147.0, 146.8, 141.1, 136.8, 136.4, 133.6, 128.7 (3), 127.9 (2), 127.2, 125.4, 119.8, 119.5, 117.7, 116.7, 111.8, 109.1, 57.2, 56.6, 56.3, 55.4, 50.2, 26.7; HRMS: calcd for C_27_H_27_N_2_O_3_Cl [M–Cl]^+^: 427.2016, found: 427.2012.

*2,3,10-Trimethoxy-9-m-methylbenzylaminoprotopalmatinechloride* (**1b**): red solid; yield: 43%; m.p.: 223–225 °C; ^1^H-NMR (DMSO-*d_6_*) *δ* 10.33 (s, 1H), 8.79 (s, 1H), 7.86 (d, *J* = 8.4 Hz, 1H), 7.67 (s, 1H), 7.50 (d, *J* = 8.4 Hz, 1H), 7.25–7.14 (m, 4H), 7.07 (s, 1H), 7.01 (d, *J* = 6.6 Hz, 1H), 4.81 (t, *J* = 6.6 Hz, 2H), 4.75 (d, *J* = 6.6 Hz, 2H), 3.92 (s, 3H), 3.86 (s, 6H), 3.22 (t, *J* = 6.6 Hz, 2H), 2.26 (s, 3H); ^13^C-NMR *δ* 151.2, 148.7, 146.6, 146.3, 140.5, 137.2, 136.4, 135.9, 133.1, 128.2, 128.1, 128.1, 127.4, 124.8, 124.5, 119.3, 119.0, 117.1, 116.3, 111.3, 108.5, 56.8, 56.1, 55.8, 55.0, 49.9, 26.3, 21.1; HRMS: calcd for C_28_H_29_N_2_O_3_Cl [M–Cl]^+^: 441.2173, found: 441.2170.

*2,3,10-Trimethoxy-9-p-methylbenzylaminoprotopalmatinechloride* (**1c**): red solid; yield: 48%; m.p.: 234–236 °C; ^1^H-NMR (DMSO-*d_6_*) *δ* 10.42 (s, 1H), 8.78 (s, 1H), 7.83 (d, *J* = 8.4 Hz, 1H), 7.66 (s, 1H), 7.48 (d, *J* = 8.4 Hz, 1H), 7.32 (t, *J* = 6.6 Hz, 1H), 7.28 (d, *J* = 7.8 Hz, 2H), 7.11–6.99 (m, 3H), 4.82 (t, *J* = 6.6 Hz, 2H), 4.78–4.69 (m, 2H), 3.92 (s, 3H), 3.86 (s, 6H), 3.22 (t, *J* = 6.6 Hz, 2H), 2.22 (s, 3H); ^13^C-NMR *δ* 151.1, 148.6, 146.5, 146.3, 137.5, 136.3, 135.8, 135.8, 133.1, 128.8 (2), 128.2, 127.5 (2), 124.8, 119.3, 119.0, 117.2, 116.2, 111.3, 108.5, 56.7, 56.1, 55.8, 54.9, 49.5, 26.3, 20.7; HRMS: calcd for C_28_H_29_N_2_O_3_Cl [M–Cl]^+^: 441.2173, found: 441.212173.

*2,3,10-Trimethoxy-9-p-methoxybenzylaminoprotopalmatinechloride* (**1d**): red solid; yield: 57%; m.p.: 232–234 °C; ^1^H-NMR (DMSO-*d_6_*) *δ* 10.42 (s, 1H), 8.78 (s, 1H), 7.84 (d, *J* = 8.4 Hz, 1H), 7.66 (s, 1H), 7.49 (d, *J* = 8.4 Hz, 1H), 7.35–7.30 (m, 2H), 7.29–7.20 (m, 1H), 7.07 (s, 1H), 6.86–6.80 (m, 2H), 4.83 (t, *J* = 6.6 Hz, 2H), 4.71 (d, *J* = 6.50 Hz, 2H), 3.92 (s, 3H), 3.89 (s, 3H), 3.86 (s, 3H), 3.68 (s, 3H), 3.22 (t, *J* = 6.6 Hz, 2H); ^13^C-NMR *δ* 158.6, 151.7, 149.2, 147.1, 146.8, 136.8, 136.3, 133.6, 132.9, 129.3 (2), 128.7, 125.2, 119.8, 119.5, 117.7, 116.7, 114.1 (2), 111.8, 109.1, 57.2, 56.6, 56.30, 55.4 (2), 49.8, 26.8; HRMS: calcd for C_28_H_29_N_2_O_4_Cl [M–Cl]^+^: 457.2122, found: 457.2112.

*2,3,10-Trimethoxy-9-m-cyanobenzylaminoprotopalmatinechloride* (**1e**): red solid; yield: 35%; m.p.: 246–248 °C; ^1^H-NMR (DMSO-*d_6_*) *δ* 10.41 (s, 1H), 8.80 (s, 1H), 7.85 (d, *J* = 8.4 Hz, 1H), 7.76 (d, *J* = 8.4 Hz, 2H), 7.68 (s, 1H), 7.59 (d, *J* = 8.4 Hz, 2H), 7.52 (d, *J* = 9.0 Hz, 1H), 7.48 (t, *J* = 7.2 Hz, 1H), 7.09 (s, 1H), 4.89–4.70 (m, 4H), 3.93 (s, 3H), 3.87 (s, 3H), 3.79 (s, 3H), 3.24 (t, *J* = 6.6 Hz, 2H); ^13^C-NMR *δ* 151.7, 149.2, 147.3, 146.9, 146.6, 136.6, 136.0, 133.8, 132.6 (2), 128.7, 128.6 (2), 125.7, 119.9, 119.5, 119.4, 117.9, 117.2, 111.8, 109.9, 109.1, 57.2, 56.6, 56.3, 55.6, 49.9, 26.7; HRMS: calcd for C_28_H_26_N_3_O_3_Cl [M–Cl]^+^: 452.1969, found: 452.1973.

*2,3,10-trimethoxy-9-p-fluorobenzylaminoprotopalmatinechloride* (**1f**): red solid; yield: 54%; m.p.: 226–228 °C; ^1^H-NMR (DMSO-*d_6_*) *δ* 10.44 (s, 1H), 8.79 (s, 1H), 7.85 (d, *J* = 9.0 Hz, 1H), 7.67 (s, 1H), 7.50 (d, *J* = 9.0 Hz, 1H), 7.47–7.40 (m, 2H), 7.36 (t, *J* = 6.6 Hz, 1H), 7.14–7.03 (m, 3H), 4.82 (t, *J* = 6.6 Hz, 2H), 4.75 (d, *J* = 6.0 Hz, 2H), 3.92 (s, 3H), 3.86 (s, 6H), 3.23 (t, *J* = 6.6 Hz, 2H); ^13^C-NMR *δ* 161.1, 151.2, 148.7, 146.6, 146.3, 136.8, 136.8, 136.0 (2), 133.2, 129.4 (2), 128.2, 124.9, 119.3, 119.0, 117.4, 116.5, 114.9 (2), 111.3, 108.5, 56.7, 56.1, 55.8, 55.0, 49.0, 26.3; HRMS: calcd for C_27_H_26_N_2_O_3_FCl [M–Cl]^+^: 445.1922, found: 445.1922.

*2,3,10-Trimethoxy-9-p-bromobenzylaminoprotopalmatinechloride* (**1g**): red solid; yield: 44%; m.p.: 215–217 °C; ^1^H-NMR (DMSO-*d_6_*) *δ* 10.41–10.20 (s, 1H), 8.79 (s, 1H), 7.86 (d, *J* = 8.4 Hz, 1H), 7.67 (s, 1H), 7.51 (d, *J* = 8.4 Hz, 1H), 7.49–7.43 (m, 2H), 7.35 (d, *J* = 8.4 Hz, 2H), 7.29 (d, *J* = 6.6 Hz, 1H), 7.08 (s, 1H), 4.81 (t, *J* = 6.6 Hz, 2H), 4.74 (d, *J* = 7.2 Hz, 2H), 3.92 (s, 3H), 3.86 (s, 3H), 3.85 (s, 3H), 3.23 (t, *J* = 6.6 Hz, 2H); ^13^C-NMR *δ* 151.2, 148.7, 146.6, 146.2, 140.1, 136.0, 135.8, 133.2, 131.1 (2), 129.7 (2), 128.2, 125.0, 119.8, 119.4, 119.0, 117.4, 116.6, 111.3, 108.5, 56.7, 56.1, 55.8, 55.1, 49.2, 26.3; HRMS: calcd for C_27_H_26_N_2_O_3_BrCl [M–Cl]^+^: 505.1121, found: 505.1121.

*2,3,10-Trimethoxy-9-(1-(furan-2-yl)methylamino)protopalmatinechloride* (**1h**): red solid; yield: 19%; m.p.: 231–233 °C; ^1^H-NMR (DMSO-*d_6_*) *δ* 10.21 (s, 1H), 8.84 (s, 1H), 7.93 (d, *J* = 8.4 Hz, 1H), 7.68 (s, 1H), 7.60 (d, *J* = 8.4 Hz, 1H), 7.52 (dd, *J* = 1.8, 0.6 Hz, 1H), 7.08 (s, 1H), 6.87 (t, *J* = 6.6 Hz, 1H), 6.33 (dd, *J* = 3.0, 1.8 Hz, 1H), 6.29–6.12 (m, 1H), 4.83 (t, *J* = 6.6 Hz, 2H), 4.75 (d, *J* = 6.6 Hz, 2H), 3.94 (s, 3H), 3.93 (s, 3H), 3.87 (s, 3H), 3.23 (t, *J* = 6.4 Hz, 2H); ^13^C-NMR *δ* 154.0, 151.7, 149.2, 147.9, 146.6, 142.6, 136.6, 136.0, 133.7, 128.7, 125.5, 120.0, 119.5, 118.3, 118.0, 111.8, 110.8, 109.1, 107.6, 57.3, 56.6, 56.3, 55.6, 43.9, 26.7; HRMS: calcd for C_25_H_25_N_2_O_4_Cl [M–Cl]^+^: 417.1809, found: 417.1811.

*2,3,10-Trimethoxy-9-(1-(pyridyl-2-yl)methylamino)protopalmatinechloride* (**1i**): red solid; yield: 35%; m.p.: 199–201 °C; ^1^H-NMR (DMSO-*d_6_*) *δ* 10.29 (s, 1H), 8.81 (s, 1H), 8.56 (ddd, *J* = 5.4, 1.2, 0.6 Hz, 1H), 7.88 (d, *J* = 9.0 Hz, 1H), 7.78 (td, *J* = 7.8, 1.8 Hz, 1H), 7.69 (s, 1H), 7.53 (d, *J* = 8.4 Hz, 1H), 7.46 (d, *J* = 7.8 Hz, 1H), 7.37–7.24 (m, 2H), 7.09 (s, 1H), 4.94 (d, *J* = 4.8 Hz, 2H), 4.85 (t, *J* = 6.0 Hz, 2H), 3.93 (s, 3H), 3.87 (s, 3H), 3.84 (s, 3H), 3.24 (t, *J* = 6.6 Hz, 2H); ^13^C-NMR *δ* 158.9, 151.1, 148.6, 146.6, 146.3, 136.8, 136.6, 135.7, 133.0, 128.1, 124.2, 122.2, 121.6, 121.5, 119.3, 118.9, 116.6, 116.2, 111.3, 108.5, 56.9, 56.1, 55.7, 55.0, 51.6, 26.2; HRMS: calcd for C_26_H_26_N_3_O_3_Cl [M–Cl]^+^: 428.1969, found: 428.1968.

*2,3,10-Trimethoxy-9-o,p-dimethoxybenzylaminoprotopalmatinechloride* (**2**): red solid; yield: 52%; m.p.: 176–178 °C; ^1^H-NMR (DMSO-*d_6_*) *δ* 10.02 (s, 1H), 8.82 (s, 1H), 7.88 (d, *J* = 9.0 Hz, 1H), 7.68 (s, 1H), 7.55 (d, *J* = 9.0 Hz, 1H), 7.14 (d, *J* = 8.4 Hz, 1H), 7.09 (s, 1H), 6.52 (d, *J* = 1.8 Hz, 1H), 6.45 (s, 1H), 6.40 (dd, *J* = 8.4, 1.8 Hz, 1H), 4.83 (t, *J* = 6.0 Hz, 2H), 4.66 (d, *J* = 3.0 Hz, 2H), 3.93 (s, 3H), 3.87 (s, 3H), 3.86 (s, 3H), 3.77 (s, 3H), 3.71 (s, 3H), 3.23 (t, *J* = 6.0 Hz, 2H); ^13^C-NMR *δ* 160.3, 158.3, 151.6, 149.1, 147.7, 146.8, 137.2, 136.3, 133.5, 130.0, 128.6, 124.8, 120.2, 119.9, 119.5, 117.9, 117.4, 111.8, 109.0, 104.6, 98.7, 57.3, 56.6, 56.3, 55.8, 55.6 (2), 46.8, 26.7; HRMS: calcd for C_29_H_31_N_2_O_5_Cl [M–Cl]^+^: 487.2227, found: 487.2227.

### 3.3. Synthesis of ***3***

*2,3,10-Trimethoxy-9-aminoprotopalmatine chloride* (**3**): The mixture of compound **2** (522 mg, 1.00 mmol) and methanol/HCl (1:1 by vol., 20 mL) was reacted at room temperature for 24 h (Table S1). Then, the solution was stopped and concentrated, and the residue was further purified through flash column chromatography on silica gel with CH_3_OH/CH_2_Cl_2_ as the eluents to get the desired compound **3** as a red solid, yield: 73%; m.p.: 266–268 °C; ^1^H-NMR (DMSO-*d_6_*) *δ* 10.35 (s, 1H), 8.72 (s, 1H), 7.84 (d, *J* = 8.4 Hz, 1H), 7.66 (s, 1H), 7.34 (d, *J* = 9.0 Hz, 1H), 7.07 (s, 1H), 6.90 (s, 2H), 4.74 (t, *J* = 6.0 Hz, 2H), 3.98 (s, 3H), 3.93 (s, 3H), 3.86 (s, 3H), 3.22 (t, *J* = 6.0 Hz, 2H); ^13^C-NMR *δ* 151.4, 149.1, 147.0, 143.7, 138.0, 135.6, 132.3, 128.4, 123.1, 119.7, 119.5, 113.8, 113.2, 111.8, 108.9, 56.9, 56.6, 56.3, 55.3, 26.8; HRMS: calcd for C_20_H_21_N_2_O_3_Cl [M–Cl]^+^: 337.1547, found: 337.1547.

### 3.4. General Procedure for the Synthesis of Compounds ***4a–i***

To a stirred solution of compound **3** (100 mg, 0.27 mmol) in anhydrous CH_3_CN (6 mL), pyridine (1.22 mmol) and corresponding acyl chloride (0.81 mmol) were added. The reaction mixture was heated at 71 °C for 3–24 h (Table S1). The mixture was cooled and filtered, and the resulting residue was washed with CH_2_Cl_2_ and 80% EtOH to acquired desired compounds **4a**–**i** (Figure S1–S20).

*2,3,10-Trimethoxy-9-benzoylaminoprotopalmatine chloride* (**4a**): yellow solid; yield: 37%; m.p.: 215–217 °C; ^1^H-NMR (DMSO-*d_6_*) *δ* 10.47 (s, 1H), 9.76 (s, 1H), 9.14 (s, 1H), 8.28 (q, *J* = 9.0 Hz, 2H), 8.17–8.12 (m, 2H), 7.76 (s, 1H), 7.70–7.64 (m, 1H), 7.60 (t, *J* = 7.2 Hz, 2H), 7.10 (s, 1H), 4.98 (t, *J* = 6.6 Hz, 2H), 4.03 (s, 3H), 3.95 (s, 3H), 3.87 (s, 3H), 3.22 (t, *J* = 6.6 Hz, 2H); ^13^C-NMR *δ* 175.7, 164.3, 161.0, 158.2, 155.5, 147.3, 143.0, 142.8, 141.5, 138.2, 137.9, 137.7 (2), 137.2 (2), 134.8, 133.9, 131.3, 130.0, 128.4, 120.8, 118.3, 66.4, 65.7, 65.4, 64.8, 35.4; HRMS: calcd for C_27_H_25_N_2_O_4_Cl [M–Cl]^+^: 441.1809, found: 441.1812.

*2,3,10-Trimethoxy-9-p-tert-butylbenzoylaminoprotopalmatine chloride* (**4b**): yellow solid; yield: 45%; m.p.: 210–212 °C; ^1^H-NMR (DMSO-*d_6_*) *δ* 10.36 (s, 1H), 9.73 (s, 1H), 9.13 (s, 1H), 8.39–8.21 (m, 2H), 8.15–7.99 (m, 2H), 7.75 (s, 1H), 7.67–7.53 (m, 2H), 7.10 (s, 1H), 4.97 (t, *J* = 6.6 Hz, 2H), 4.03 (s, 3H), 3.96 (s, 3H), 3.87 (s, 3H), 3.21 (t, *J* = 6.6 Hz, 2H), 1.36 (s, 9H); ^13^C-NMR *δ* 166.0, 155.0, 154.8, 151.5, 148.7, 146.0, 137.8, 133.3, 130.8, 128.7, 128.1 (2), 127.6, 125.3, 125.2 (2), 124.5, 122.0, 120.5, 118.9, 111.3, 108.8, 56.9, 56.2, 55.9, 55.3, 34.8, 31.0 (3), 25.9; HRMS: calcd for C_31_H_33_N_2_O_4_Cl [M–Cl]^+^: 497.2435, found: 497.2434.

*2,3,10-Trimethoxy-9-p-fluorobenzoylaminoprotopalmatine chloride* (**4c**): yellow solid; yield: 49%; m.p.: 239–241 °C; ^1^H-NMR (DMSO-*d*_6_): *δ* 10.63 (s, 1H), 9.80 (s, 1H), 9.15 (s, 1H), 8.42–8.19 (m, 2H), 8.07–7.86 (m, 2H), 7.76 (s, 1H), 7.69–7.63 (m, 1H), 7.54 (td, *J* = 9.0, 2.4 Hz, 1H), 7.10 (s, 1H), 4.98 (t, *J* = 6.6 Hz, 2H), 4.03 (s, 3H), 3.96 (s, 3H), 3.87 (s, 3H), 3.22 (t, *J* = 6.6 Hz, 2H); ^13^C-NMR *δ* 164.9, 162.8, 161.2, 154.8, 151.5, 148.7, 145.9, 137.8, 135.9, 133.4, 130.6, 130.6, 128.7, 127.9, 125.3, 124.4, 124.3, 121.35, 120.48, 118.9, 115.1, 115.0, 111.3, 108.9, 57.0, 56.2, 55.9, 55.3, 25.9; HRMS: calcd for C_27_H_24_N_2_O_4_FCl [M–Cl]^+^: 459.1715, found: 459.1711.

*2,3,10-Trimethoxy-9-(2′-furoylamino)protopalmatine chloride* (**4d**): yellow solid; yield: 37%; m.p.: 244–246 °C; ^1^H-NMR (CD_3_OD, CDCl_3_) *δ* 9.44 (s, 1H), 8.75 (s, 1H), 8.24 (d, *J* = 9.0 Hz, 1H), 8.06 (d, *J* = 9.0 Hz, 1H), 7.73 (dd, *J* = 2.4, 1.2 Hz, 1H), 7.56 (s, 1H), 7.37 (dd, *J* = 3.6, 0.6 Hz, 1H), 6.93 (s, 1H), 6.66 (dd, *J* = 3.6, 1.8 Hz, 1H), 4.88 (t, *J* = 6.6 Hz, 2H), 4.10 (s, 3H), 4.02 (s, 3H), 3.95 (s, 3H), 3.25 (t, *J* = 6.6 Hz, 2H); ^13^C-NMR *δ* 159.4, 155.6, 153.2, 150.3, 147.4, 146.6, 146.4, 139.1, 134.8, 129.0 (2), 125.4, 124.9, 121.5, 121.0, 119.5, 117.3, 113.2, 111.6, 109.1, 57.4, 57.1, 56.9, 56.6, 27.5; HRMS: calcd for C_25_H_23_N_2_O_5_Cl [M–Cl]^+^: 431.1602, found: 431.1604.

*2,3,10-Trimethoxy-9-(2′,5′-dimethyl-3′-furoylamino)protopalmatine chloride* (**4e**): yellow solid; yield: 40%; m.p.: 236–238 °C; ^1^H-NMR (DMSO-*d_6_*) *δ* 9.87 (s, 1H), 9.67 (s, 1H), 9.12 (s, 1H), 8.30–8.18 (m, 2H), 7.75 (s, 1H), 7.10 (s, 1H), 6.83 (s, 1H), 4.99 (t, *J* = 6.6 Hz, 2H), 4.03 (s, 3H), 3.95 (s, 3H), 3.87 (s, 3H), 3.22 (t, *J* = 6.6 Hz, 2H), 2.51 (s, 3H), 2.32 (s, 3H); ^13^C NMR *δ* 162.9, 155.7, 154.7, 151.5, 149.2, 148.7, 146.1, 137.7, 133.3, 128.7, 127.5, 125.2, 124.5, 121.6, 120.4, 118.9, 116.0, 111.3, 108.8, 105.7, 56.9, 56.2, 55.9, 55.4, 26.0, 13.4, 13.1; HRMS: calcd for C_27_H_27_N_2_O_5_Cl [M–Cl]^+^: 459.1915, found: 459.1914.

*2,3,10-Trimethoxy-9-(1′,5′-dimethyl-1′H-pyrazole-3′-ylcarbon*y*lamino)protopalmatine chloride* (**4f**): yellow solid; yield: 42%; m.p.: 192–194 °C; ^1^H-NMR (DMSO-*d_6_*) *δ* 9.92 (s, 1H), 9.65 (s, 1H), 9.12 (s, 1H), 8.30–8.12 (m, 2H), 7.74 (s, 1H), 7.10 (s, 1H), 6.62 (s, 1H), 4.97 (t, *J* = 6.6 Hz, 2H), 4.00 (s, 3H), 3.95 (s, 3H), 3.89 (s, 3H), 3.87 (s, 3H), 3.21 (t, *J* = 6.6 Hz, 2H), 2.36 (s, 3H); ^13^C-NMR *δ* 161.1, 154.6, 151.5, 148.7, 146.2, 143.9, 140.7, 137.5, 133.2, 128.6, 127.4, 125.2, 124.3, 121.6, 120.4, 118.9, 111.3, 108.8, 106.3, 56.9, 56.2, 55.9, 55.4, 36.6, 26.0, 10.8; HRMS: calcd for C_26_H_27_N_4_O_4_Cl [M–Cl]^+^: 459.2027, found: 459.2029.

*2,3,10-Trimethoxy-9-(3′,5′-dimethylisoxazole-4′-ylcarbon*y*lamino)protopalmatine chloride* (**4g**): brown solid; yield: 47%; m.p.: 262–264 °C; ^1^H-NMR (CD_3_OD, CDCl_3_) *δ* 9.61 (s, 1H), 8.85 (s, 1H), 8.32 (d, *J* = 9.0 Hz, 1H), 8.14 (d, *J* = 9.0 Hz, 1H), 7.64 (s, 1H), 7.01 (s, 1H), 4.97 (t, *J* = 6.6 Hz, 2H), 4.16 (s, 3H), 4.06 (s, 3H), 4.00 (s, 3H), 3.32 (d, *J* = 6.6 Hz, 2H), 2.79 (s, 3H), 2.56 (s, 3H); ^13^C-NMR *δ* 173.0, 163.8, 159.9, 155.7, 153.4, 150.5, 146.5, 139.4, 135.0, 129.3 (2), 125.5, 125.0, 121.6, 121.4, 119.7, 113.1, 111.8, 109.4, 57.5, 57.2, 56.9, 56.7, 27.5, 13.0, 11.3; HRMS: calcd for C_26_H_26_N_3_O_5_Cl [M–Cl]^+^: 460.1867, found: 460.1870.

*2,3,10-Trimethoxy-9-(5′-methyl-3′-phenylisoxazole-4′-ylcarbonylamino)protopalmatine chloride* (**4h**): yellow solid; yield: 35%; m.p.: 221–223 °C; ^1^H-NMR (DMSO-*d_6_*) *δ* 10.38 (s, 1H), 9.54 (s, 1H), 9.12 (s, 1H), 8.30 (s, 2H), 7.85 (s, 2H), 7.74 (s, 1H), 7.53 (s, 3H), 7.12 (s, 1H), 4.95 (s, 2H), 4.11 (s, 3H), 3.95 (s, 3H), 3.88 (s, 3H), 3.25 (t, *J* = 6.6 Hz, 2H), 2.81 (s, 3H); ^13^C-NMR *δ* 172.5, 162.3, 161.5, 155.4, 152.7 (2), 149.8 (2), 146.1, 138.8, 134.4, 130.7, 129.3 (3), 129.0, 128.8, 125.6, 124.4, 121.1, 119.5, 112.7, 111.8 (2), 109.4(2), 57.3, 56.7, 56.5, 56.2, 26.7, 12.6; HRMS: calcd for C_31_H_28_N_3_O_5_Cl [M–Cl]^+^: 522.2024, found: 522.2028.

*2,3,10-Trimethoxy-9-nicotinoylaminoprotopalmatine chloride* (**4i**): yellow solid; yield: 36%; m.p.: 248–250 °C; ^1^H-NMR (CD_3_OD, CDCl_3_) *δ* 9.98 (s, 1H), 9.65 (d, *J* = 2.4 Hz, 1H), 9.34–9.28 (m, 1H), 9.09–9.03 (m, 1H), 8.85 (s, 1H), 8.35 (d, *J* = 9.0 Hz, 1H), 8.26 (dd, *J* = 8.4, 6.0 Hz, 1H), 8.14 (d, *J* = 9.0 Hz, 1H), 7.64 (s, 1H), 6.99 (s, 1H), 5.03 (t, *J* = 6.6 Hz, 2H), 4.14 (s, 3H), 4.07 (s, 3H), 4.00 (s, 3H), 3.31 (t, *J* = 6.6 Hz, 2H); ^13^C-NMR *δ* 163.6, 156.1, 153.3, 150.4, 146.4, 145.6 (2), 143.9, 139.5, 135.1, 133.8, 129.7, 129.3, 127.8, 125.6, 125.2, 121.6, 120.6, 119.6, 111.7, 109.3, 57.5, 56.9, 56.9, 56.6, 27.5; HRMS: calcd for C_26_H_24_N_3_O_4_Cl [M–Cl]^+^: 442.1761, found: 442.1767.

### 3.5. Biology Assay

#### 3.5.1. Antimicrobial Assay

Six *H. pylori* strains from CAMS Collection Center of Pathogen Microorganisms (CCPM), including *H. pylori* strain ATCC43504, a standard strain isolated from human gastric antrum in Australia, and five clinical isolated strains from Chinese hospitals, were employed in the antibacterial activities study. MICs of the target compounds against *H. pylori* were performed using the agar dilution method following Clinical and Laboratory Standards Institute (CLSI) guidelines (M45). Briefly, the bacterial suspension adjusted to 2.0 McFarland density (about 10^8^ CFU/mL) was prepared from an MH agar plate (9 cm) containing 5% defibrinated sheep blood and selective antibiotics (e.g., vancomycin, trimethroprim, polymyxin B sulfate, amphotericin B, and cefsulodin sodium salt) [33]. Then, 2.5 μL of the bacterial suspension in triplicate was directly inoculated onto the MH agar plates with 5% defibrinated sheep blood containing 2-fold serial dilutions of test compounds (0.5–256 μg/mL). The plates were incubated at 37 °C for 72 h under microaerobic conditions (10% CO_2_). *H. pylori* ATCC43504 strain was used as a control strain. The MICs were defined as the lowest concentration of drug showing no growth. The MIC results were obtained from two independent experiments. The resistance breakpoint for MTZ was defined as *>*8 μg/mL.

#### 3.5.2. Acute Toxicity

The weights of sixteen experimental female and sixteen male Kunming mice were 20.0 ± 1.0 g. They were purchased from the Institute of Laboratory Animal Science (Beijing, China). According to the institutional guidelines of the Institute of Materia Medica, CAMS&PUMC, all mice were fed with regular rodent chow and housed in an air-conditioned room. The mice were randomly allocated into different groups with four female and four male in each group. The compound **1c** was given orally in a single-dosing experiment at 0, 250, 500, or 1000 mg/kg (saline as control), respectively. The mice were closely monitored for seven days. Body weight as well as survival was monitored. Animal experiments were approved by the Ethics Committee of the Institute of Medicinal Biotechnology, Chinese Academy of Medical Sciences and Peking Union Medical College, Beijing, China, with the approval number of IBM20190510D201 (Approval date: 05/10/2019).

#### 3.5.3. Molecular Docking Assay

Crystal structure of *H. pylori* urease was obtained from the Protein Data Bank (PDB code 1E9Y, resolution: 3 Å) [31]. The binding modes between *H. pylori* urease and **1c** or PMT were generated using the LibDock software within Discovery Studio v4.5 (BIOVIA, San Diego, CA, USA). Before docking, protein structure and ligands were processed by Discovery Studio v4.5, such as ligand energy minimization, protein preparation, and so on [34]. The receptor ligand interaction with the highest docking score in the Discovery Studio v4.5 software was the final result of the docking experiment.

#### 3.5.4. Enzyme Inhibition Assay

Urease (Type III from *Canavalia ensiformis*; Sigma-Aldrich, St. Louis, MO, USA) 0.05 mg/mL and different concentrations of **1c** (1, 2, 4, 8, 16, and 32 μg/mL) or PMT (1, 2, 4, 8, 16, and 32 μg/mL) were incubated at 37 °C in assay buffer in the absence of urea for 20 min. Then, the reactions were initiated by mixing the urea and incubating for 10 min at room temperature [22,35]. The urease activity was determined by Urease Activity Assay Kit (Sigma-Aldrich).

## 4. Conclusions

Taking PMT as the lead, 20 new derivatives were synthesized and examined for their antibacterial activities against different MTZ-resistant *H. pylori* strains. SAR analysis indicated that the introduction of a suitable secondary amine substituent at the 9-position might be beneficial for potency. Among them, compound **1c** exhibited the most potent activities against MTZ-resistant strains with MIC values of 4–16 μg/mL, better than those of PMT and MTZ. Compound **1c** exhibited a good safety character, with an LD_50_ of over 1000 mg/kg by oral administration. Molecular docking and enzyme inhibition assay verified that **1c** might act on *H. pylori* urease for mediating its activity. Therefore, we consider PMT derivatives to be a new family of anti-MTZ-resistant *H. pylori* components.

## Figures and Tables

**Table 1:**
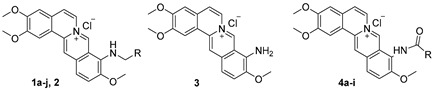
Antimicrobial activities of the target compounds against metronidazole (MTZ)-resistant *H. pylori* strains (minimum inhibitory concentration (MIC), μg/mL).

Code	R	ATCC 43504 ^a^	CCPMAP 160007	CCPMAP 160008	CCPMAP 160010	CCPMAP 160011	CCPMAP 160017
PMT	-	64	64	128	128	128	256
**1a**	C_6_H_5_	32	8	64	16	64	64
**1b**	*m*-CH_3_C_6_H_4_	16	4	8	4	16	16
**1c**	*p*-CH_3_C_6_H_4_	16	4	4	4	8	16
**1d**	*p*-CH_3_OC_6_H_4_	16	4	32	8	16	32
**1e**	*m*-CNC_6_H_4_	>256	128	>256	256	>256	>256
**1f**	*p*-FC_6_H_4_	128	64	128	8	128	128
**1g**	*p*-BrC_6_H_4_	64	8	16	4	64	64
**1h**	*2*′*-*furyl	32	8	64	16	16	16
**1i**	2′-pyridyl	64	16	128	32	64	128
**2**	2′,4′-(CH_3_O)_2_ C_6_H_3_	16	4	32	4	16	16
**3**	-	>128	>128	>128	>128	>128	>128
**4a**	C_6_H_5_	>256	64	>256	>256	>256	>256
**4b**	*p*-(CH_3_)_3_CC_6_H_4_	>256	16	>256	128	>256	>256
**4c**	*m*-FC_6_H_4_	>256	128	>256	256	>256	>256
**4d**	*2*′*-*furyl	256	128	>256	256	>256	>256
**4e**	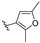	256	128	>256	256	>256	>256
**4f**	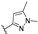	>256	128	>256	256	>256	>256
**4g**	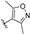	>256	>256	>256	>256	>256	>256
**4h**	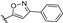	>256	>256	>256	>256	>256	>256
**4i**	*3*′*-*pyridyl	>256	>256	>256	>256	>256	>256
MTZ ^b^	-	128	32	32	16	64	128

^a^*H. pylori* ATCC43504 strain served as MIC quality control (MTZ: 64–256 μg/mL); ^b^ MTZ: ≤8 μg/mL for susceptible and >8 μg/mL for resistant.

## Supplementary Materials

The following are available online, Figures S1–S20: Spectra of the analyzed target compounds **1a**–**i**, **2**, **3**, **4a**–**i**, Table S1: The reaction times and temperatures of each target compounds.
