# Supplementary material for: Synthesis and Structure–Activity Relationship of Palmatine Derivatives as a Novel Class of Antibacterial Agents against Helicobacter pylori
Source: Molecules. 2020 Mar 16;25(6):1352. doi: 10.3390/molecules25061352 (PMC7146163; doi:10.3390/molecules25061352)

Synthesis and Structure-activity Relationship of Palmatine Derivatives as a Novel Class of Antibacterial Agents against *Helicobacter pylori*

Tianyun Fan[1,†], Xixi Guo [1,†], Qingxuan Zeng [1], Wei Wei [1], Xuefu You [1], Jing Pang [1,*], Yanxiang Wang [1,2,*] and Danqing Song [1]

[1] Beijing Key Laboratory of Antimicrobial Agents, Institute of Medicinal Biotechnology, Chinese Academy of Medical Sciences and Peking Union Medical College, Beijing 100050, China;

[2] State Key Laboratory of Bioactive Substance and Function of Natural Medicines, Institute of Materia Medica, Chinese Academy of Medical Sciences and Peking Union Medical College, Beijing 100050, P. R. China

fty1668@163.com (T.F.); sissi.kwok@outlook.com (X.G.); zqx50810793@163.com (Q.Z.); weiwei082695@163.com (W.W.); 13311123098@163.com (X.Y.); songdanqingsdq@hotmail.com (D.S.).

* Correspondence: pangjing.pangjing@163.com (J.P.); wangyanxiang@imb.pumc.edu.cn (Y.W.) Tel.:+86-10-67058991 (J.P.); +86-10-63033012 (Y.W.)

† These authors contributed equally to this work.

Table SI. The reaction time and temperature of each target compound.

Code	reaction time (h)	reaction temperature (°C)	yield (%)
**1a**	6	110	41
**1b**	4	110	43
**1c**	4	110	48
**1d**	6	110	57
**1e**	8	110	35
**1f**	7	110	54
**1g**	7	110	44
**1h**	72	120	19
**1i**	72	95	35
**2**	6	110	52
**3**	24	25	73
**4a**	6	71	37
**4b**	3	71	45
**4c**	6	71	49
**4d**	8	71	37
**4e**	8	71	40
**4f**	24	71	42
**4g**	24	71	47
**4h**	8	71	35
**4i**	24	71	36

Figure S1: ^{1}H NMR, ^{13}C NMR, HRMS-ESI spectra of compound **1a**

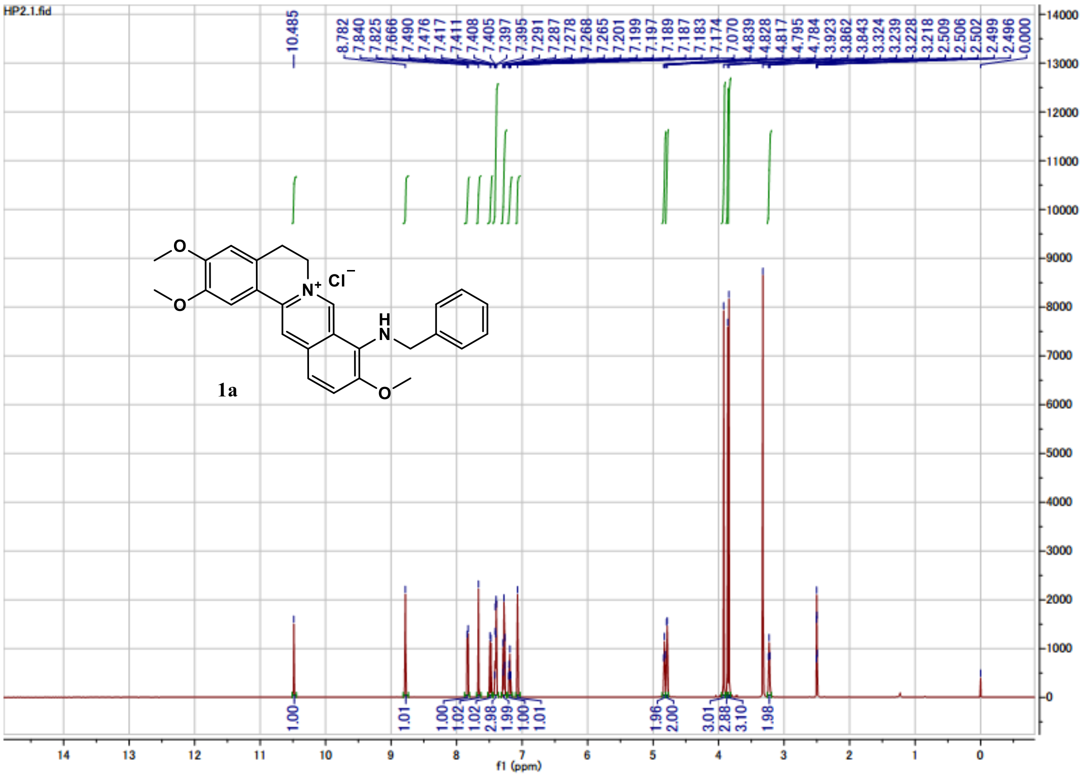

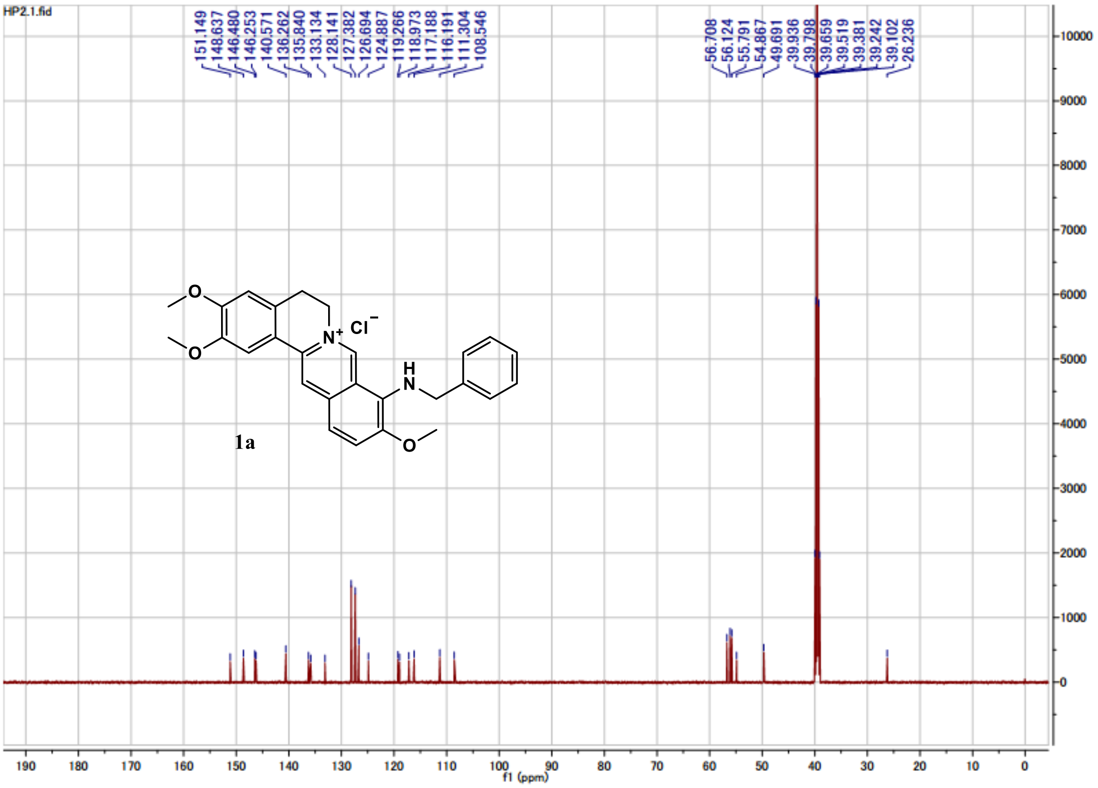

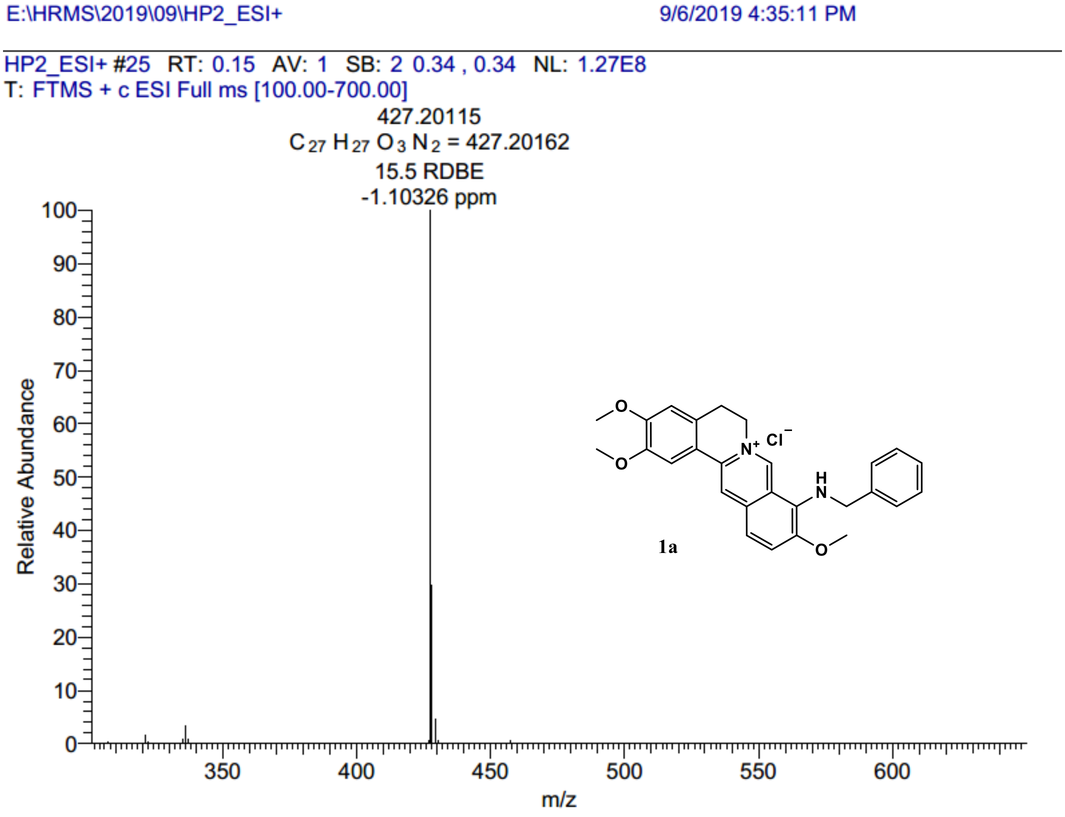

Figure S2: ^{1}H NMR, ^{13}C NMR, HRMS-ESI spectra of compound**1b**

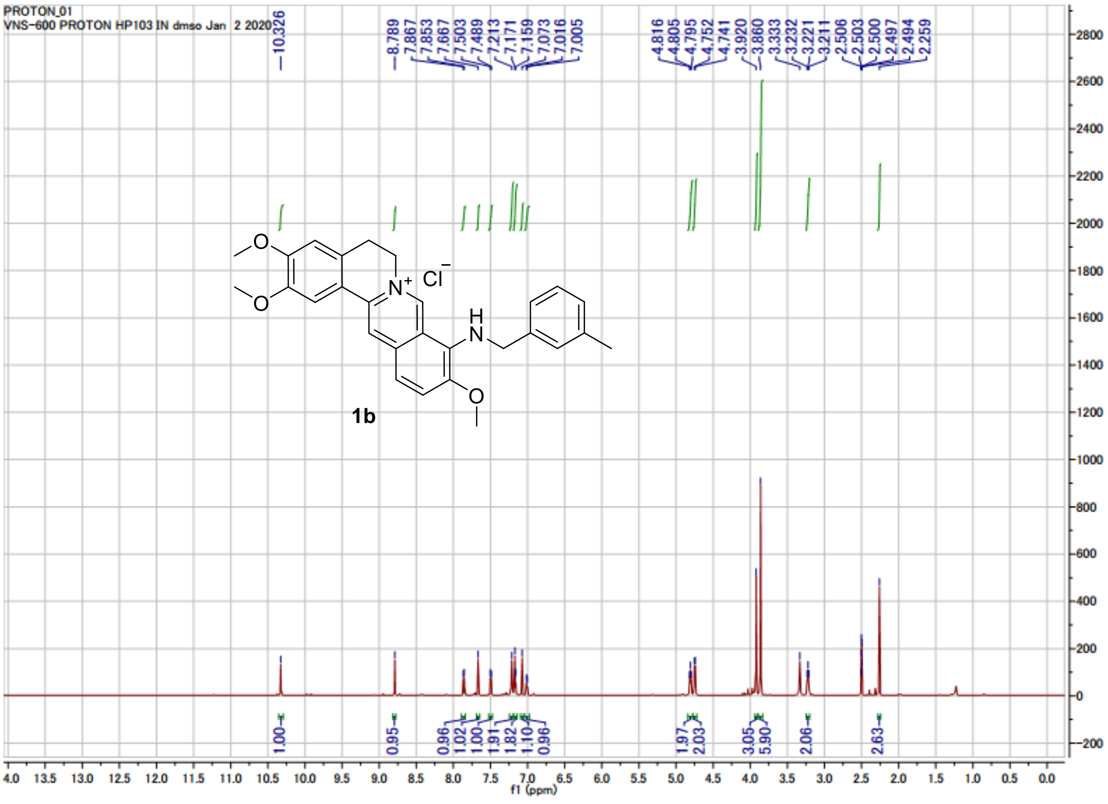

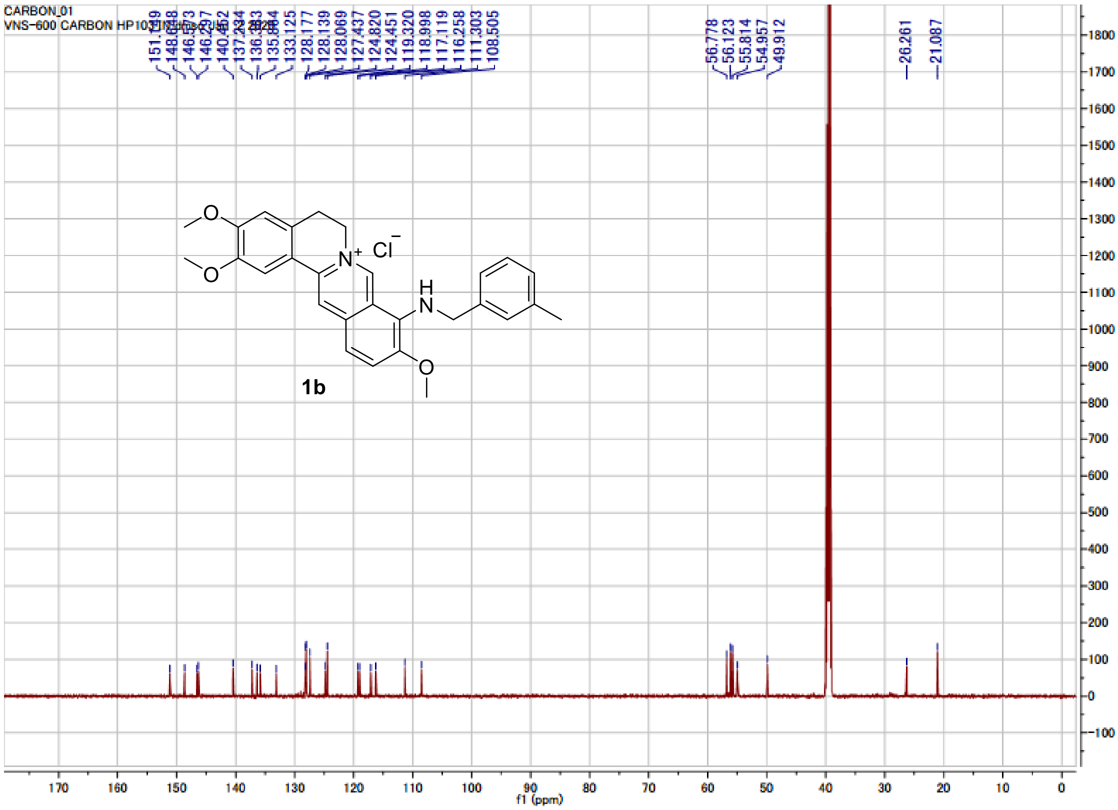

E:\HRMS\...\12\13\FTY\HP103_191213160724 12/13/2019 4:39:18 PM

HP103_191213160724 #30 RT: 0.30 AV: 1 NL: 2.08E7
T: FTMS + c ESI Full ms [100.00-2000.00]

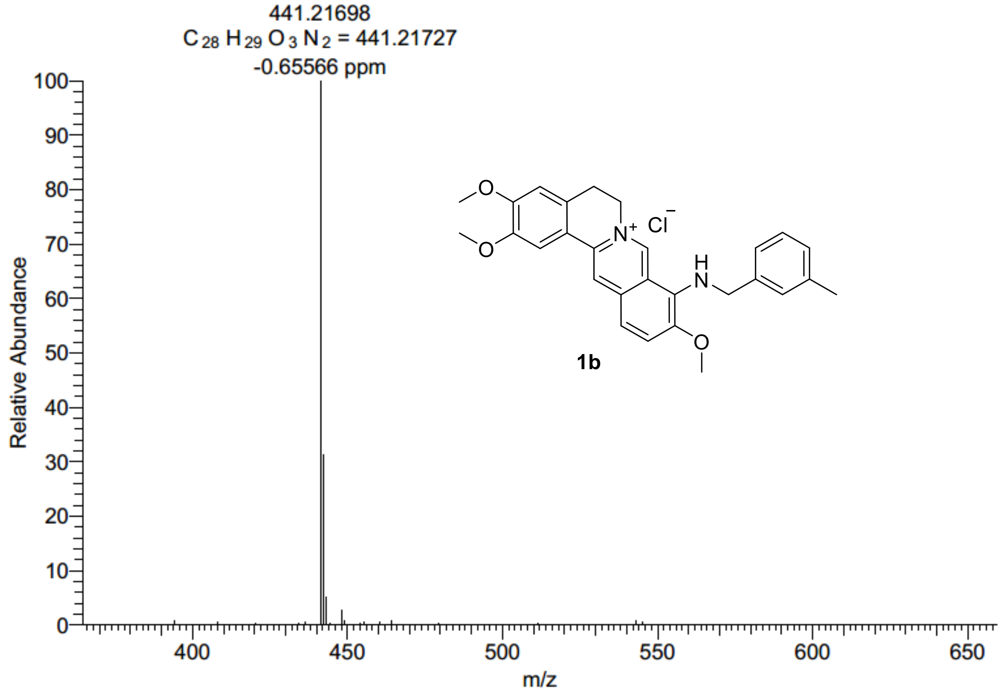

Figure S3: ^{1}H NMR, ^{13}C NMR, HRMS-ESI spectra of compound **1c**

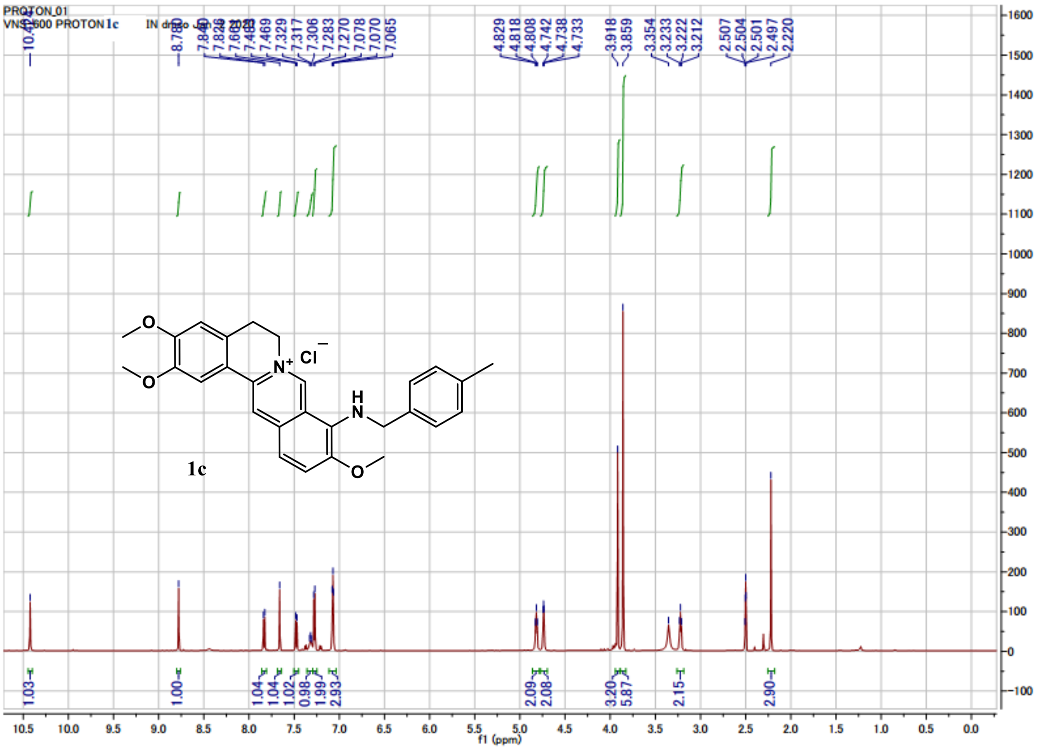

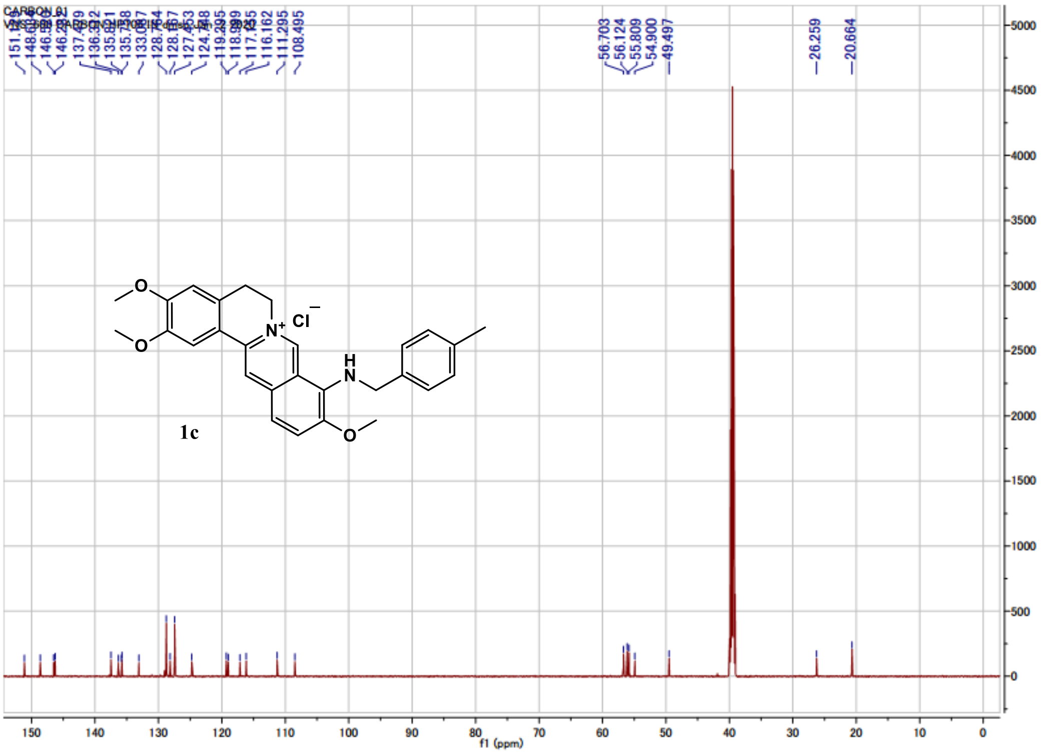

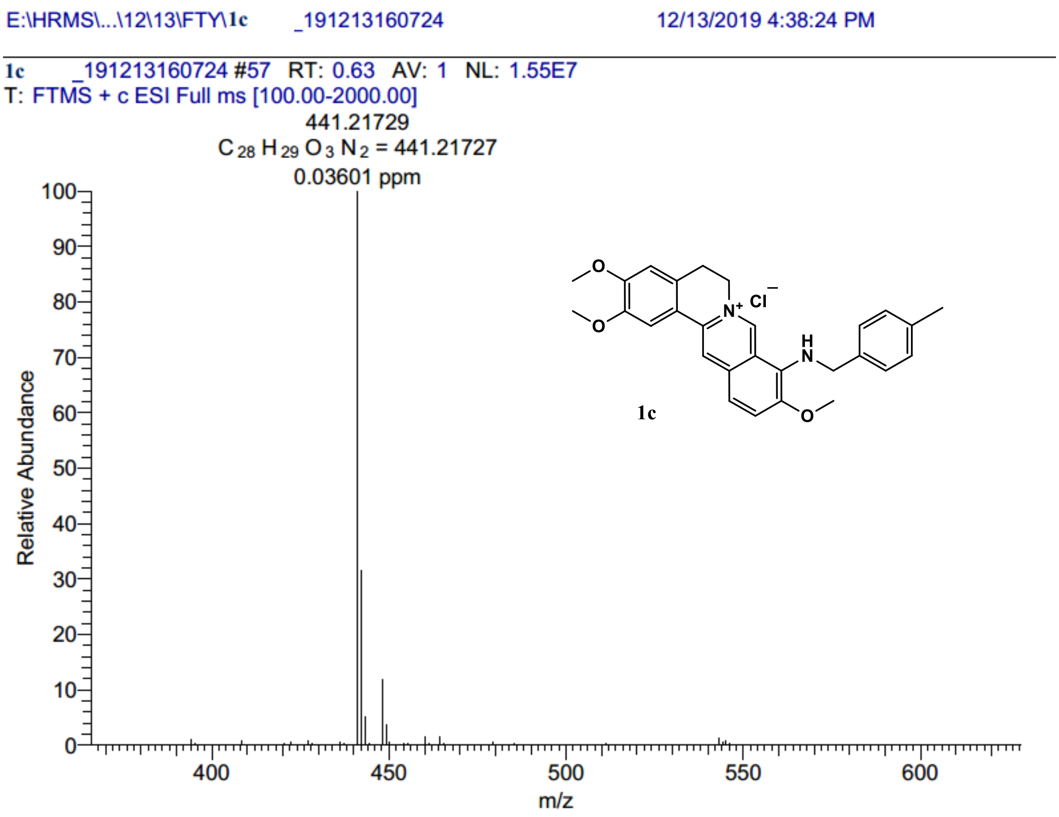

Figure S4: ^{1}H NMR, ^{13}C NMR, HRMS-ESI spectra of compound **1d**

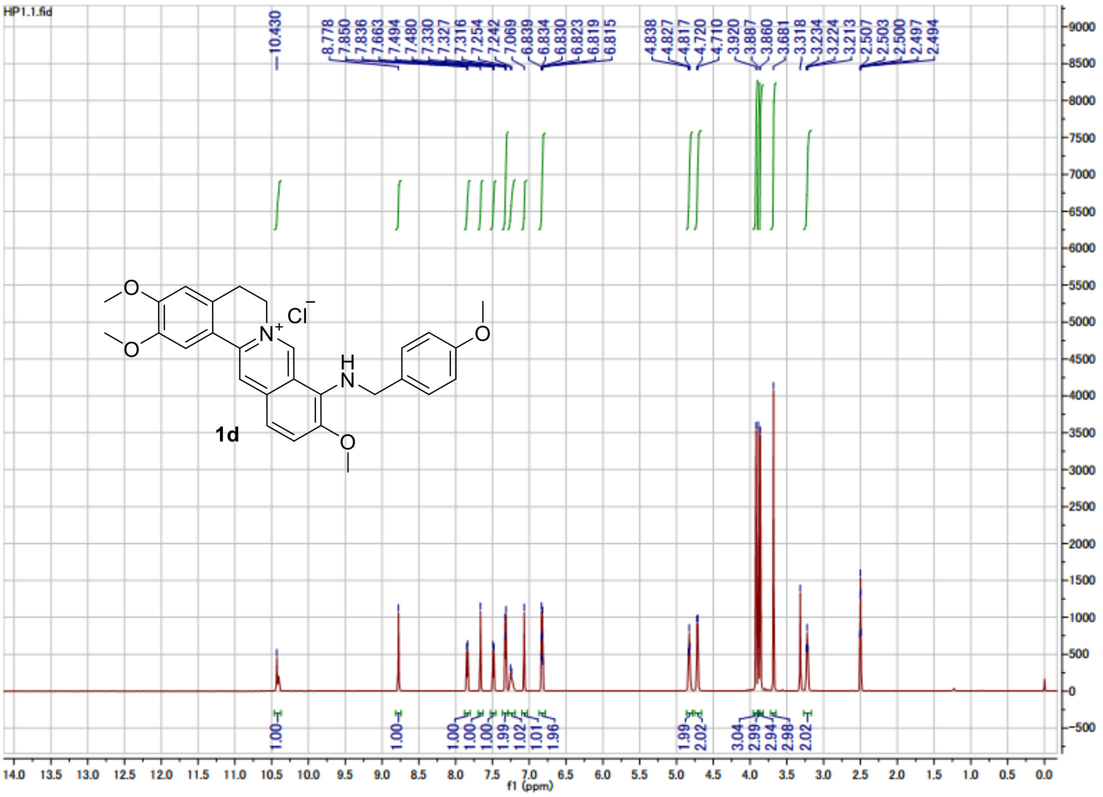

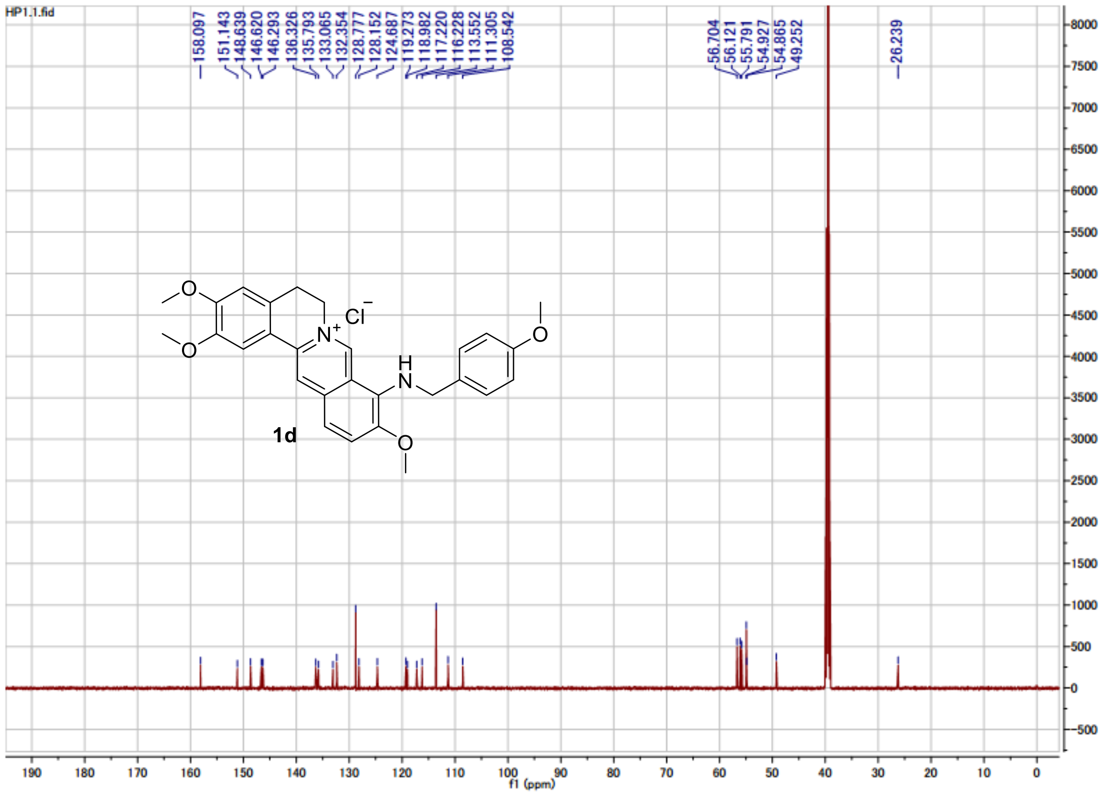

E:\HRMS\2019\09\HP1_ESI+ 9/6/2019 4:34:09 PM

HP1_ESI+ #23 RT: 0.13 AV: 1 SB: 2 0.91 , 0.91 NL: 1.02E8
T: FTMS + c ESI Full ms [100.00-700.00]

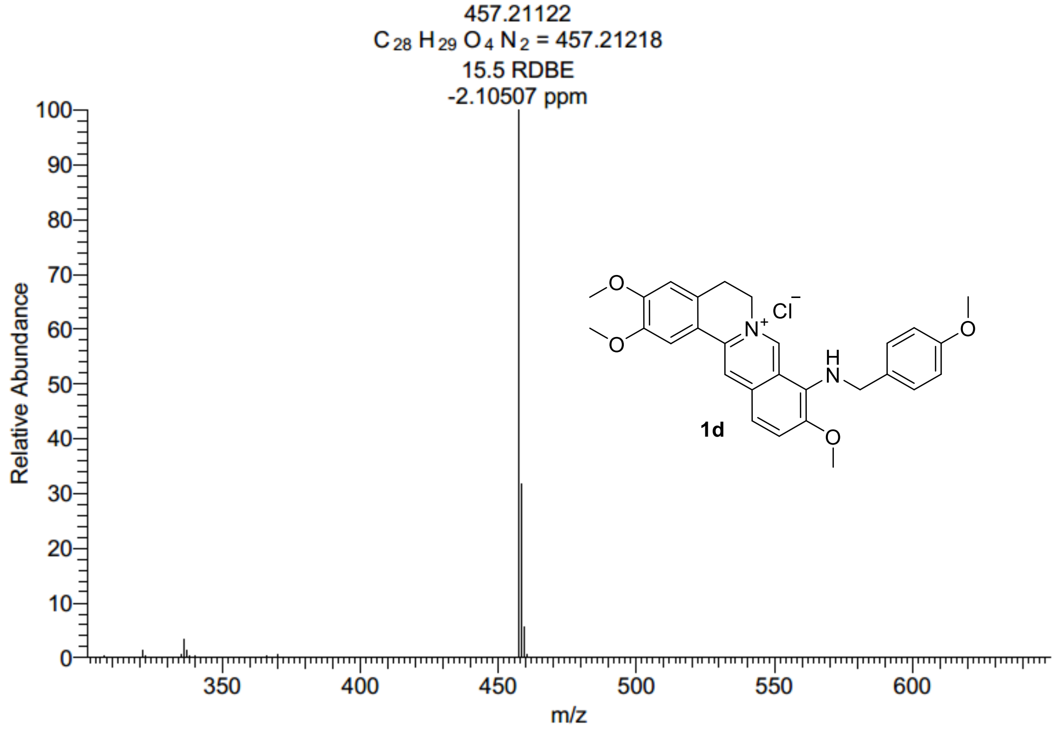

Figure S5: ^{1}H NMR, ^{13}C NMR, HRMS-ESI spectra of compound **1e**

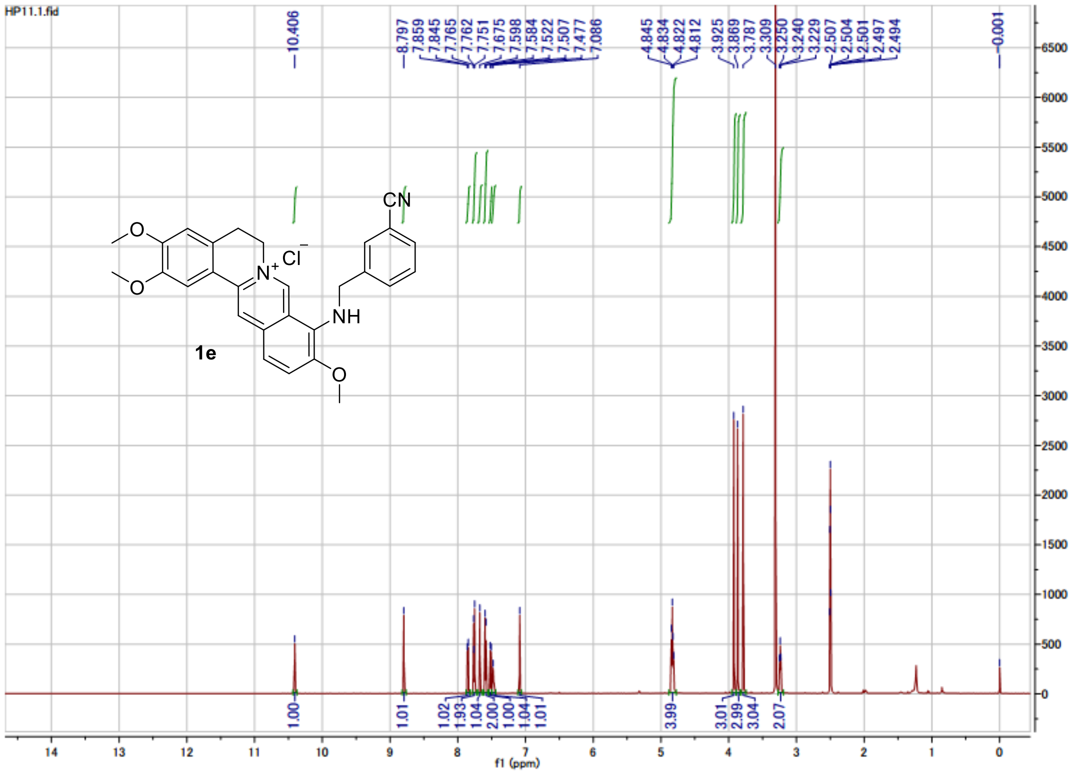

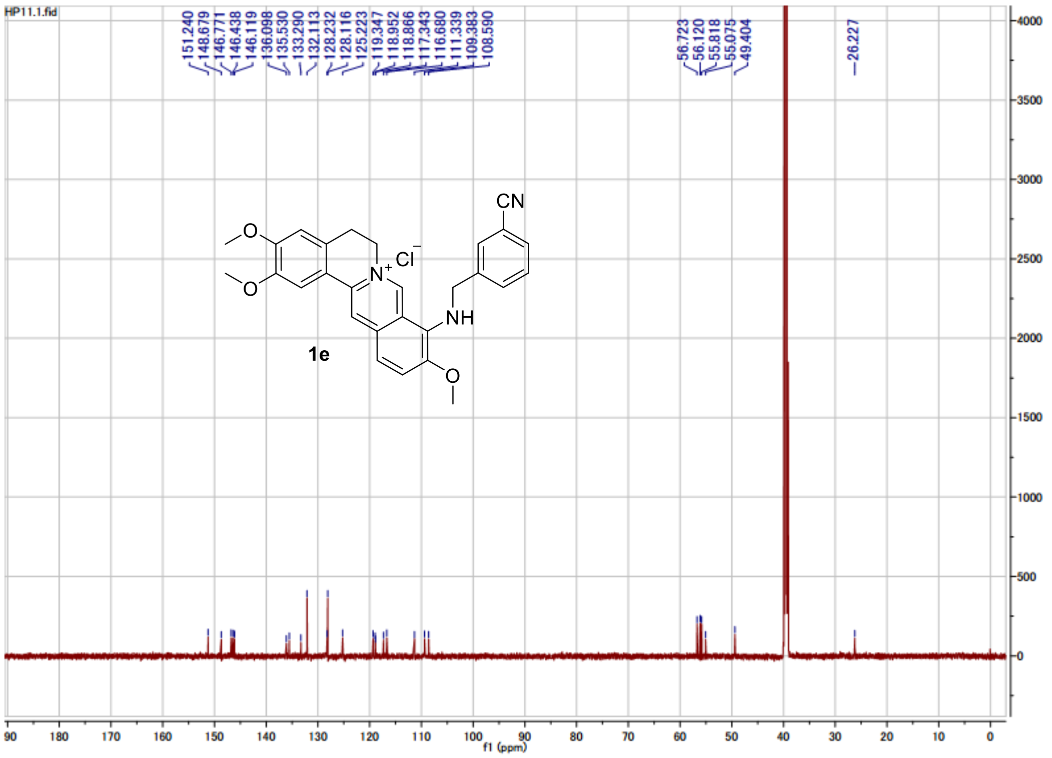

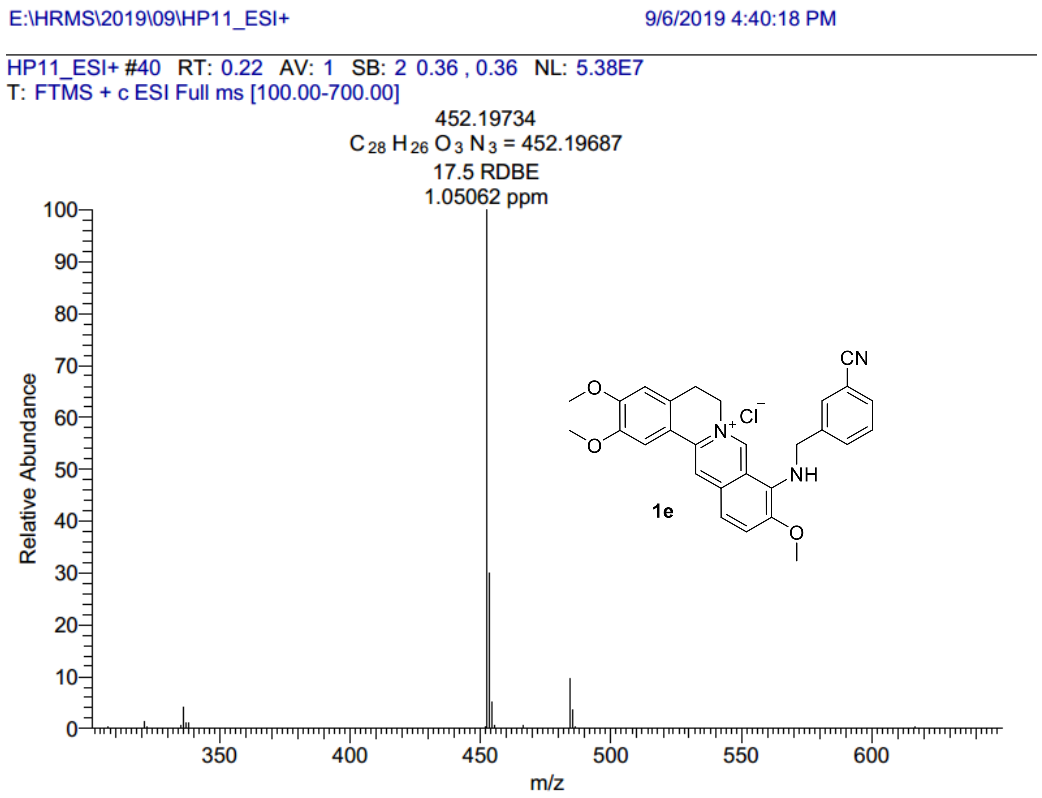

Figure S6: ^{1}H NMR, ^{13}C NMR, HRMS-ESI spectra of compound **1f**

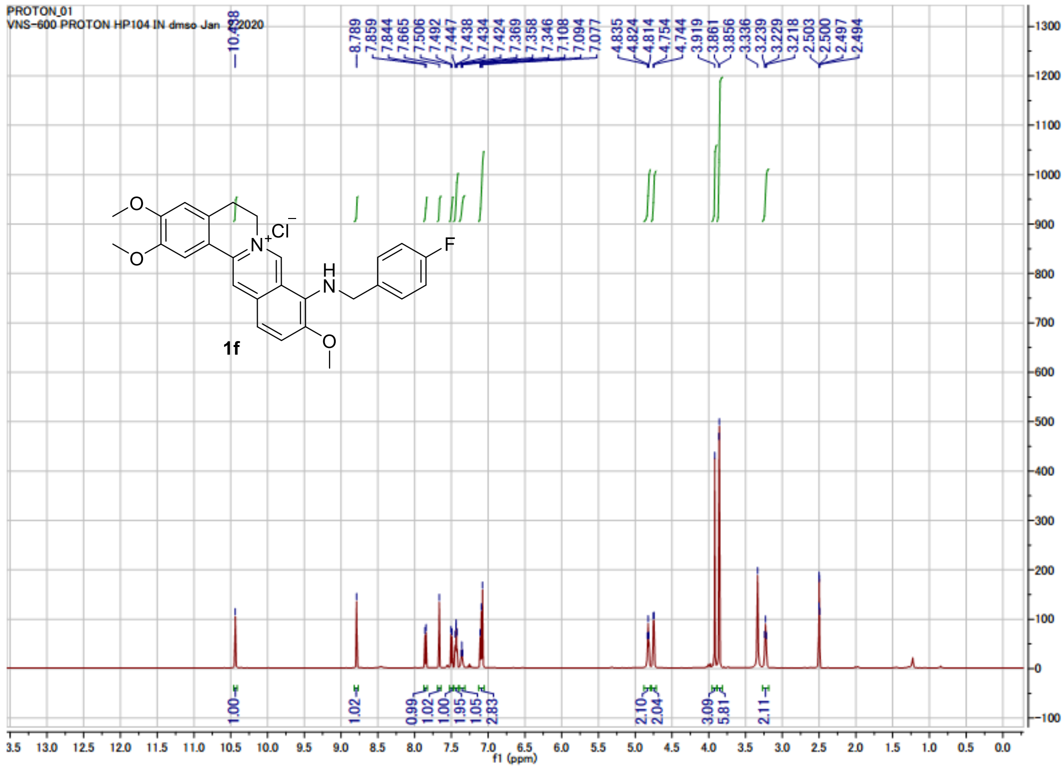

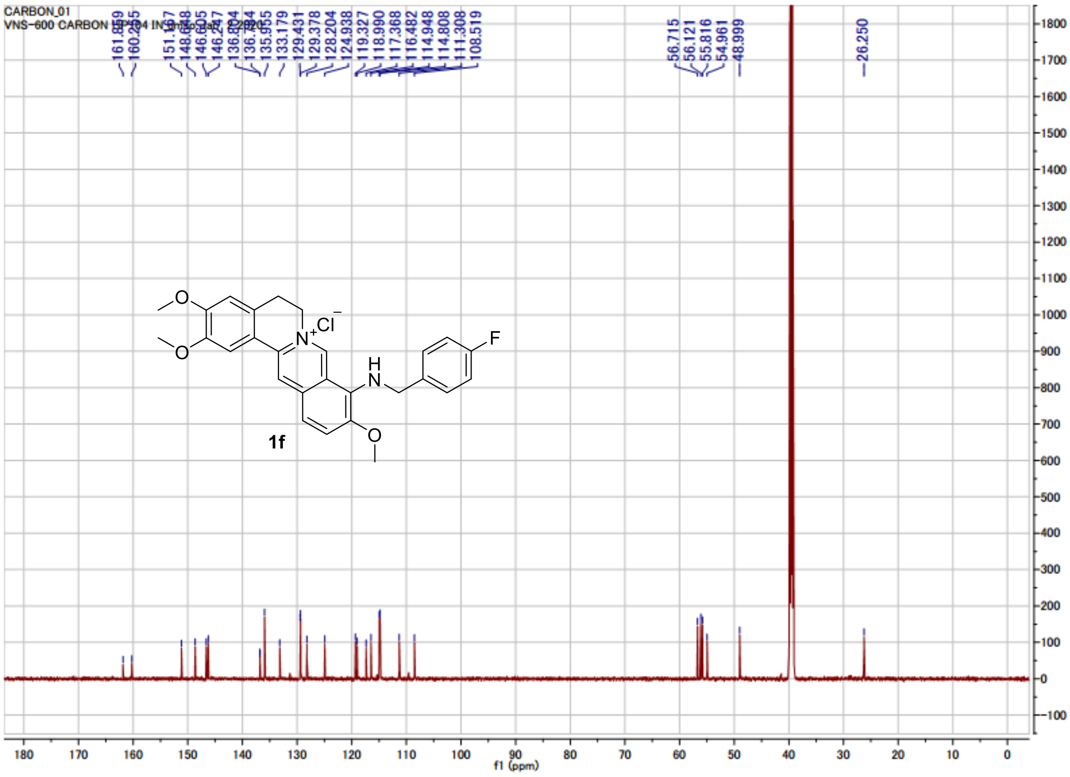

CARBON_01
160.285
146.605
135.955
133.179
129.431
129.378
128.204
124.938
119.327
118.990
117.368
116.482
114.948
114.808
111.308
108.519
56.715
56.121
55.816
54.961
48.999
26.250
f1 (ppm)
1f

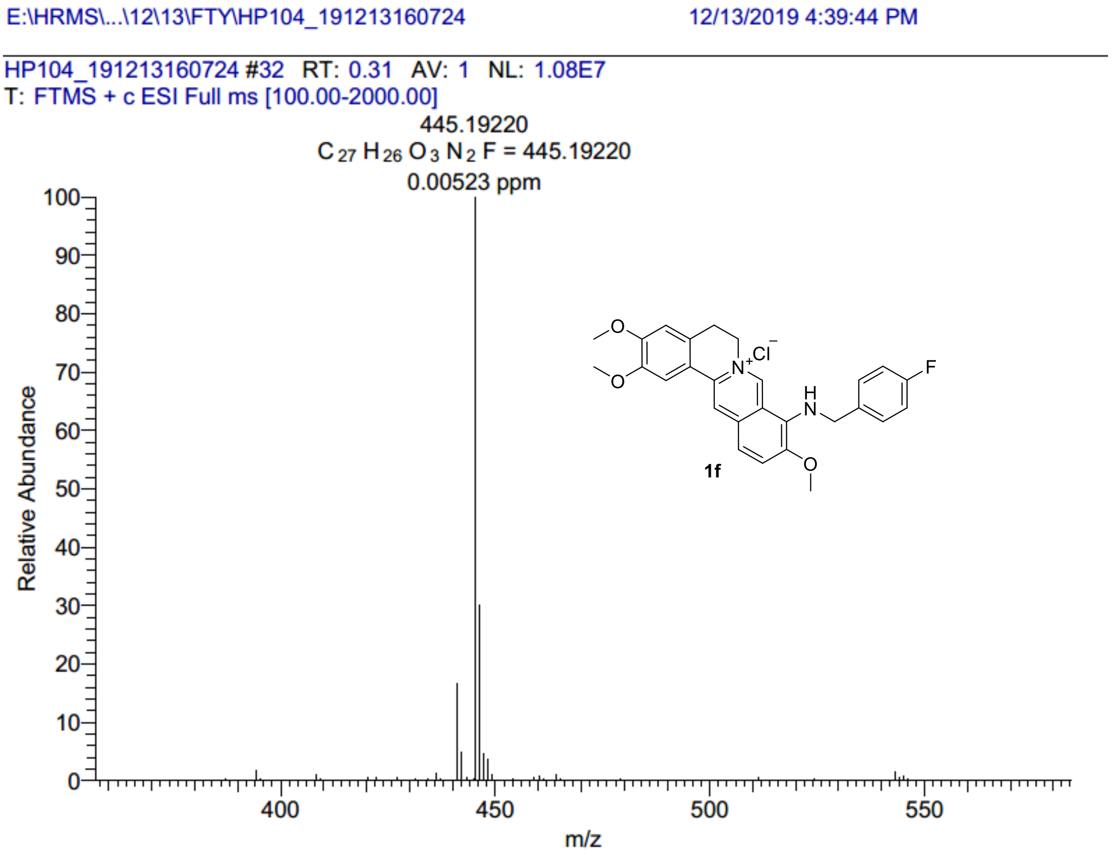

E:\HRMS\...\12\13\FTY\HP104_191213160724
12/13/2019 4:39:44 PM
HP104_191213160724 #32 RT: 0.31 AV: 1 NL: 1.08E7
T: FTMS + c ESI Full ms [100.00-2000.00]
445.19220
C 27 H 26 O 3 N 2 F = 445.19220
0.00523 ppm
Relative Abundance
m/z
1f

Figure S7: ^{1}H NMR, ^{13}C NMR, HRMS-ESI spectra of compound **1g**

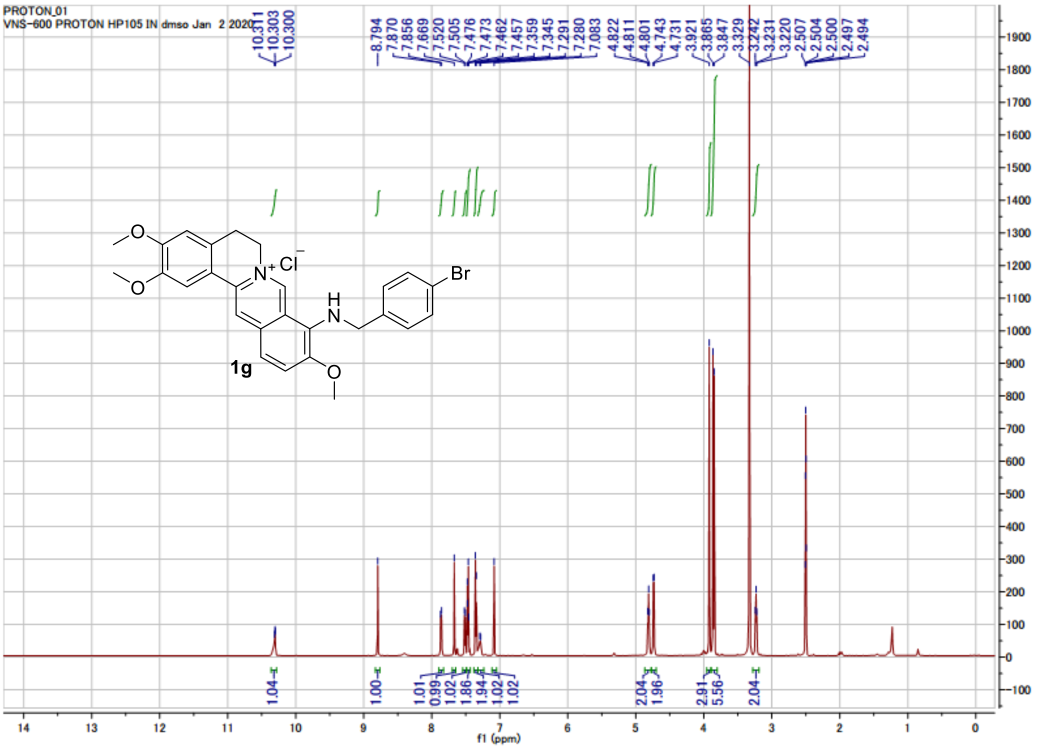

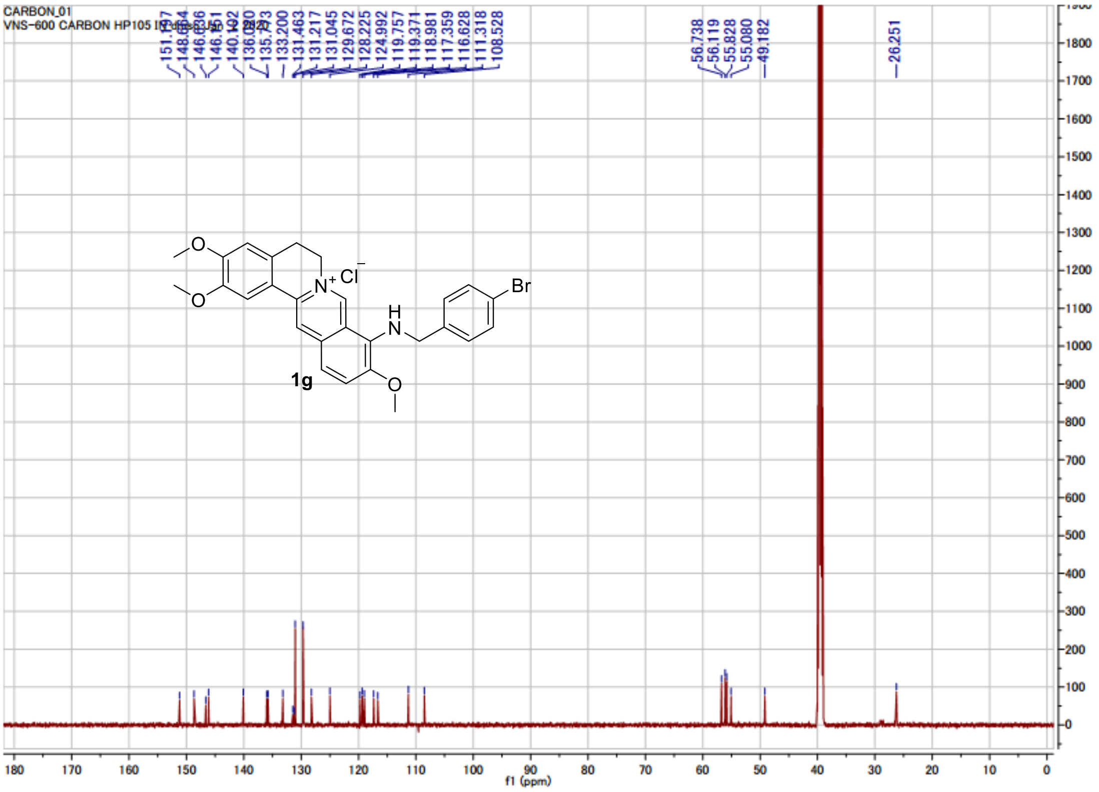

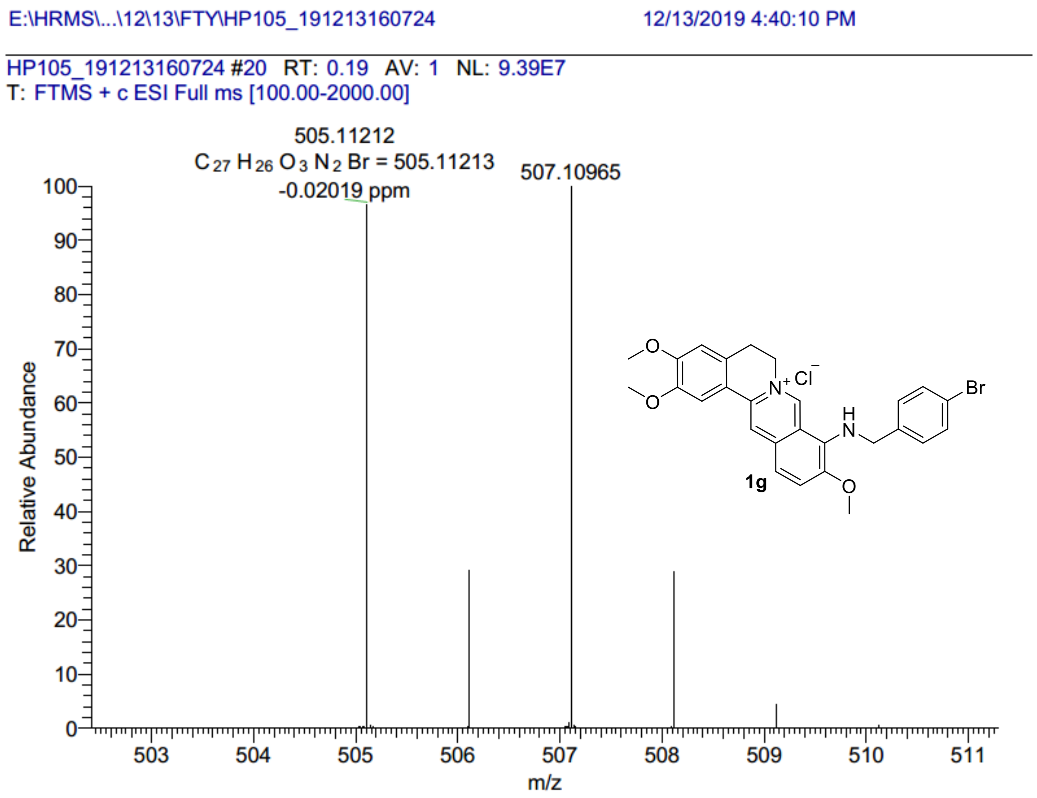

Figure S8: ^{1}H NMR, ^{13}C NMR, HRMS-ESI spectra of compound **1h**

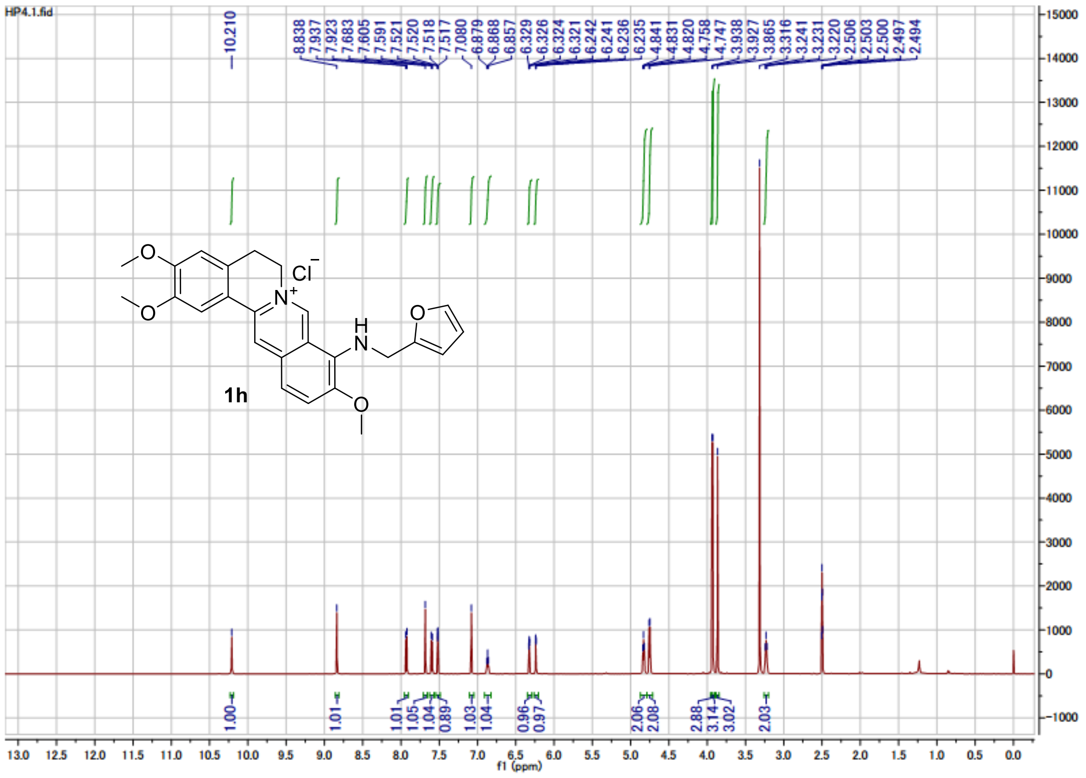

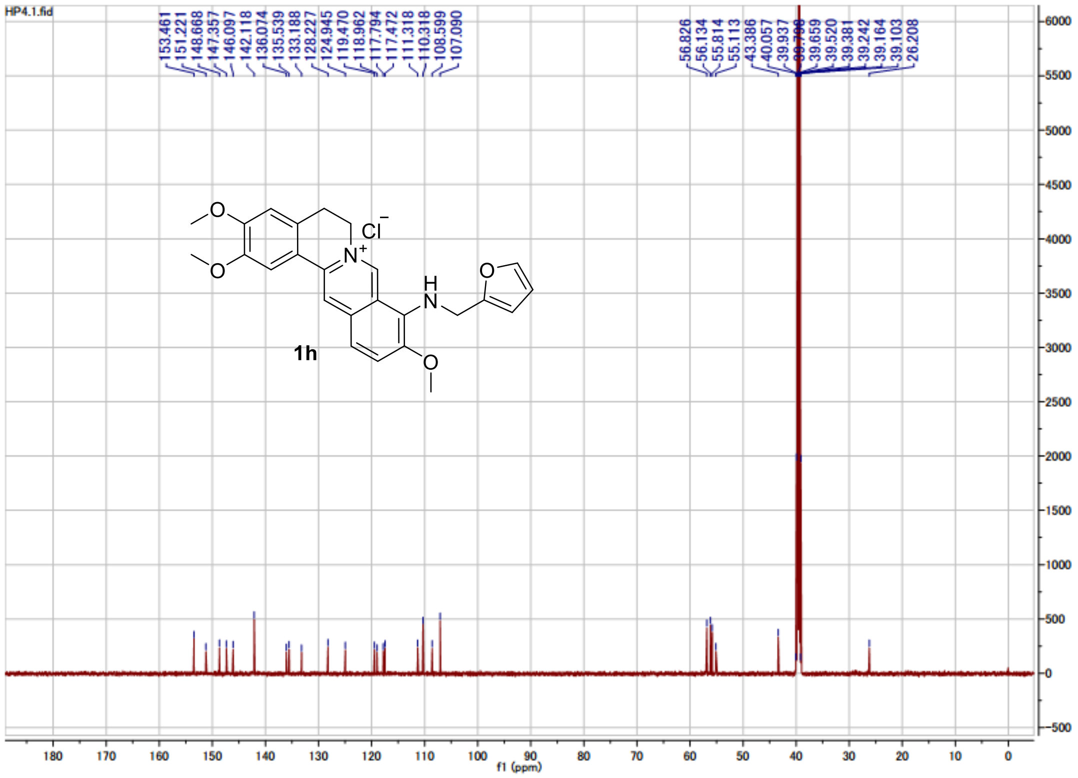

E:\HRMS\2019\09\HP4_ESI+ 9/6/2019 4:36:28 PM

HP4_ESI+ #47 RT: 0.26 AV: 1 SB: 2 0.60 , 0.60 NL: 2.28E8

T: FTMS + c ESI Full ms [100.00-700.00]

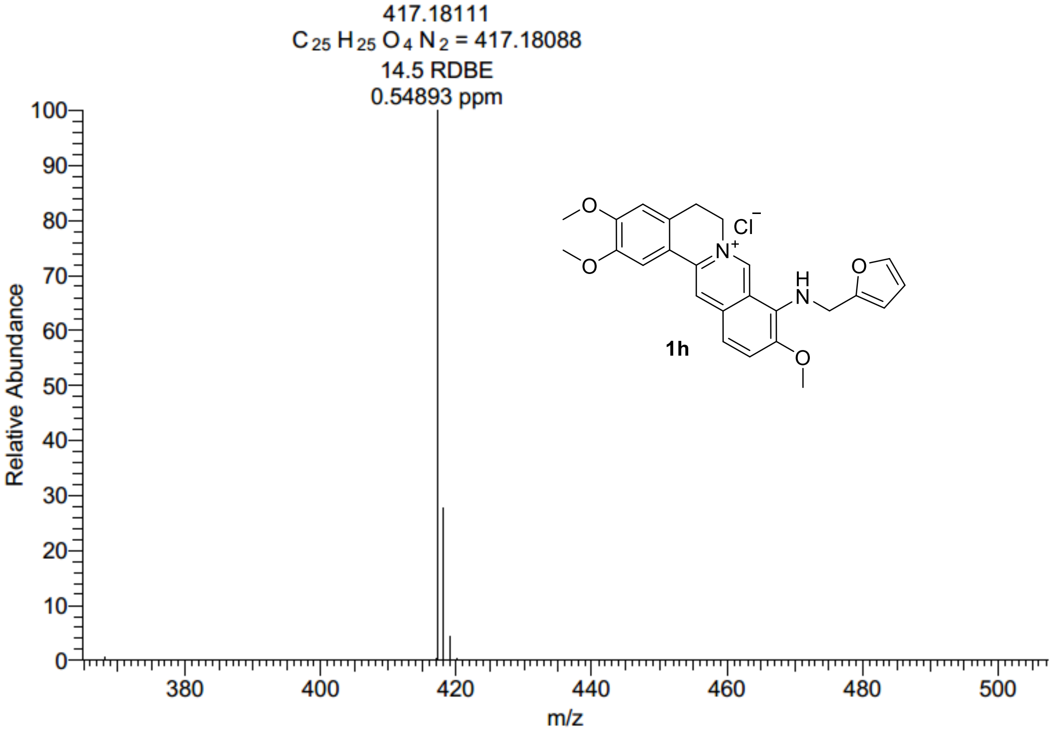

Figure S9: ^{1}H NMR, ^{13}C NMR, HRMS-ESI spectra of compound **1i**

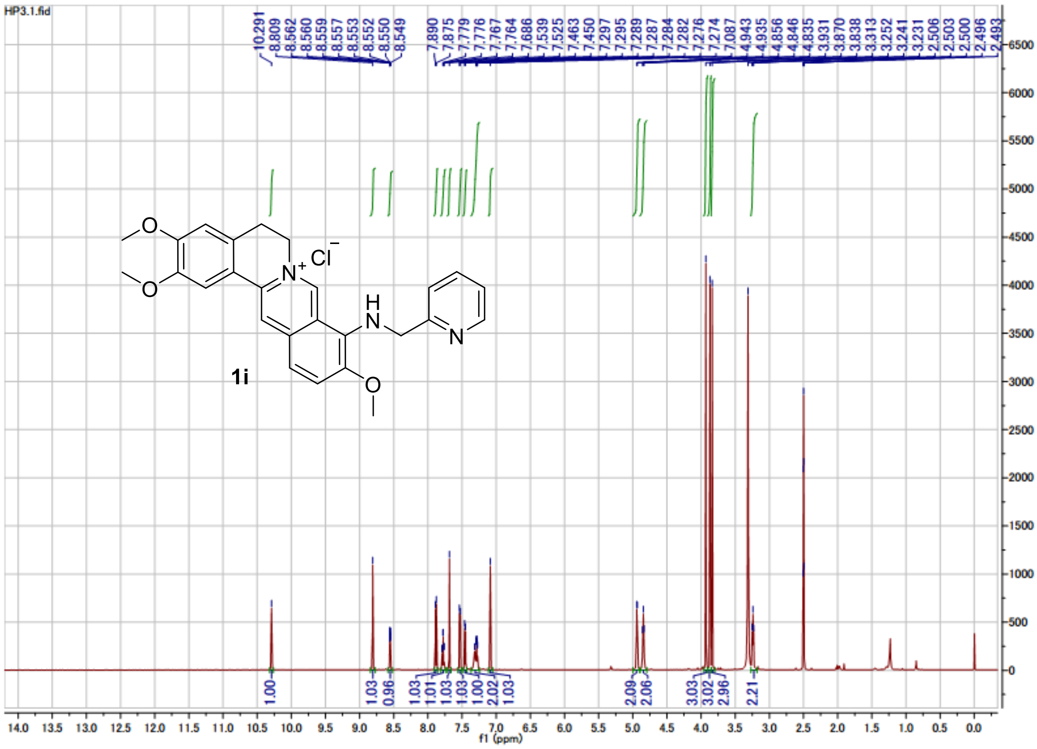

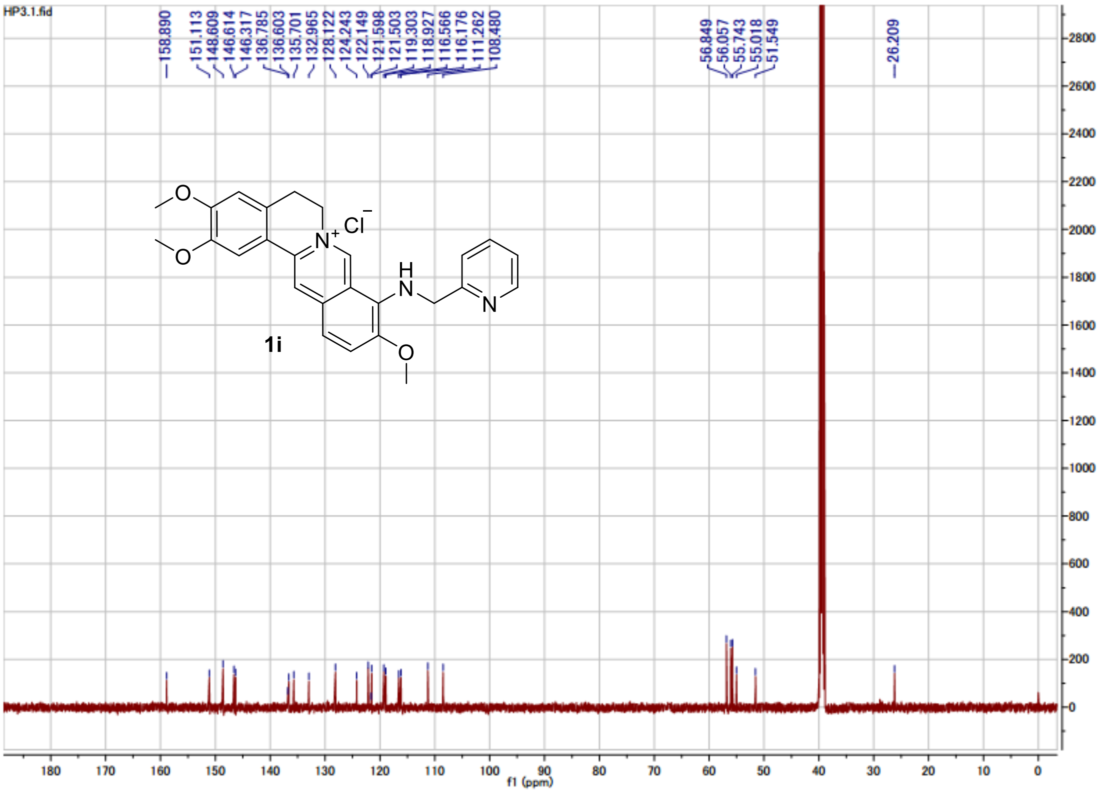

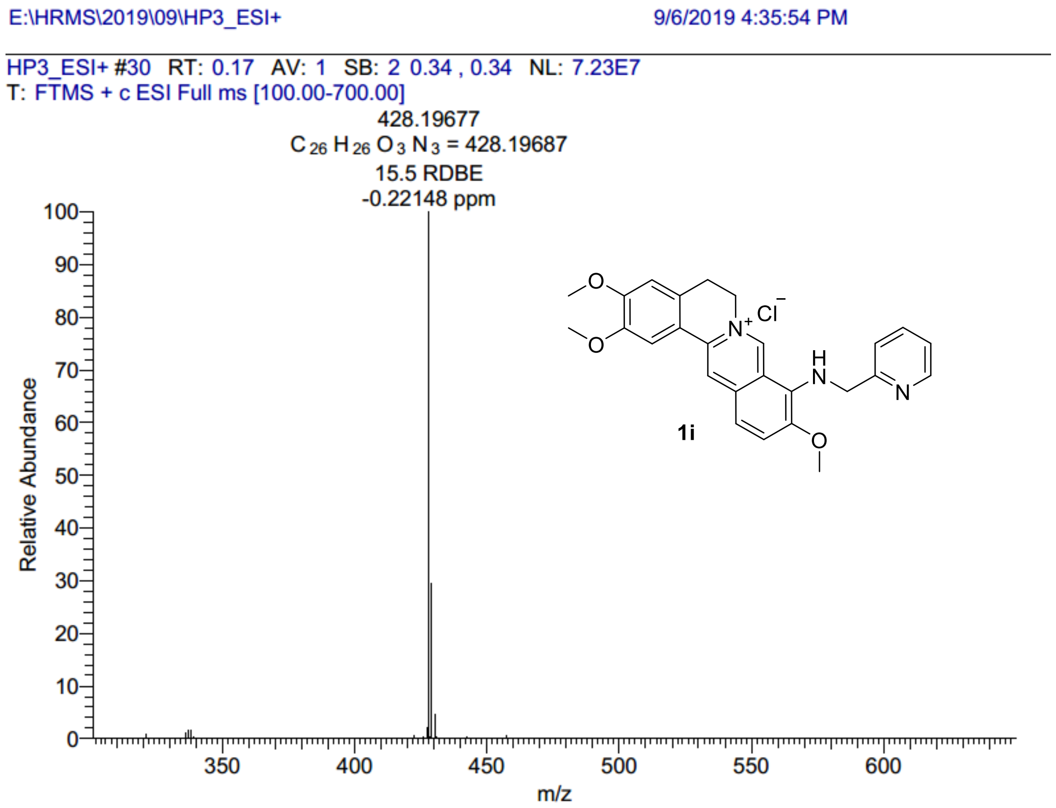

Figure S10: ^{1}H NMR, ^{13}C NMR, HRMS-ESI spectra of compound **2**

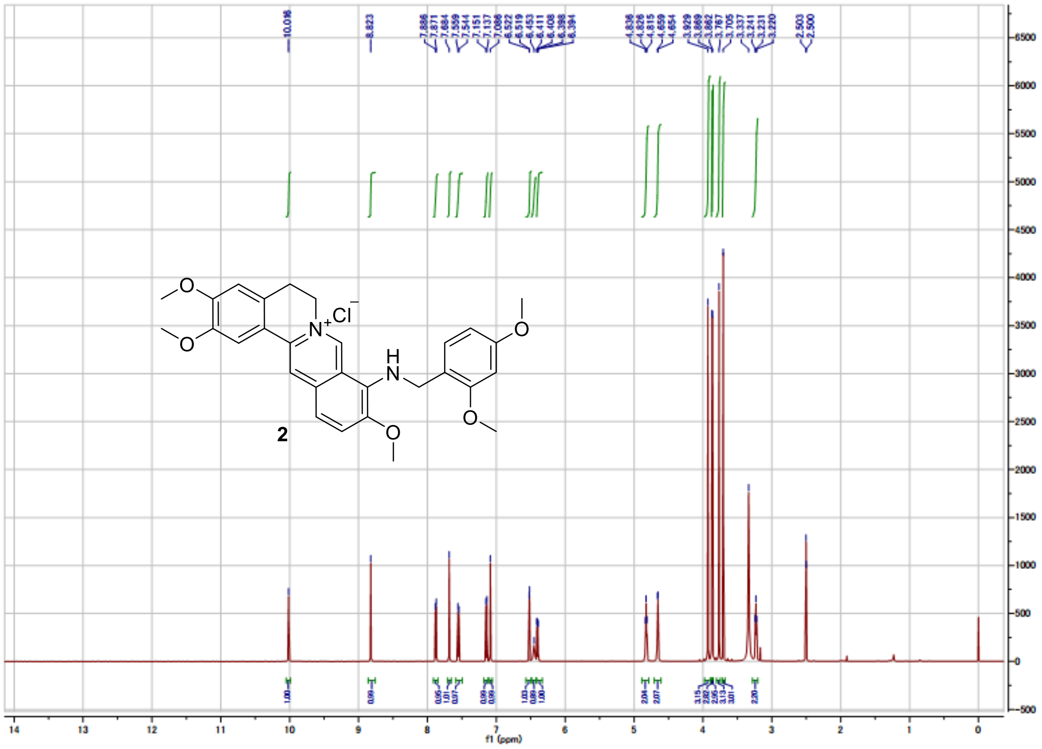

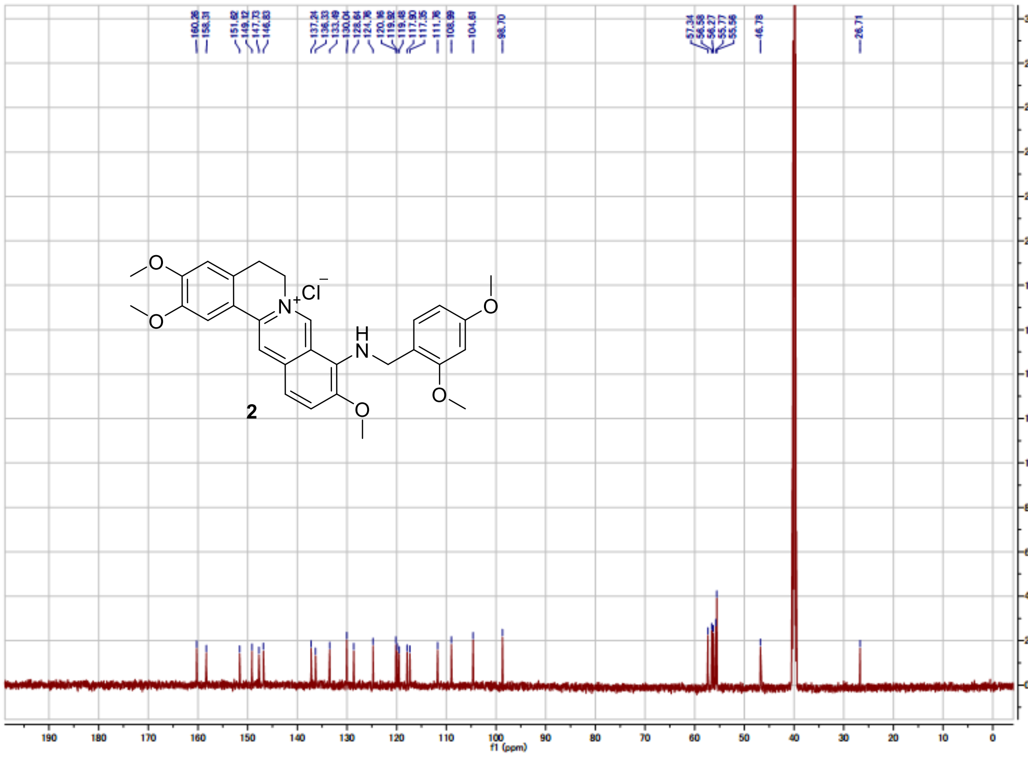

E:\HRMS\2019\12\27\FTY\PN1 12/27/2019 3:34:00 PM

PN1 #35 RT: 0.42 AV: 1 NL: 3.42E8
T: FTMS + c ESI Full ms [100.00-1000.00]

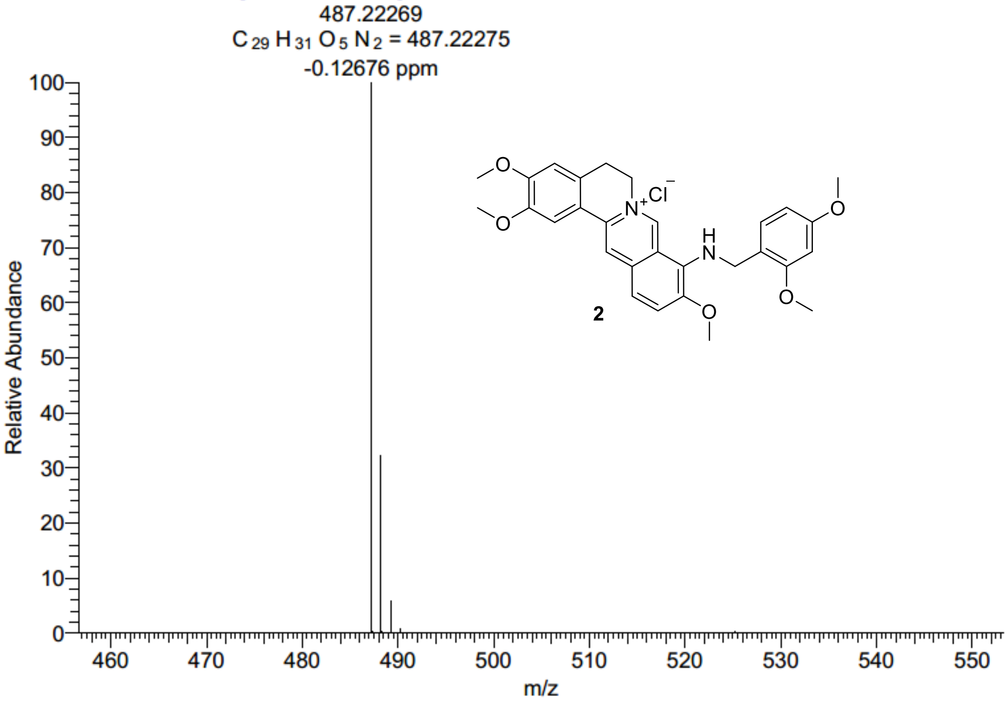

Figure S11: ^{1}H NMR, ^{13}C NMR, HRMS-ESI spectra of compound **3**

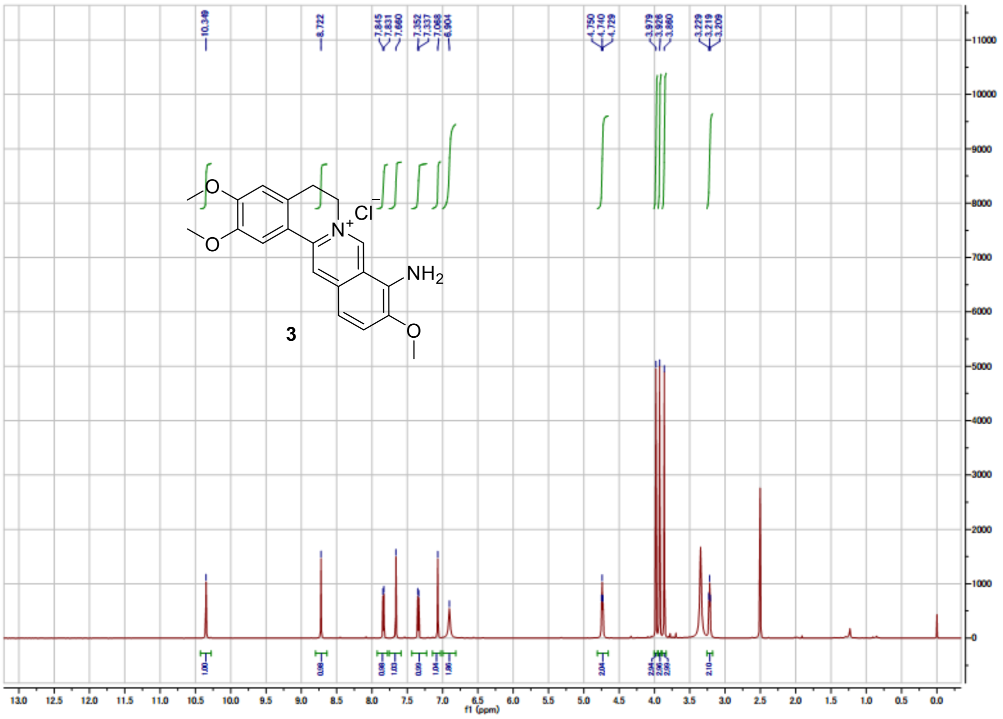

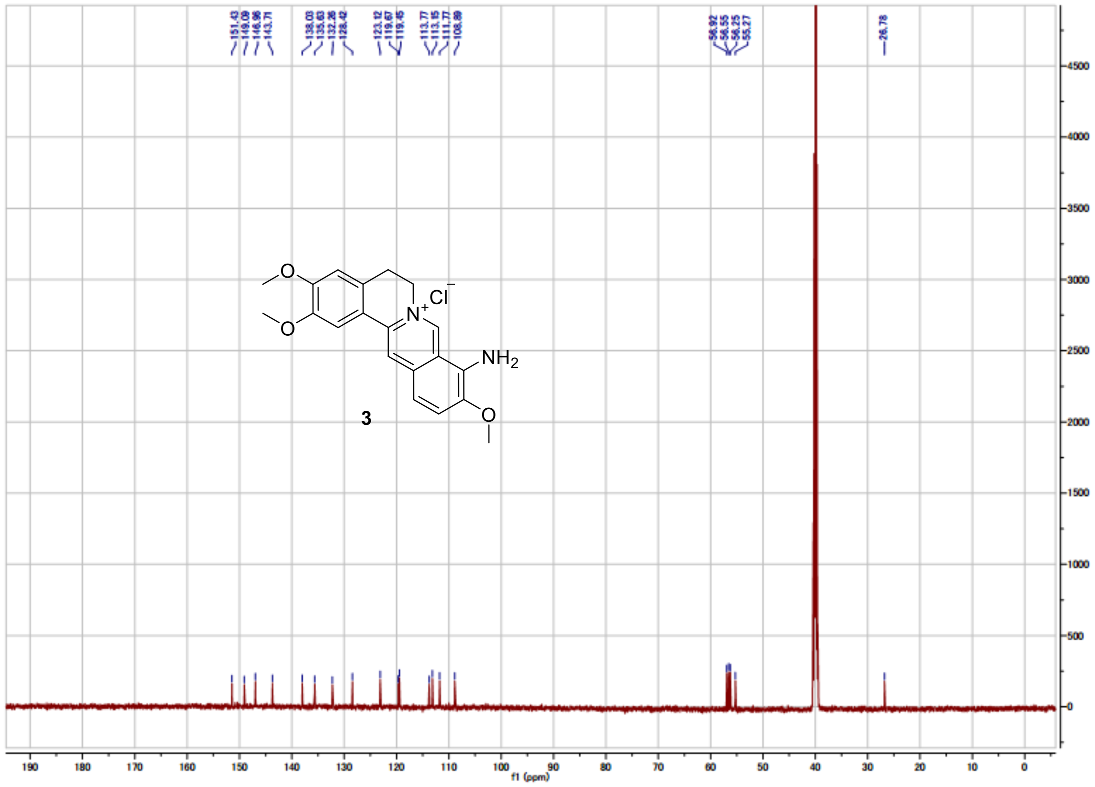

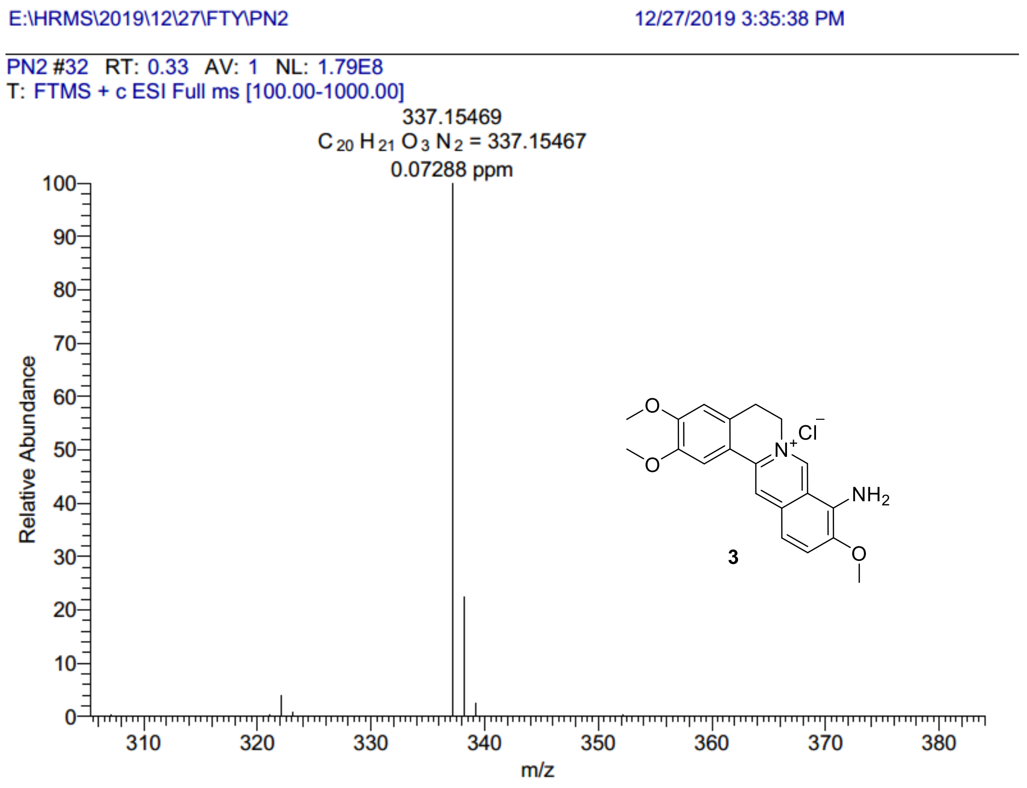

Figure S12: ^{1}H NMR, ^{13}C NMR, HRMS-ESI spectra of compound **4a**

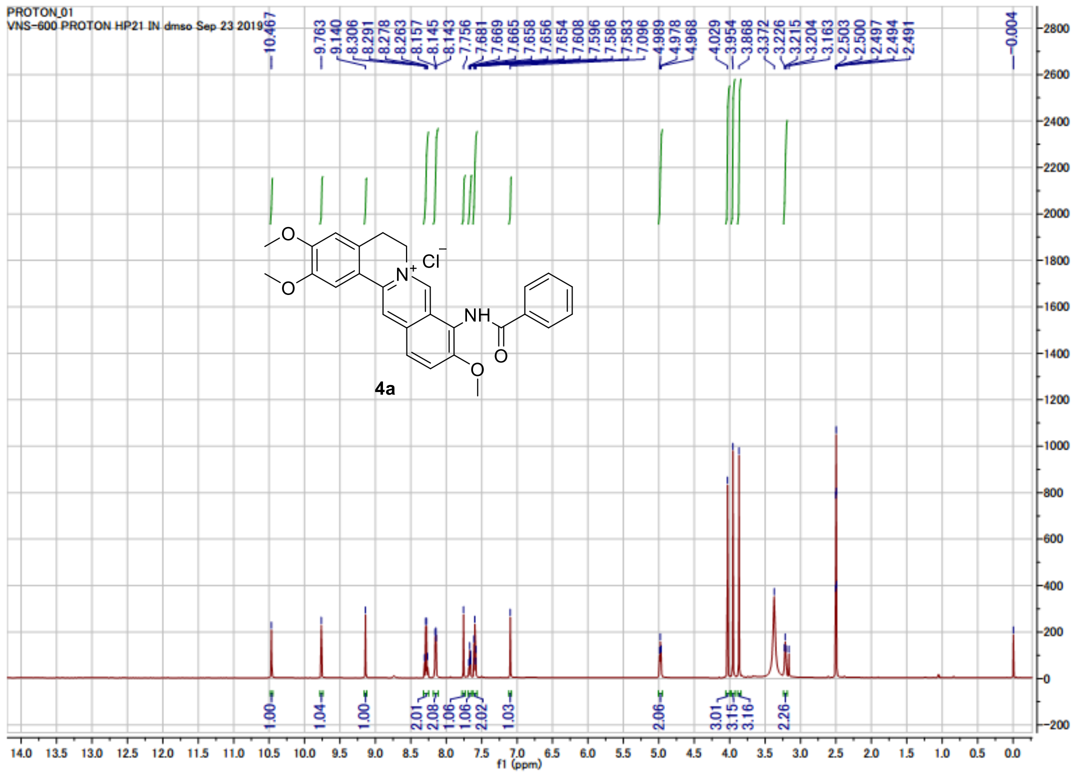

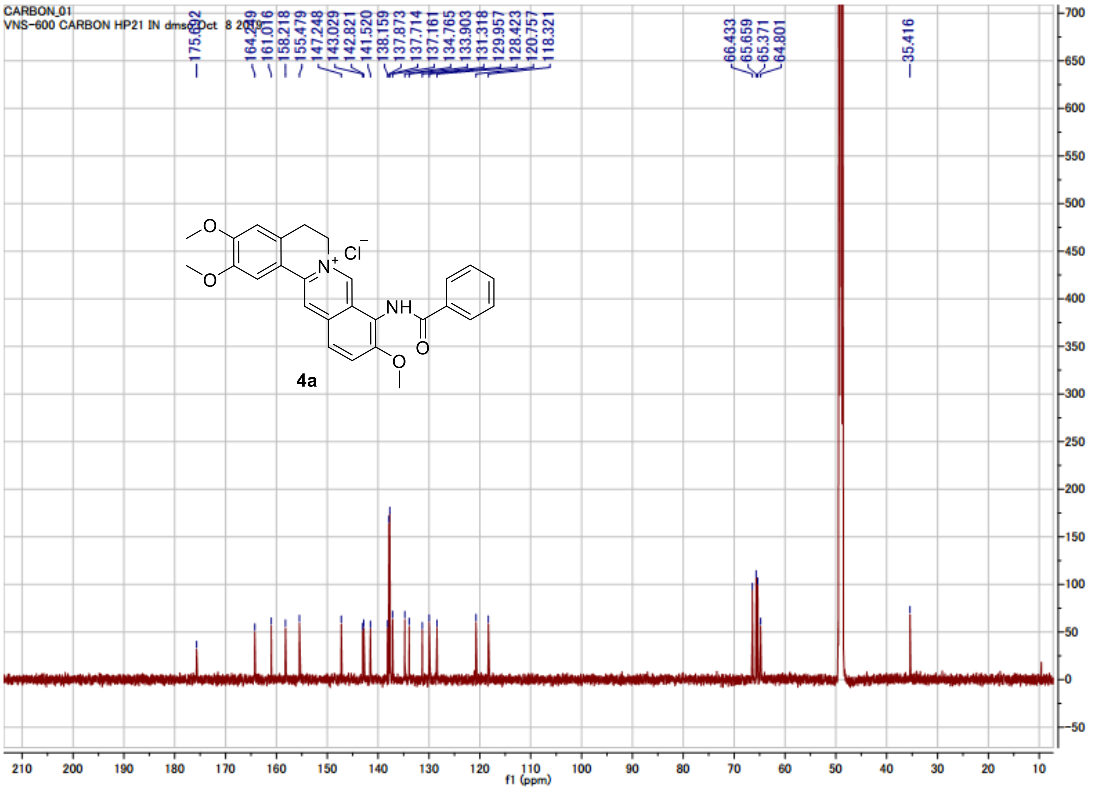
CARBON_01
VNS-600 CARBON HP21 IN dmso Oct 8 2019
161.016
158.218
155.479
147.248
143.029
142.821
141.520
138.159
137.873
137.714
137.161
134.765
133.903
131.318
129.957
128.423
120.757
118.321
66.433
65.659
65.371
64.801
35.416
Cl
NH
O
4a
f1 (ppm)

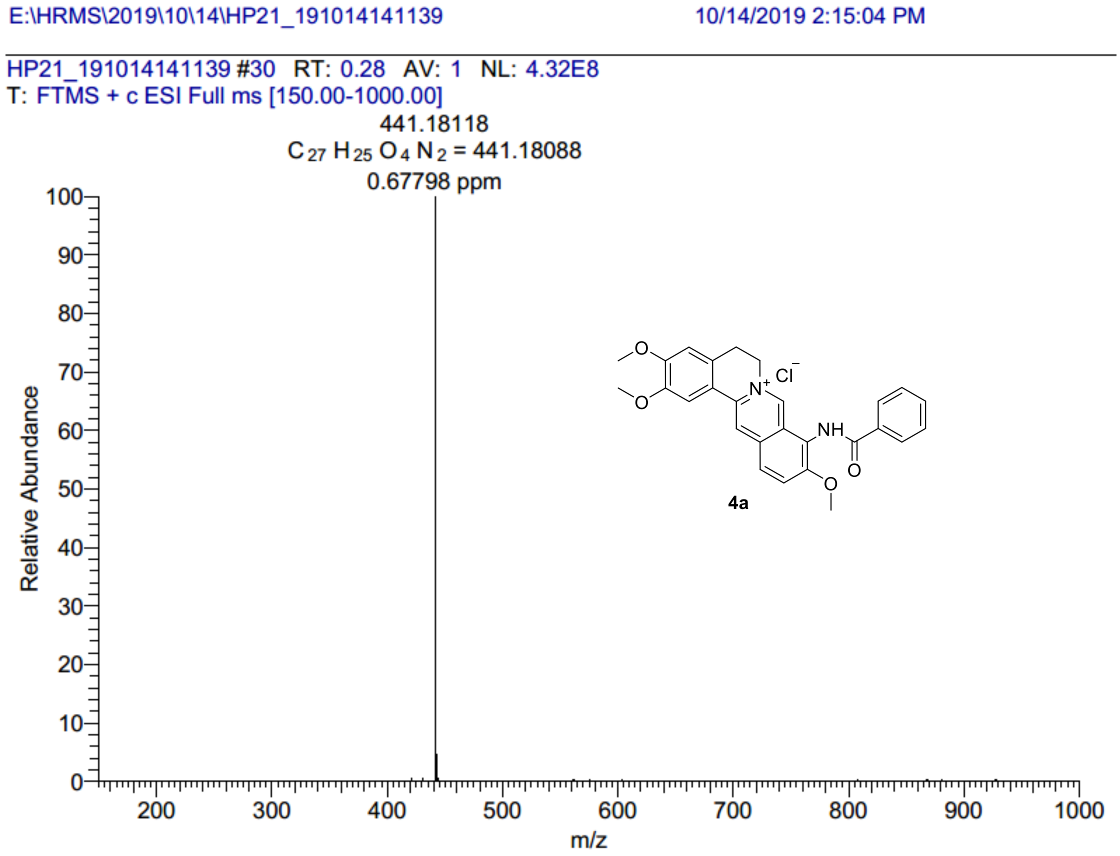
E:\HRMS\2019\10\14\HP21_191014141139
10/14/2019 2:15:04 PM
HP21_191014141139 #30 RT: 0.28 AV: 1 NL: 4.32E8
T: FTMS + c ESI Full ms [150.00-1000.00]
441.18118
C 27 H 25 O 4 N 2 = 441.18088
0.67798 ppm
Relative Abundance
m/z
Cl
NH
O
4a

Figure S13: ^{1}H NMR, ^{13}C NMR, HRMS-ESI spectra of compound **4b**

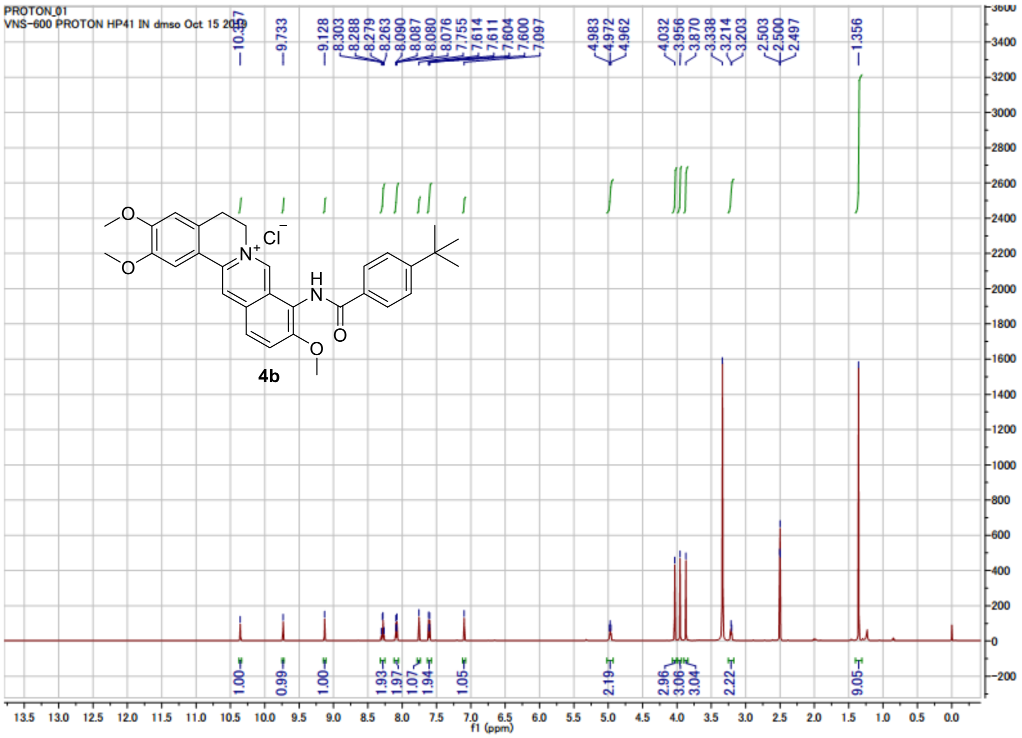

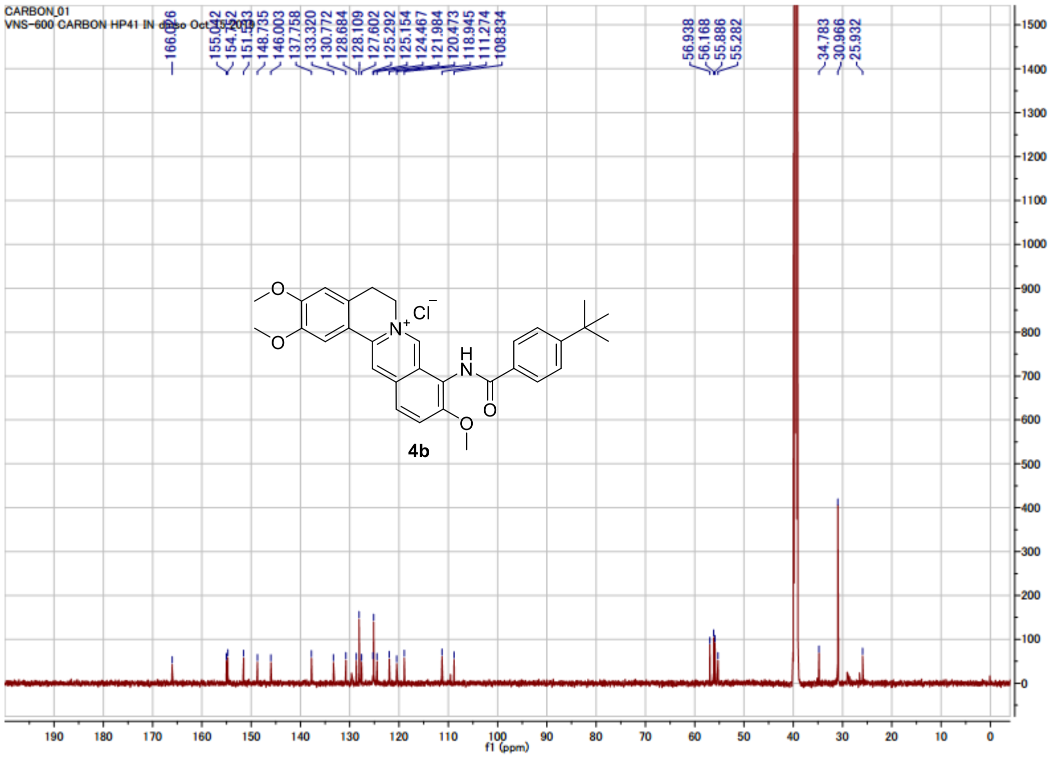

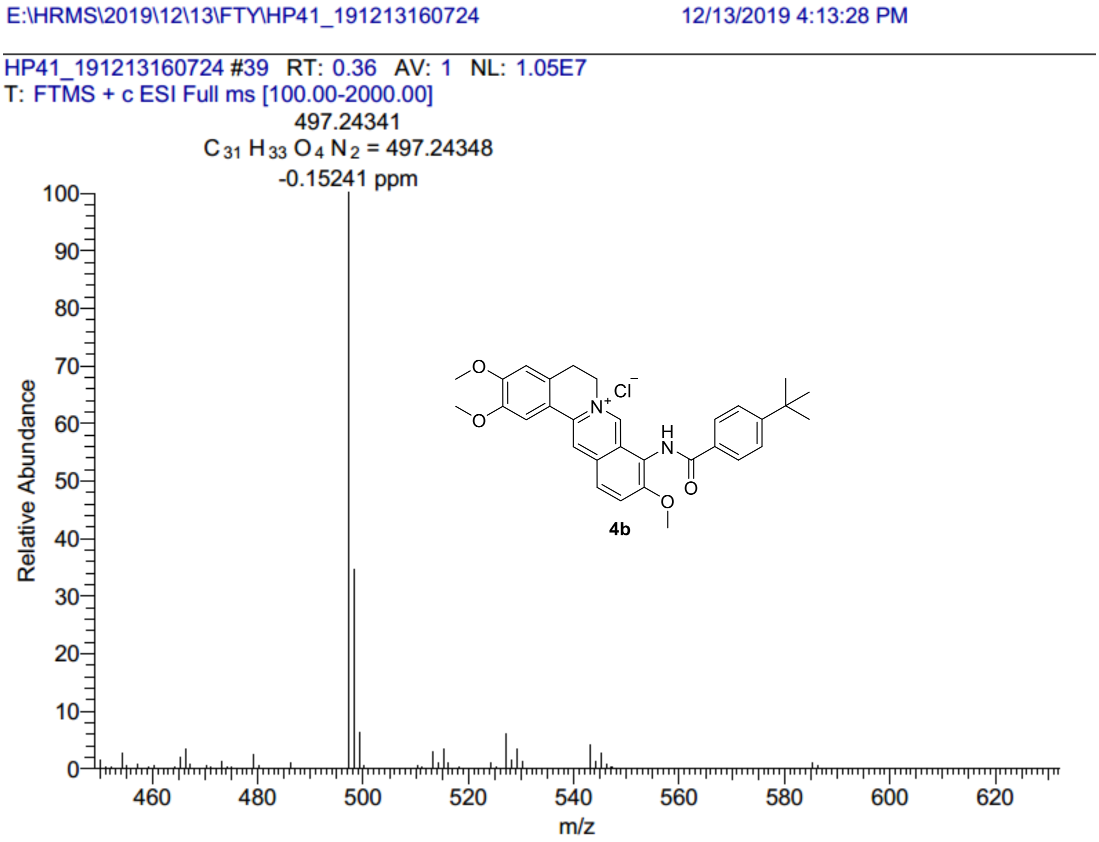

Figure S14: ^{1}H NMR, ^{13}C NMR, HRMS-ESI spectra of compound **4c**

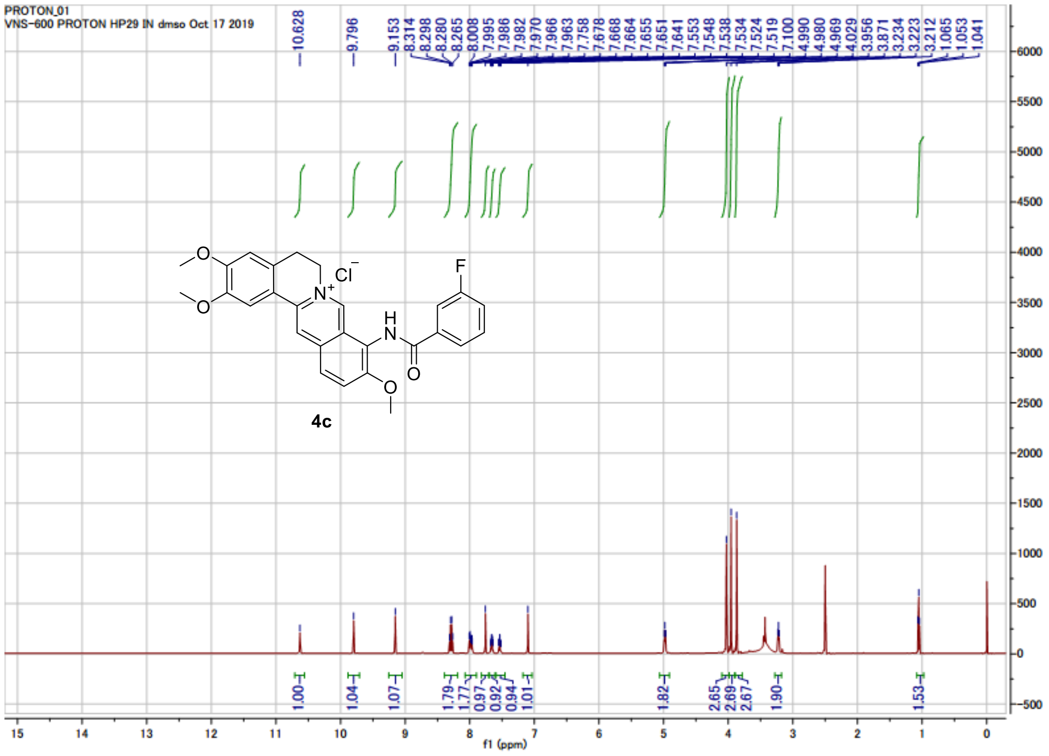

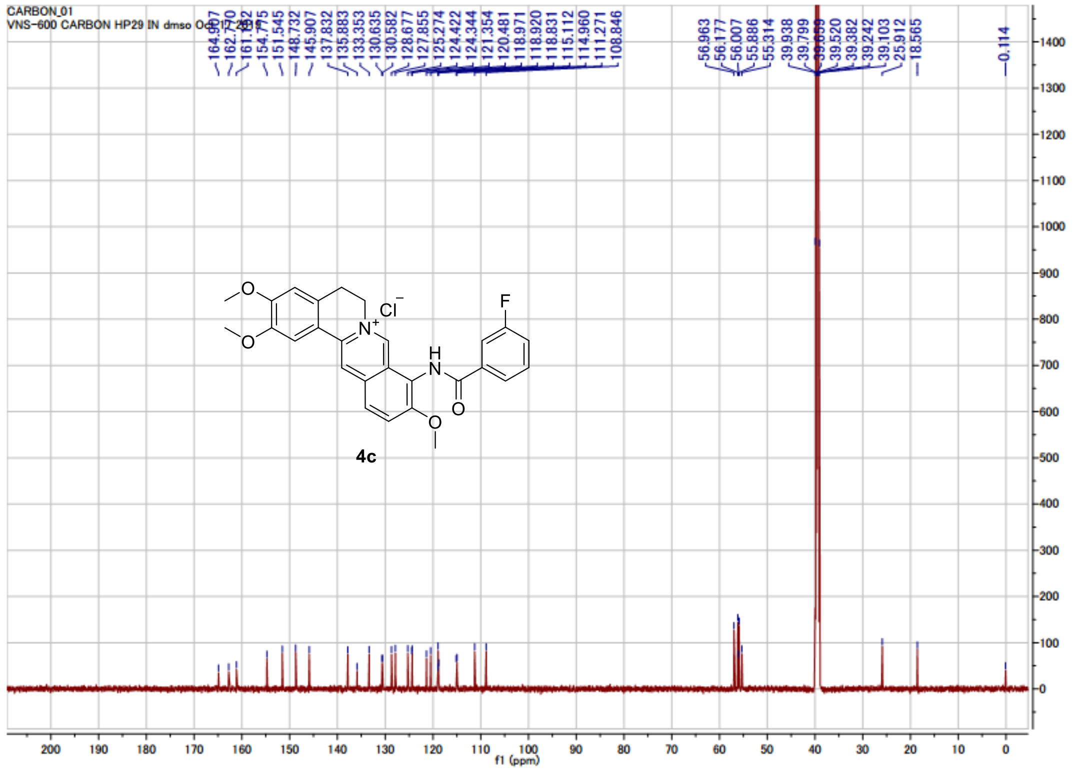

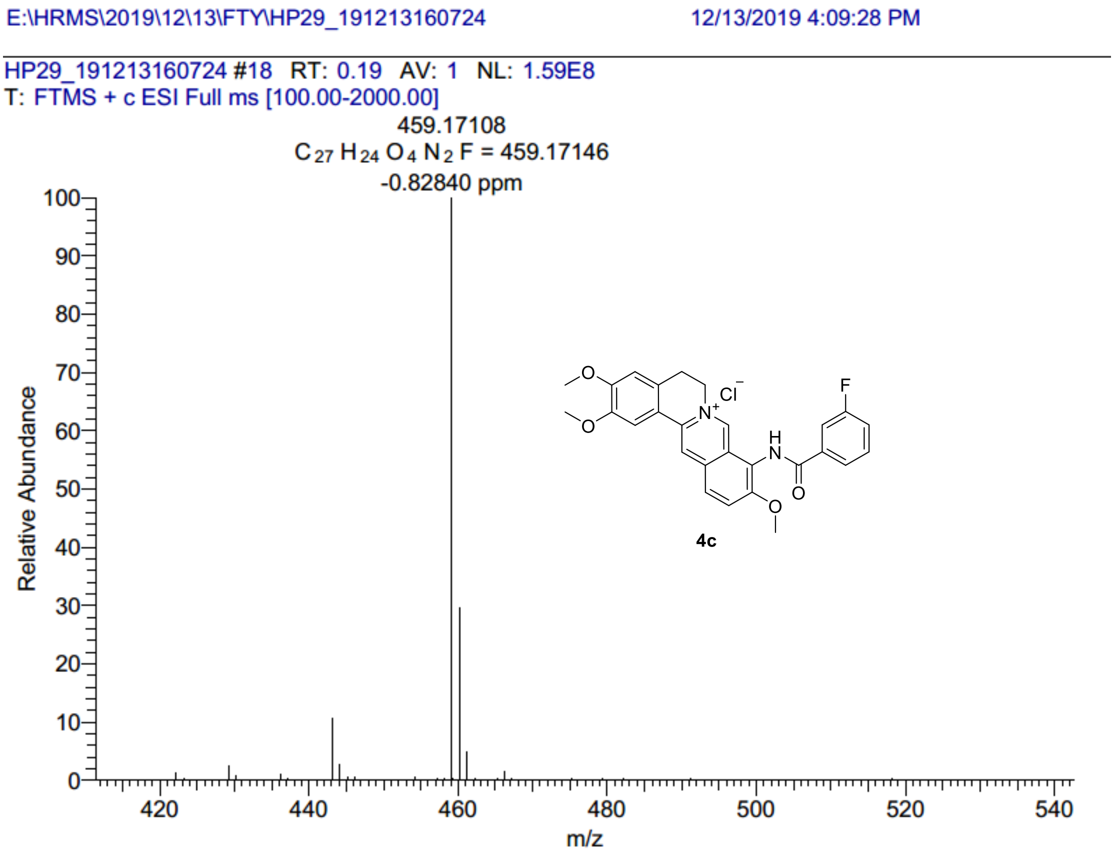

Figure S15: ^{1}H NMR, ^{13}C NMR, HRMS-ESI spectra of compound **4d**

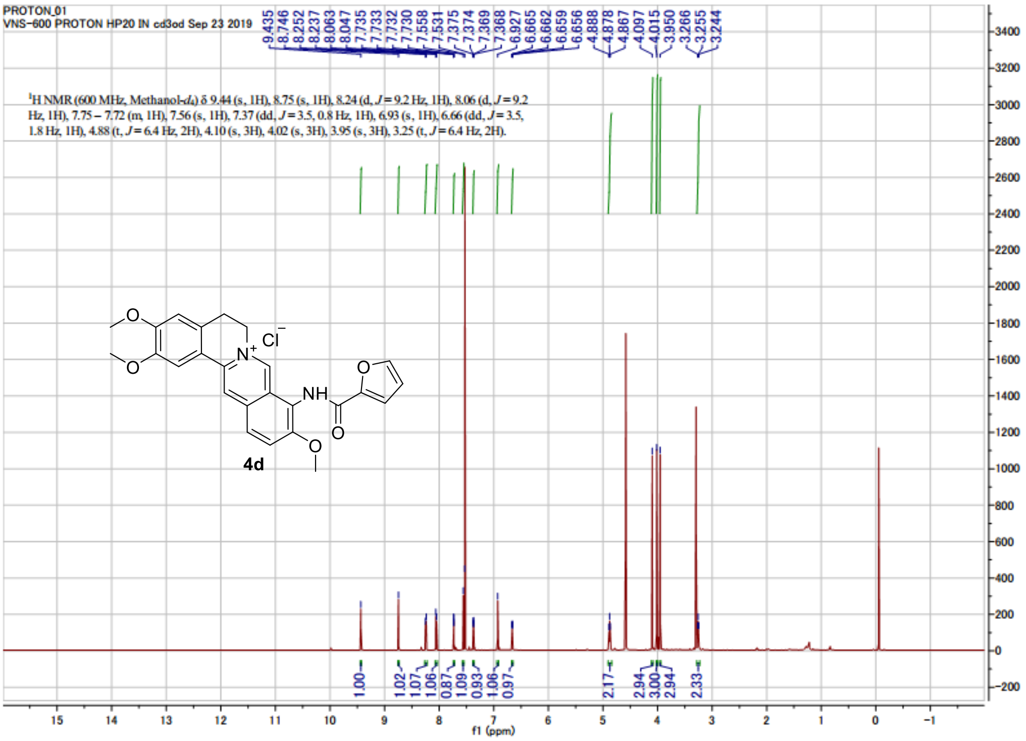
PROTON_01
VNS-600 PROTON HP20 IN cd3od Sep 23 2019
9.435
8.746
8.252
8.237
8.063
8.047
7.735
7.733
7.732
7.730
7.558
7.531
7.375
7.374
7.369
7.368
6.927
6.665
6.662
6.659
6.656
4.888
4.878
4.867
4.097
4.015
3.950
3.266
3.255
3.244
1H NMR (600 MHz, Methanol-d4) δ 9.44 (s, 1H), 8.75 (s, 1H), 8.24 (d, J = 9.2 Hz, 1H), 8.06 (d, J = 9.2 Hz, 1H), 7.75 – 7.72 (m, 1H), 7.56 (s, 1H), 7.37 (dd, J = 3.5, 0.8 Hz, 1H), 6.93 (s, 1H), 6.66 (dd, J = 3.5, 1.8 Hz, 1H), 4.88 (t, J = 6.4 Hz, 2H), 4.10 (s, 3H), 4.02 (s, 3H), 3.95 (s, 3H), 3.25 (t, J = 6.4 Hz, 2H).
4d
1.00
1.02
1.07
1.06
0.87
1.09
0.93
1.06
0.97
2.17
2.94
3.00
2.94
2.33
f1 (ppm)

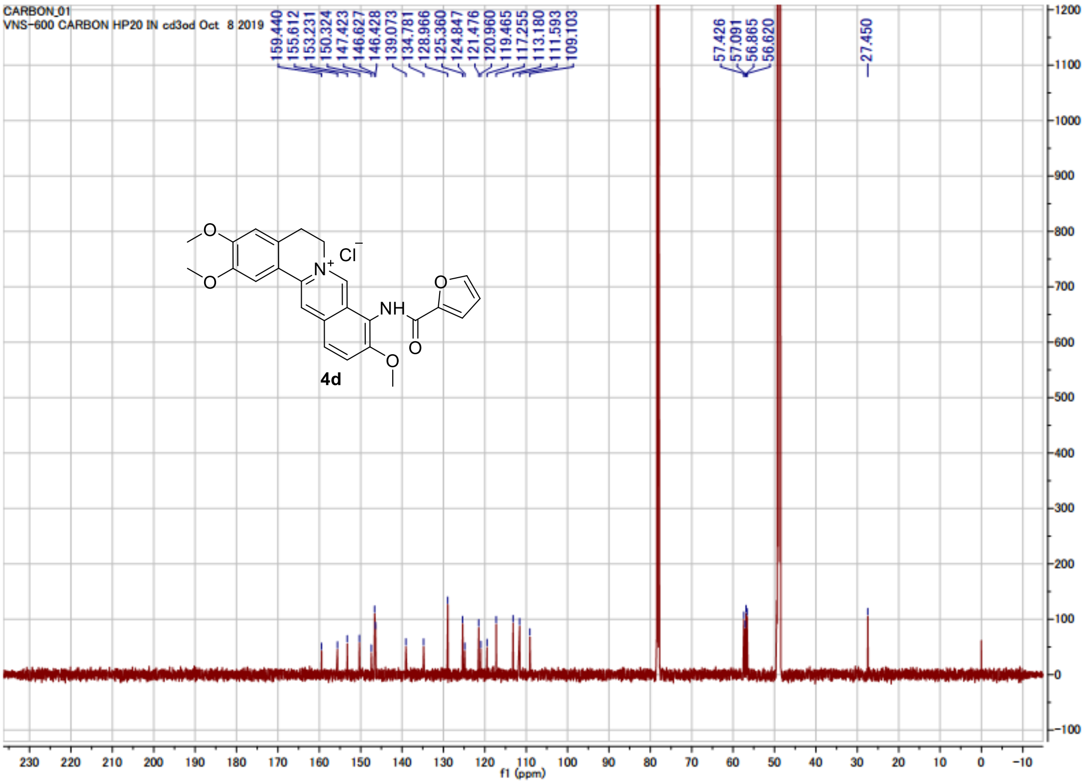
CARBON_01
VNS-600 CARBON HP20 IN cd3od Oct 8 2019
159.440
155.612
153.231
150.324
147.423
146.627
146.428
139.073
134.781
128.966
125.360
124.847
121.476
120.960
119.465
117.255
113.180
111.593
109.103
57.426
57.091
56.865
56.620
27.450
4d
f1 (ppm)

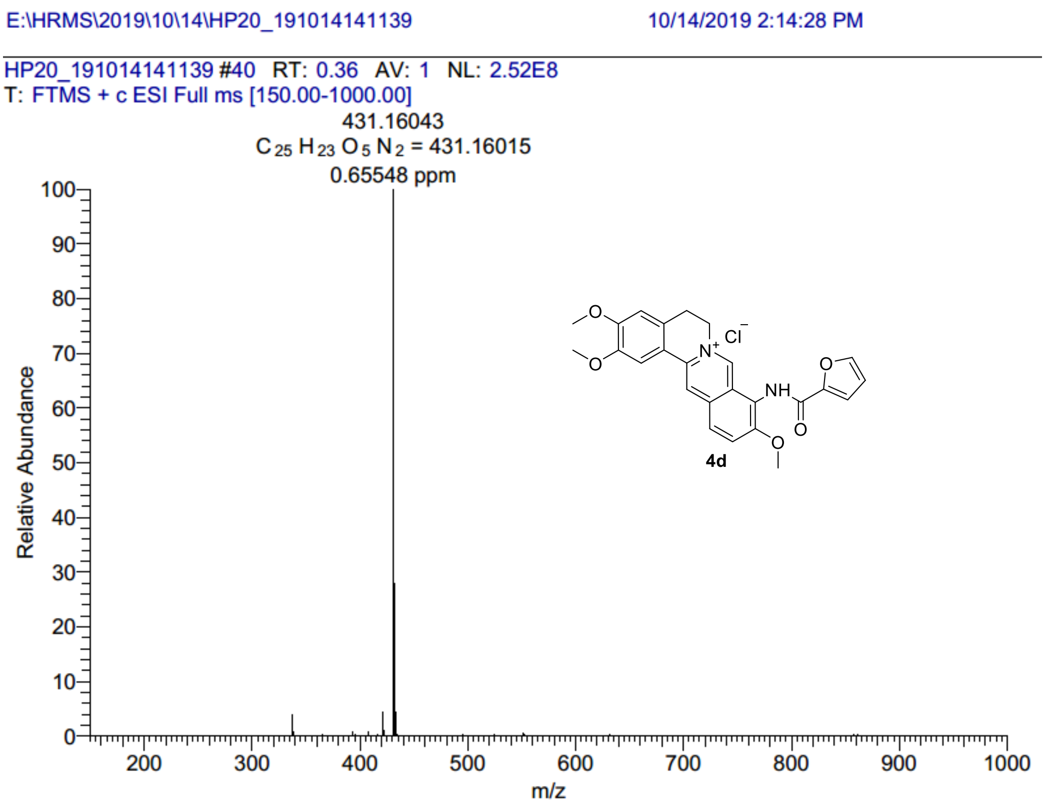

Figure S16: ^{1}H NMR, ^{13}C NMR, HRMS-ESI spectra of compound **4e**

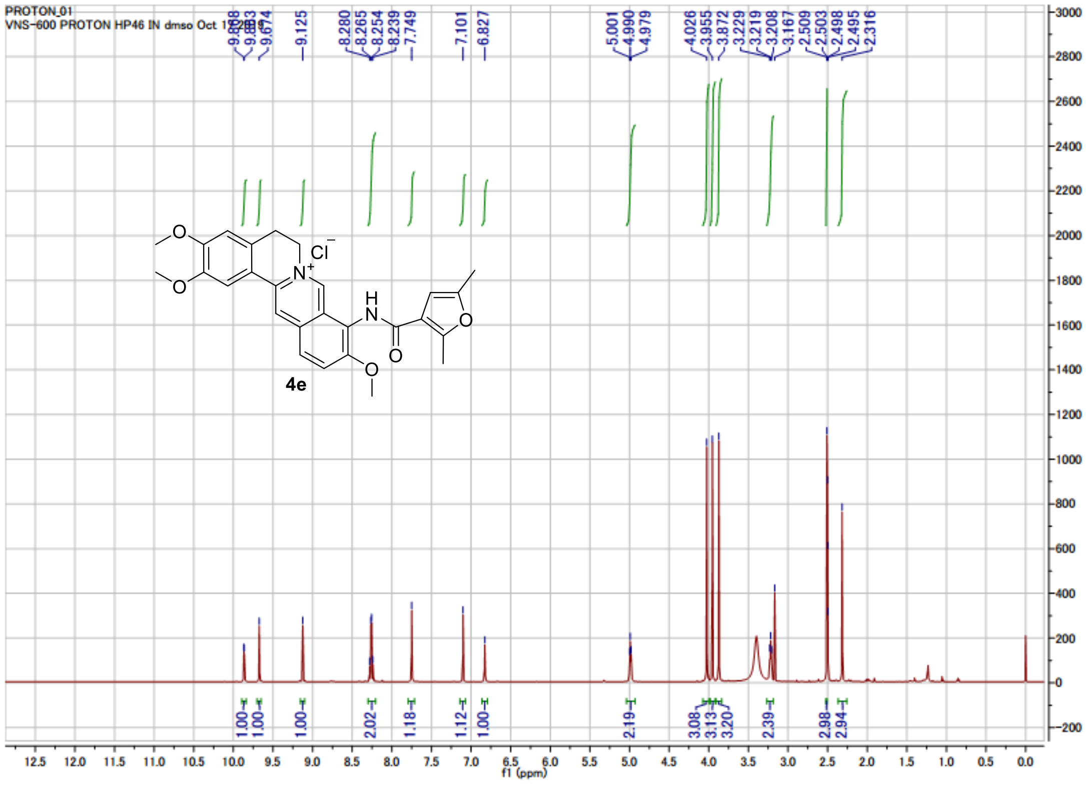

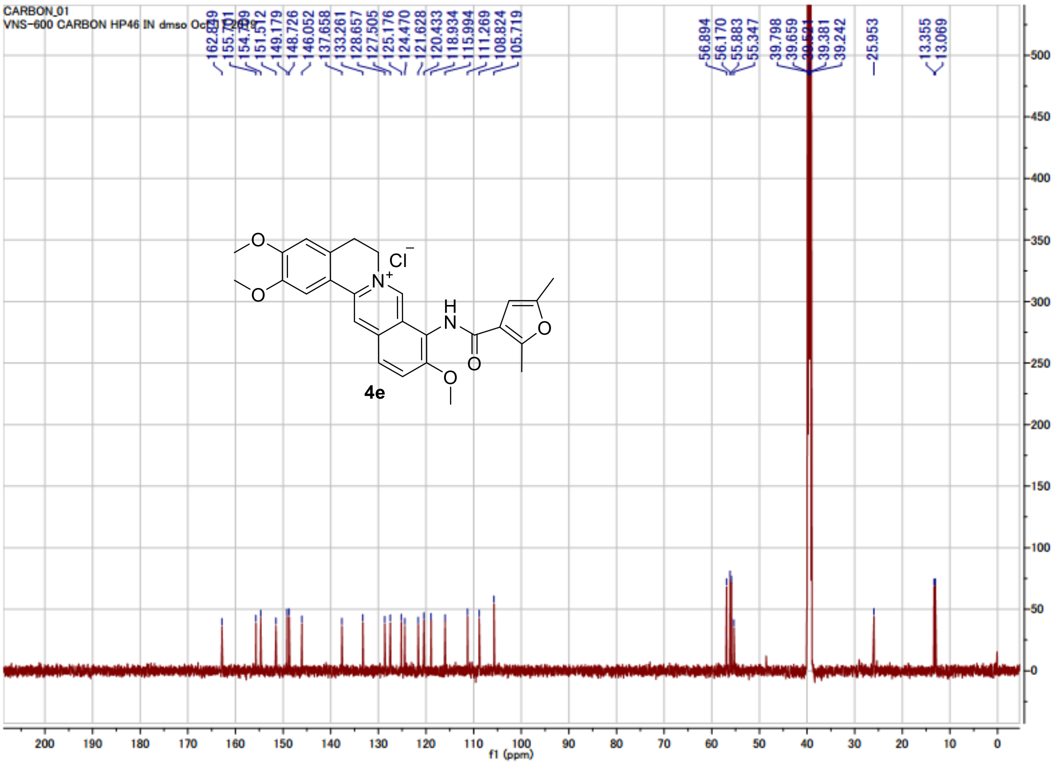

E:\HRMS\2019\12\13\FTY\HP46_191213160724 12/13/2019 4:19:49 PM

HP46_191213160724 #14 RT: 0.14 AV: 1 NL: 1.90E8
T: FTMS + c ESI Full ms [100.00-2000.00]

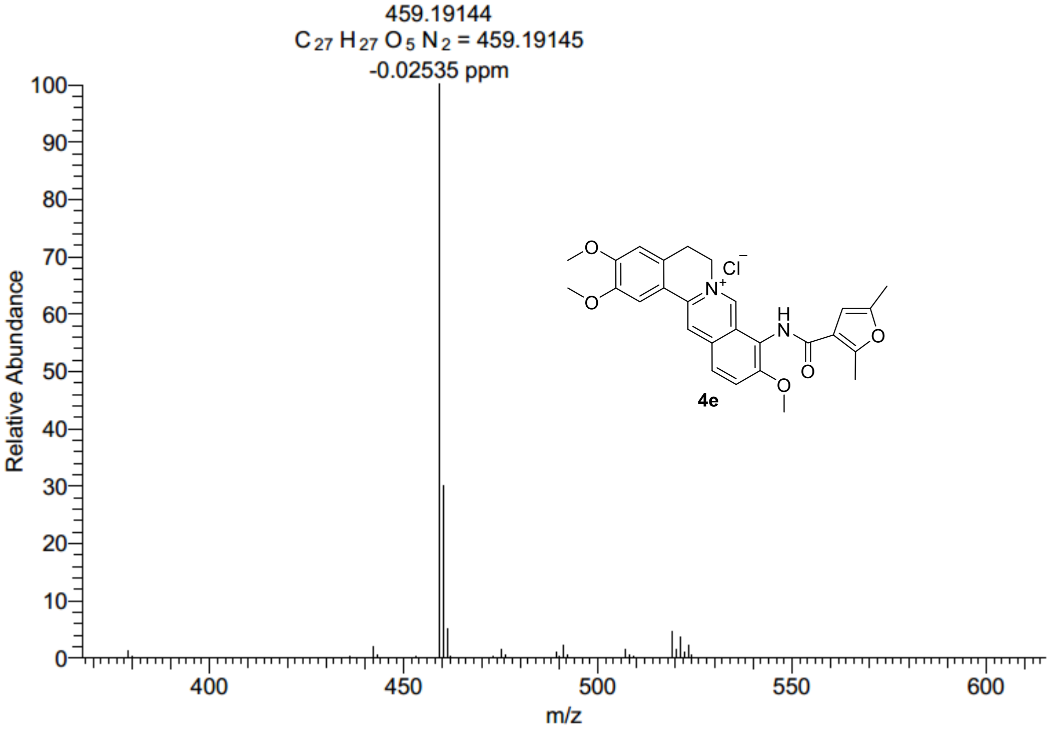

Figure S17: ^{1}H NMR, ^{13}C NMR, HRMS-ESI spectra of compound **4f**

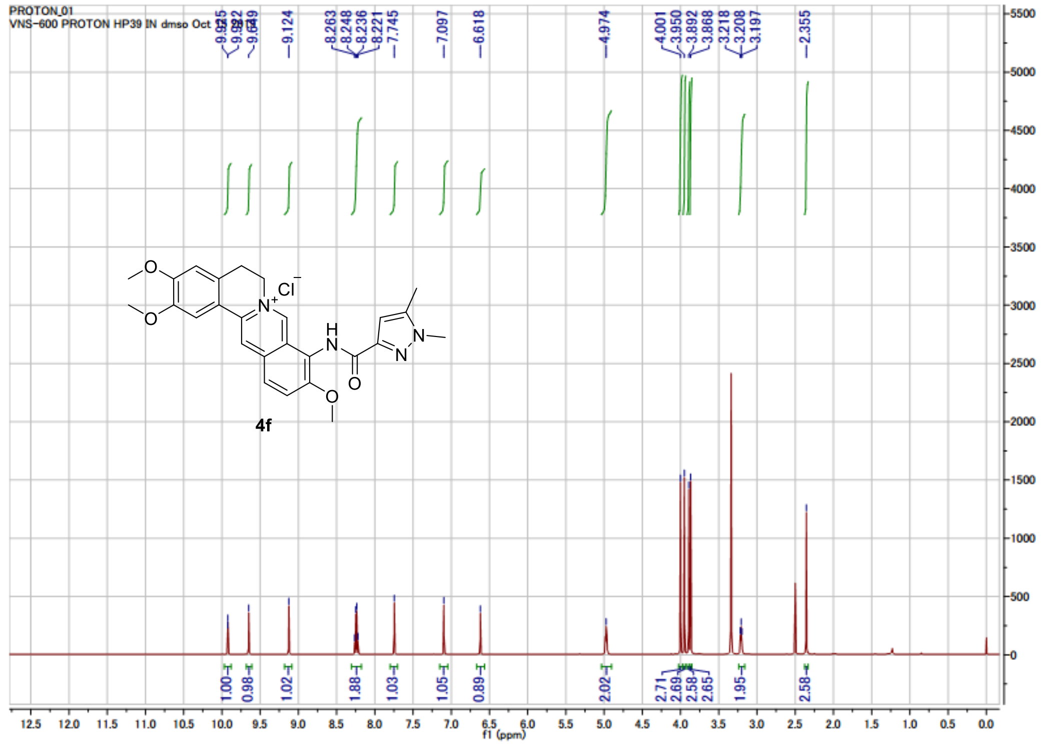

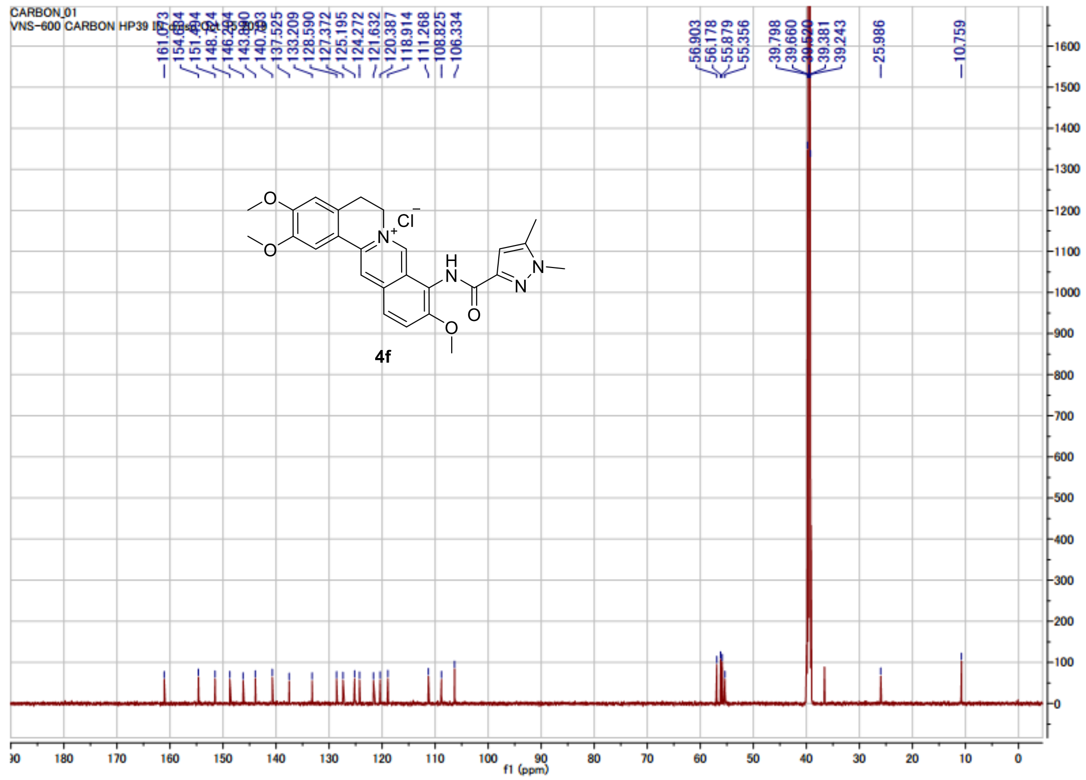

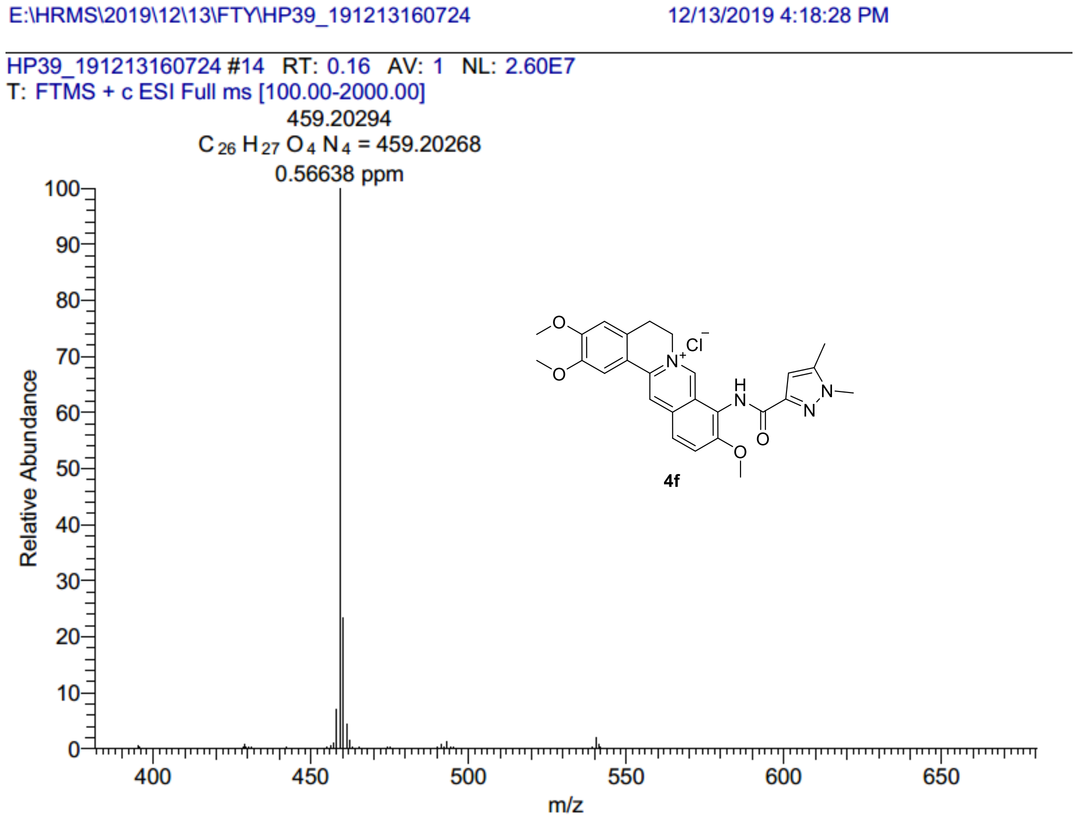

Figure S18: ^{1}H NMR, ^{13}C NMR, HRMS-ESI spectra of compound **4g**

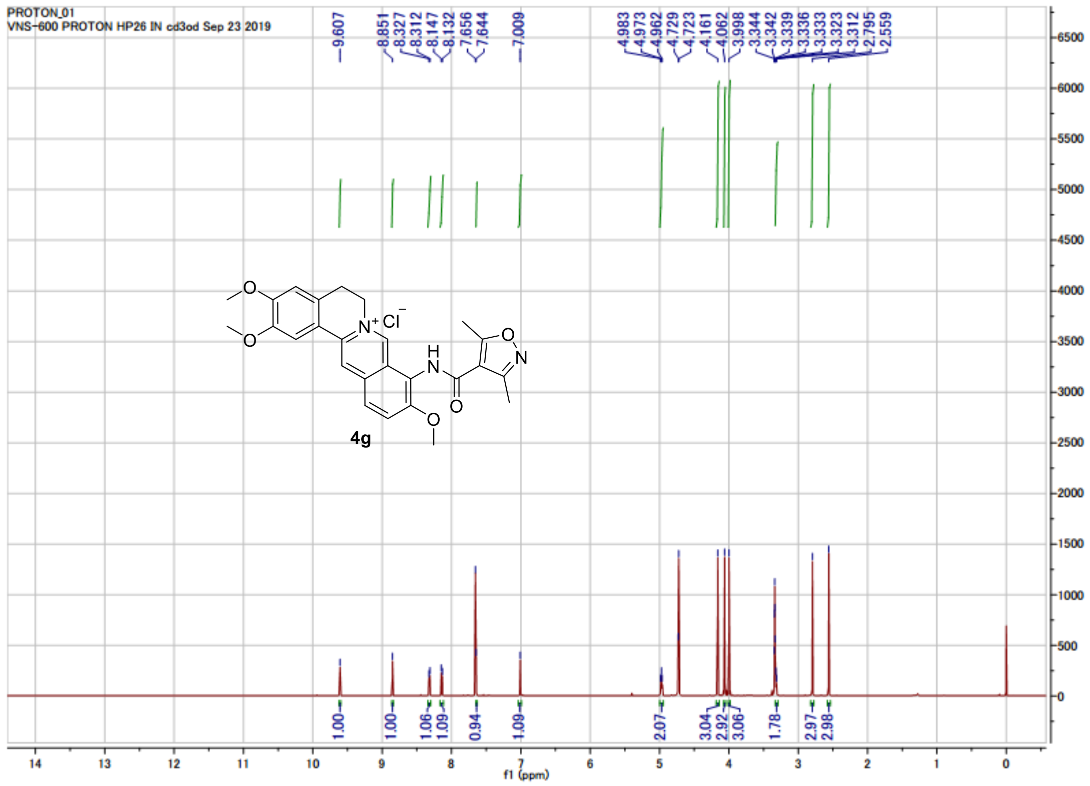

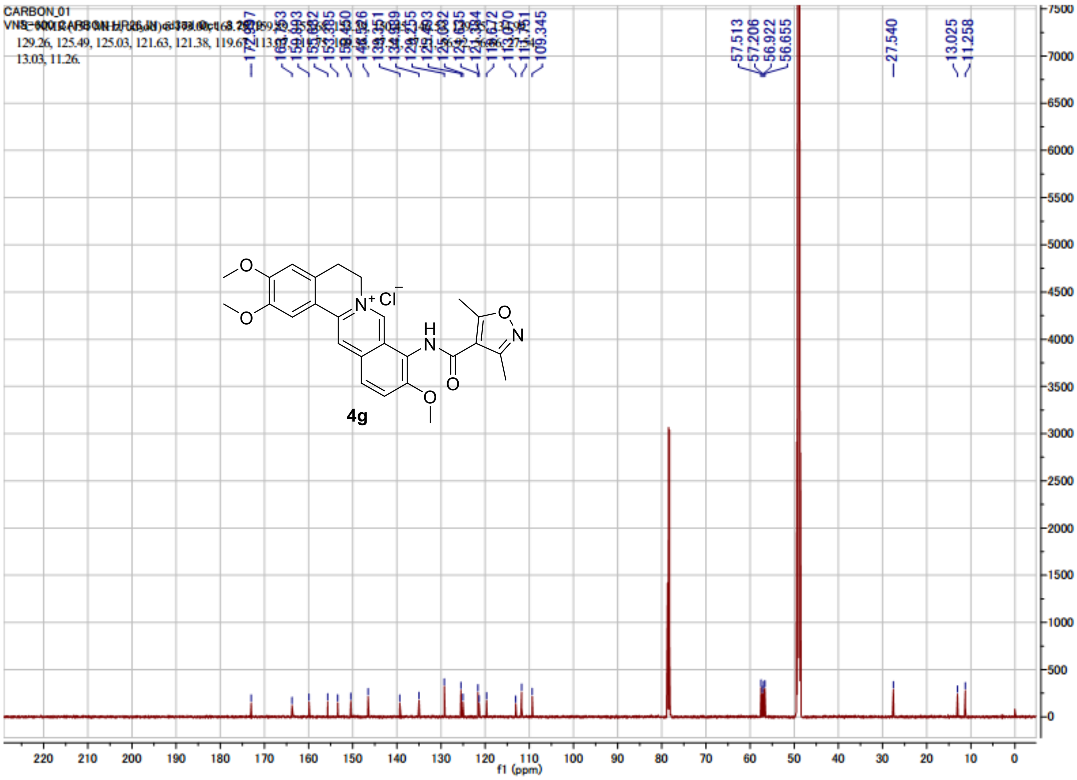

E:\HRMS\2019\10\14\HP26_191014141139 10/14/2019 2:16:40 PM

HP26_191014141139 #34 RT: 0.31 AV: 1 NL: 4.21E8
T: FTMS + c ESI Full ms [150.00-1000.00]

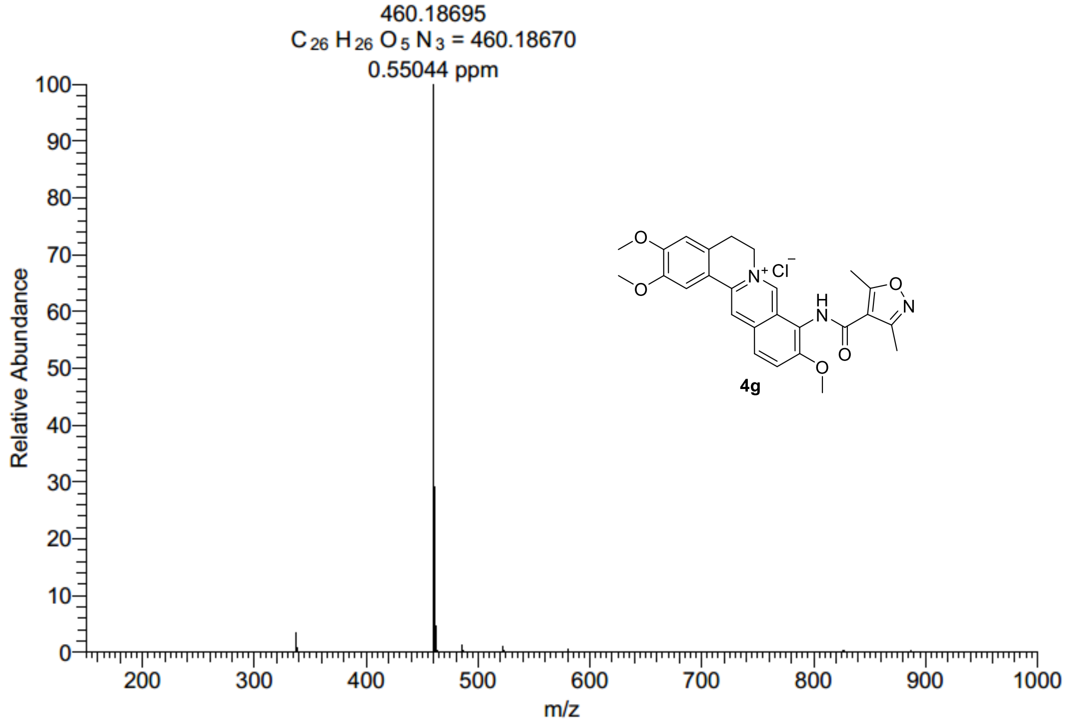

Figure S19: ^{1}H NMR, ^{13}C NMR, HRMS-ESI spectra of compound **4h**

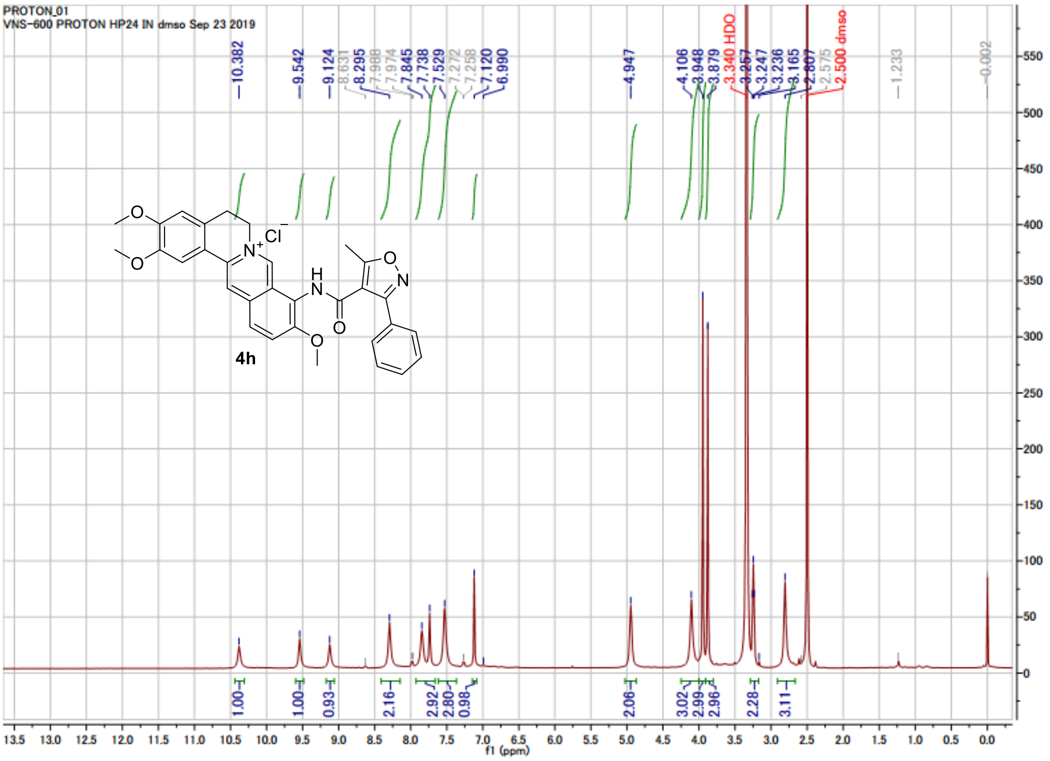

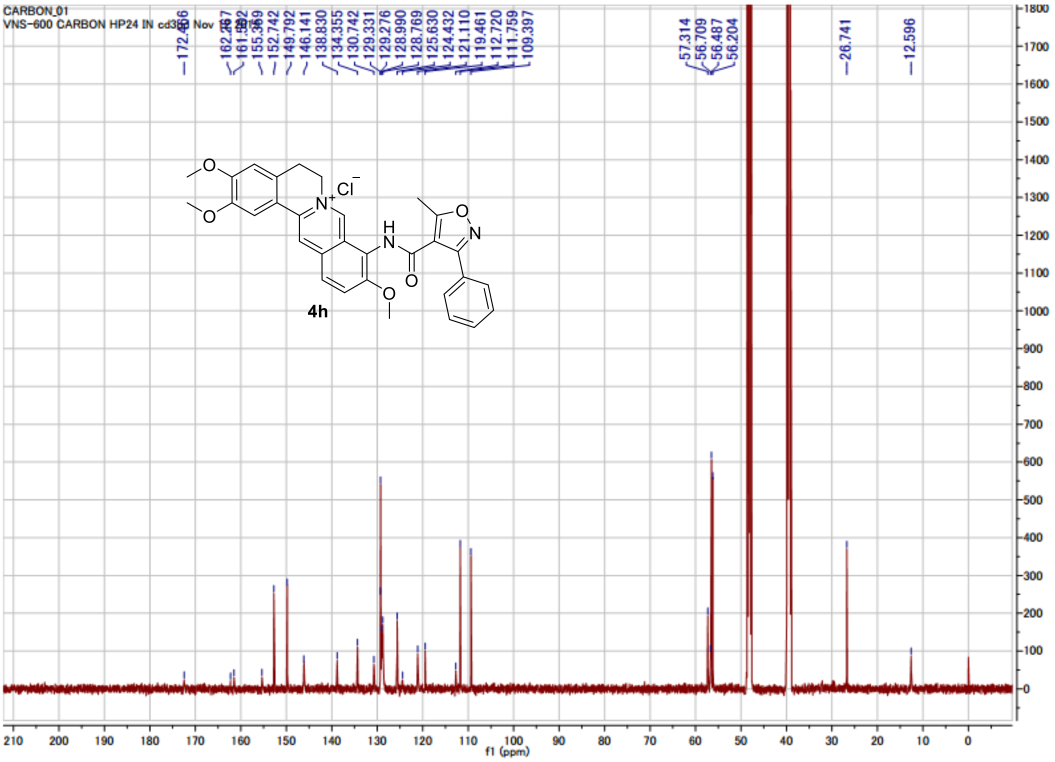

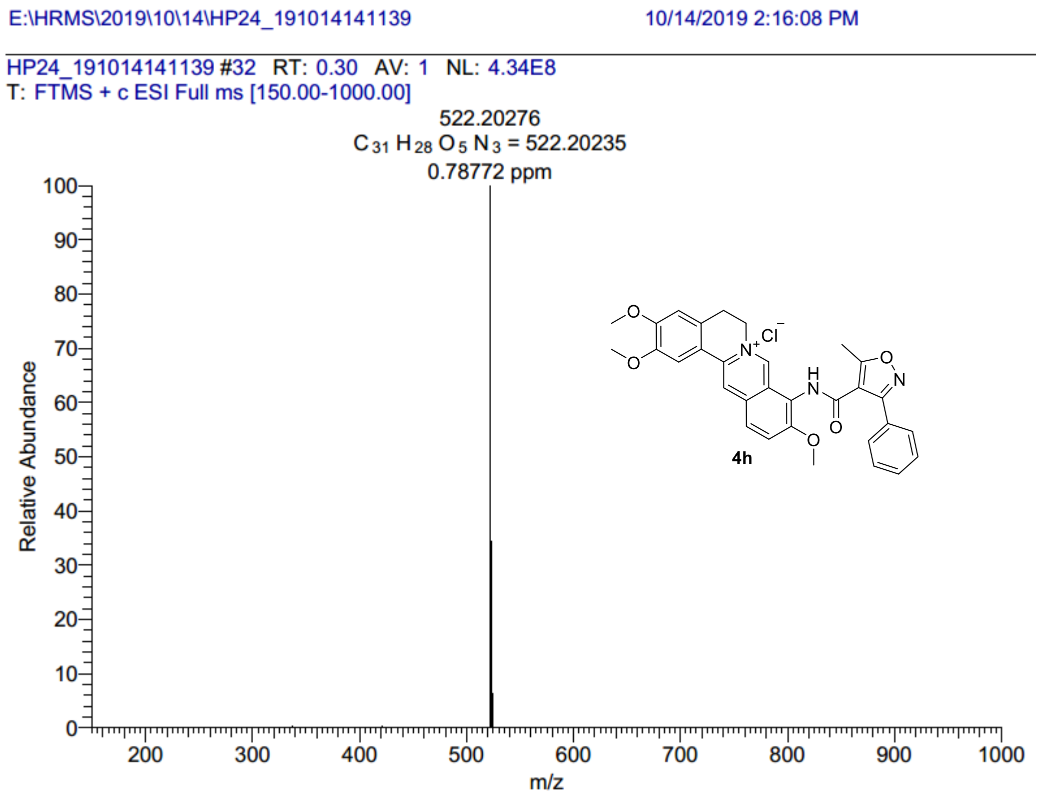

Figure S20: ^{1}H NMR, ^{13}C NMR, HRMS-ESI spectra of compound **4i**

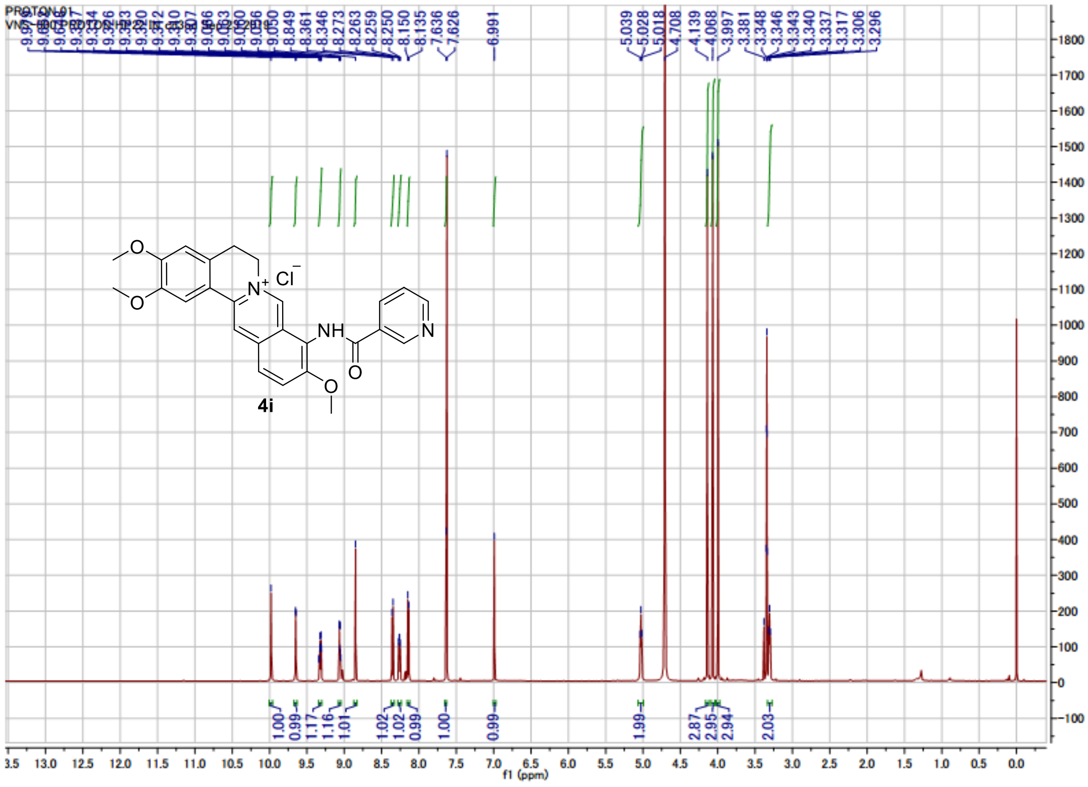

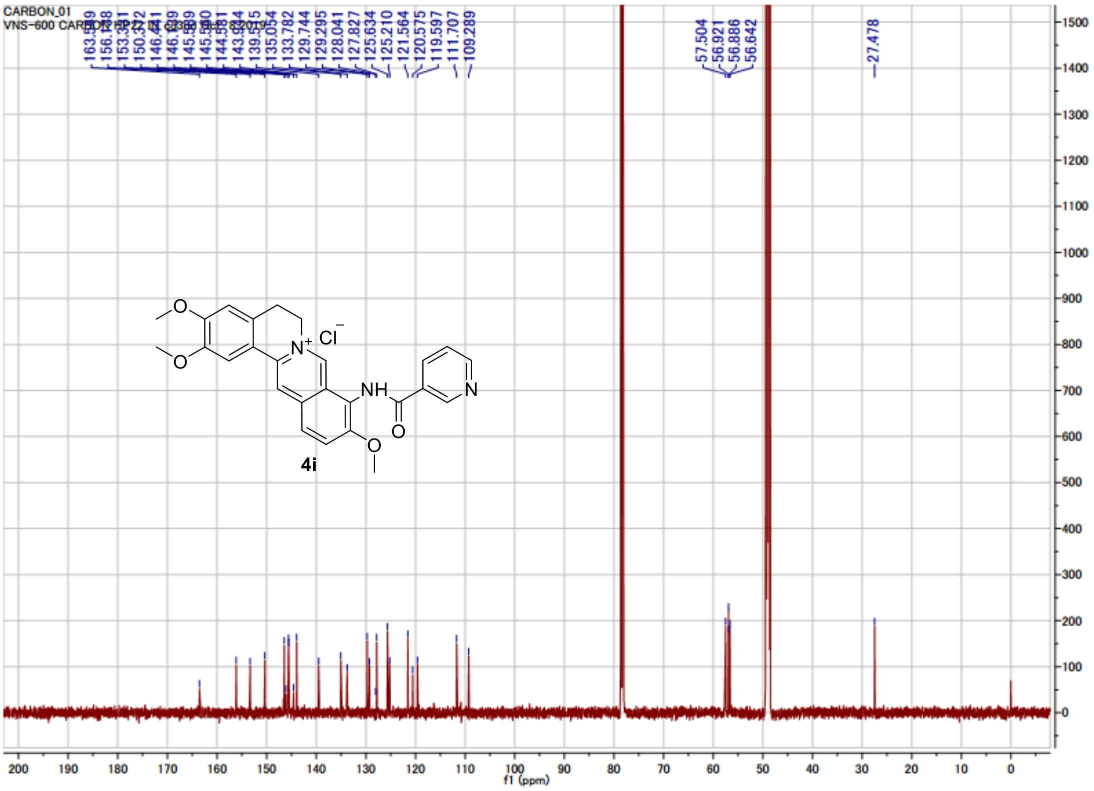

E:\HRMS\2019\10\14\HP22_191014141139 10/14/2019 2:15:38 PM

HP22_191014141139 #32 RT: 0.31 AV: 1 NL: 1.30E8
T: FTMS + c ESI Full ms [150.00-1000.00]

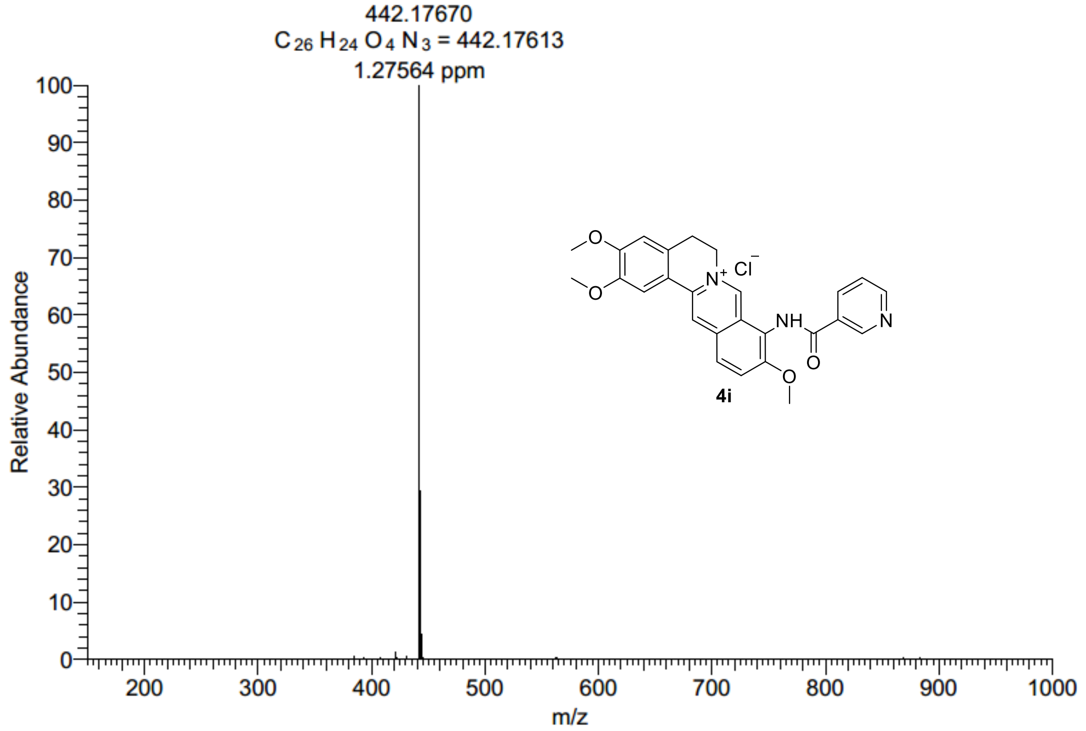

Supplement: Supplementary file 1 [file molecules-25-01352-s001.pdf]
